# Synthesis of some potent immunomodulatory and anti-inflammatory metabolites by fungal transformation of anabolic steroid oxymetholone

**DOI:** 10.1186/1752-153X-6-153

**Published:** 2012-12-10

**Authors:** Naik Tameen Khan, Marium Bibi, Sammer Yousuf, Izhar Husain Qureshi, Atta-ur-Rahman, Abdullah Mohammad Al-Majid, Muhammad Ahmed Mesaik, Ahmed Shukralla Khalid, Samina A Sattar, Atia-tul-Wahab, M Iqbal Choudhary

**Affiliations:** 1 H. E. J. Research Institute of Chemistry, International Center for Chemical and Biological Sciences, University of Karachi, Karachi, 75270, Pakistan; 2 Dr. Panjwani Center for Molecular Medicine and Drug Research, International Center for Chemical and Biological Sciences, University of Karachi, Karachi, 75270, Pakistan; 3 Department of Chemistry, College of Sciences, King Saud University, Riyadh, 11451, Saudi Arabia

**Keywords:** Oxymetholone, Anabolic steroid, Biotransformation, *de novo* Hydroxylation, Immunomodulation, T-Cell proliferation inhibition, Anti-inflammation, Inhibition of reactive oxygen species production, 3T3 Fibroblast cells

## Abstract

**Background:**

Biotransformation of organic compounds by using microbial whole cells provides an efficient approach to obtain novel analogues which are often difficult to synthesize chemically. In this manuscript, we report for the first time the microbial transformation of a synthetic anabolic steroidal drug, oxymetholone, by fungal cell cultures.

**Results:**

Incubation of oxymetholone (**1**) with *Macrophomina phaseolina*, *Aspergillus niger*, *Rhizopus stolonifer*, and *Fusarium lini* produced 17β-hydroxy-2-(hydroxy-methyl)-17α-methyl-5α-androstan-1-en-3-one (**2**), 2α,17α-di(hydroxyl-methyl)-5α-androstan-3β,17β-diol (**3**), 17α-methyl-5α-androstan-2α,3β,17β-triol (**4**), 17β-hydroxy-2-(hydroxymethyl)-17α-methyl-androst-1,4-dien-3-one (**5**), 17β-hydroxy-2α-(hydroxy-methyl)-17α-methyl-5α-androstan-3-one (**6**), and 2α-(hydroxymethyl)-17α-methyl-5α-androstan-3β-17β-diol (**7**). Their structures were deduced by spectral analyses, as well as single-crystal X-ray diffraction studies. Compounds **2**–**5** were identified as the new metabolites of **1**. The immunomodulatory, and anti-inflammatory activities and cytotoxicity of compounds **1**–**7** were evaluated by observing their effects on T-cell proliferation, reactive oxygen species (ROS) production, and normal cell growth in MTT assays, respectively. These compounds showed immunosuppressant effect in the T-cell proliferation assay with IC_50_ values between 31.2 to 2.7 μg/mL, while the IC_50_ values for ROS inhibition, representing anti-inflammatory effect, were in the range of 25.6 to 2.0 μg/mL. All the compounds were found to be non-toxic in a cell-based cytotoxicity assay.

**Conclusion:**

Microbial transformation of oxymetholone (**1**) provides an efficient method for structural transformation of **1**. The transformed products were obtained as a result of *de novo* stereoselective reduction of the enone system, isomerization of double bond, insertion of double bond and hydroxylation. The transformed products, which showed significant immunosuppressant and anti-inflammatory activities, can be further studied for their potential as novel drugs.

## Background

Microbial regio- and stereo-selective transformations of steroids have been extensively investigated [1,2]. We have been studying microbial transformation of bioactive steroids with the objectives of producing their novel metabolites and understanding their metabolism [3-8]. Oxymetholone [17β-hydroxy-2-(hydroxymethylene)-17α-methyl-5α-androstan-3-one, C_21_H_32_O_3_ (**1**), a 17α-alkylated anabolic-androgenic steroid, has been approved by the US Food and Drug Administration for the treatment of blood anemia, osteoporosis, HIV-associated wasting, antithrombin III deficiency, pediatric growth impairment, and damaged myocardium; as well as for stimulating muscle growth in malnourished, and underdeveloped individuals. However, it is also been abused by some athletes for enhancing muscle mass and strength [9,10]. Compound **1** also known to exhibit immunosuppressant activity *in vivo* by decreasing the activity of T-cells [11].

During current study, compound **1** was found to exhibit significant immunomodulatory and anti-inflammatory activities *in vitro* in T-cell proliferation and reactive oxygen species (ROS) production assay, respectively. T-Lymphocytes play a key role in cell mediated immune response by activating various T-cells and modulating autoimmune response. Thus inhibition of T-cell proliferation can serve as an approach to treat various immune disorders [12], including organ rejection after transplant [13]. The immune system also utilizes various cellular processes for elimination of pathogens, such as phagocytosis which involves elimination of pathogens by enzyme catalyzed oxidative burst. Ironically prolonged overproduction of ROS can damage body’s own cells and tissues, and lead to chronic inflammation and other autoimmune diseases [14].

Based on these preliminary findings, we investigated biotransformation of **1** to obtain new analogues. Preliminary experiments showed that *Macrophomina phaseolina*, *Aspergillus niger*, *Rhizopus stolonifer*, and *Fusarium lini* can efficiently transform **1** into several metabolites. Subsequent large scale fermentation was carried out and four new metabolites **2**–**5**, along with two known metabolites **6** and **7** were obtained. The structures of new metabolites were unambiguously deduced through 1D- and 2D-NMR and by single-crystal X-ray diffraction techniques. Metabolites **2**–**7** were then evaluated for the inhibition of T-cell proliferation (IC_50_ values in the range of 31.2 to 2.7 μg/mL), and ROS production (**1** and **2** had a strong inhibition (IC_50_ = 2 to 2.3 μg/mL)), representing their immunosuppressant and anti-inflammatory potential.

## Results and discussion

### Structure elucidation of metabolites

**Figure 1:**
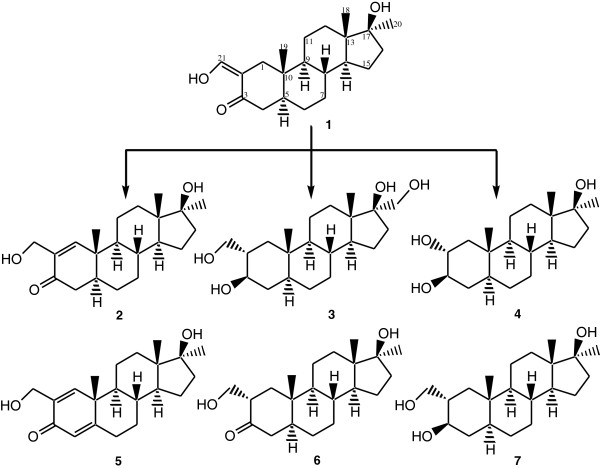
**Biotransformation of oxymetholone(1)by *Macrophomina phaseolina *(compounds 2**, **3**, **6**, **7)**, ***Aspergillus niger *(compounds 6**, **7)**, ***Rhizopus stolonifer *(compounds 3**, **6)and *Fusarium lini *(compounds 2**, **4**, **5).**

Incubation of oxymetholone (**1**) (C_21_H_32_O_3_) with *Macrophomina phaseolina*, *Aspergillus niger*, *Rhizopus stolonifer* and *Fusarium lini* produced six metabolites **27** (Figure 1). Metabolites **6** and **7** were previously obtained through chemical hydrogenation of **1**[15], however this is the first report of their biomimetic synthesis. The structures of new metabolites **25** were unambiguously deduced largely by single-crystal X-ray diffraction analyses.

**Figure 2:**
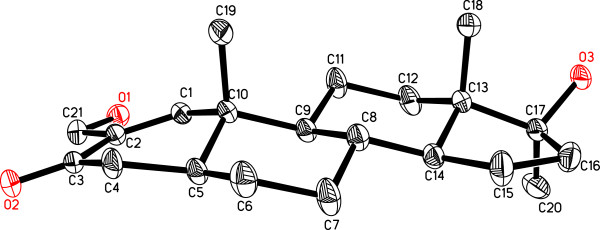
**Computer**-**generated ORTEP diagram of metabolite 2(Hydrogens are omitted for clarity).**

The HREI-MS *M*^+^ = *m*/*z* 332.2333 (calcd 332.2351) (C_21_H_32_O_3_)], IR (1666 cm^-1^) and UV (236.4 nm) spectra of **2** suggested the C = C isomerization of **1** into **2**[8,16]. The ^1^H- and ^13^C-NMR spectra (Table 1) showed a new olefinic methine H/C signal at δ 6.48/δ 152.4, along with a hydroxyl-bearing methylene H_2_/C at δ 4.78 and 4.76 (*J*_21a,b_ = 15.0 Hz)/δ 59.8 for HC-1 and H_2_C-21, respectively. Me (19) protons (δ 0.96) were correlated with C-1 (δ 152.4) in HMBC, while C-21 protons (δ 4.78, 4.76) were correlated with C-1 (δ 152.4) and C-2 (δ 137.5). Additionally, C-1 (δ 6.48) and C-4 protons (δ 2.47, 2.26) were also correlated with C-3 (δ 199.0), and C-2 (δ 137.5) C-1 Proton also showed HMBC correlations with C-21 (δ 59.8). The structure of new metabolite **2** was finally deduced through single-crystal X-ray diffraction techniques (Figure 2). The asymmetric unit contains two independent molecules of metabolite **2**. The ORTEP diagrams of **2** (Figure 2) showed four *trans* fused rings A, B, C, and D with *half chair*/*chair*/*chair*, and *envelop* conformations, respectively. C-17 -OH and methyl groups exist in *pseudoequatorial* and *pseudoaxial* orientations, respectively. The shorter bond lengths of C-2—C-3 single-bond (1.476(8) Å) is due to the conjugation of C-1—C-2 (1.321(7) Å) olefinic bond with the C-3 carbonyl moiety. All bond angles and lengths were within the normal range.

**Table 1:** ^**1**^**H- and**^**13**^**C-NMR Chemical Shifts of New Compounds 2–5 (δ in ppm; *J *** **and *W***_***1*** **/** ***2***_**in Hz)**

**Position**	**2**		**3**		**4**		**5**	
	^**1**^**H**	^**13**^**C**	^**1**^**H**	^**13**^**C**	^**1**^**H**	^**13**^**C**	^**1**^**H**	^**13**^**C**
1	6.48, s	152.4	1.63; 1.57, m	36.5	2.28, dd (12.5, 4.5); 1.30, m	46.6	7.45, s	150.0
2	-	137.5	1.98, m	40.5	4.05, m (*W*_1/2_ = 20.6 )	73.1	-	137.6
3	-	199.0	4.51, br. s (*W*_1/2_ 12.0)	67.6	3.86, m (*W*_1/2_ = 20.6 )	76.7	-	186.0
4	2.47, dd (17.3, 4.2), 2.26, dd (17.3, 4.0)	41.6	1.71; 1.60 m	37.7	1.87 ddd (13.0, 5.0, 2.5); 1.72, m	37.2	6.28, s	124.0
5	1.83, m	44.8	2.01, m	39.6	1.26, m	45.3	-	169.4
6	1.32; 1.27, m	27.6	1.31; 1.25, m	28.8	1.28; 1.22, m	28.4	2.32, 2.21, m	32.5
7	1.71; 0.85, m	31.5	1.65; 0.90, m	32.4	1.65; 0.85, m	32.2	1.75; 0.85, m	33.7
8	1.44, m	36.7	1.44, m	36.4	1.37, m	35.9	1.57, m	36.3
9	0.78, m	50.7	0.76, m	54.9	0.67, m	54.7	0.81, m	52.9
10	-	38.9	-	36.6	-	37.6		43.5
11	1.63; 1.37, m	21.3	1.57; 1.29, m	21.0	1.62; 1.33, m	21.4	1.65; 1.55, m	22.9
12	1.61; 1.22, m	32.2	1.78; 1.49, m	32.7	1.58, 1.35, m	32.0	1.53; 1.14, m	31.8
13	-	46.3	-	46.1	-	46.2	-	46.2
14	1.20, m	51.2	1.37, m	51.8	1.24, m	51.0	1.12, m	50.2
15	1.53; 1.24, m	23.7	1.57; 1.34, m	24.2	1.54; 1.29, m	23.8	1.48; 1.30, m	23.8
16	2.14, ddd (13.5, 11.7, 3.5); 1.77, m	39.5	2.30, ddd (15.6, 9.2, 6.7); 1.95, m	34.1	2.13; 1.76, m	39.4	2.14, td (13.0, 3.5); 1.78, m;	39.2
17	-	80.5	-	83.2	-	80.6	-	80.3
18	1.08, s	14.9	1.14, s	15.2	1.06, s	14.8	1.08, s	14.8
19	0.96, s	13.1	0.87, s	12.5	0.89, s	13.7	1.15, s	18.8
20	1.41. s	26.8	4.09; 3.82, d (10.3)	67.3	1.40, s	26.7	1.37, s	26.6
21	4.78; 4.76, d (15.0)	59.8	4.17; 4.03, m	66.1	-	-	5.06; 4.98, m	59.5

**Figure 3:**
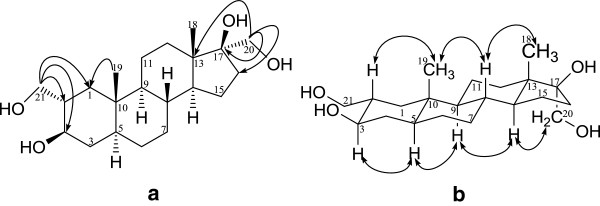
**Important** (**a**) **HMBC**, **and** (**b**) **NOESY correlations in metabolite 3.**

The UV inactive metabolite **3** showed molecular ion (M^+^) at *m*/*z* 352.2643 (calcd 352.2613), in agreement with the formula C_21_H_36_O_4_, indicating reduction of C = C bond and addition of any oxygen. The ^1^H- and ^13^C-NMR spectra of **3** (Table 1) showed two pairs of downfield OH-bearing methylene H_2_/C signals at δ 4.09, 3.82 (d, *J*_20a,b_ = 10.3 Hz)/δ 67.3 (H_2_C-20) and 4.17, 4.03 (m)/δ 66.1 (H_2_C-21), along with an OH-bearing methine H/C at δ 4.51 (br. s, *W*_1/2_ = 12.0 Hz)/δ 67.6 (HC-3). The H_2_C-21 (δ 4.17, 4.03) and HC-3 (δ 4.51) both showed COSY correlations with the vicinal HC-2 (δ 1.98). Moreover, C-21 protons showed HMBC correlations with C-1 (δ 36.5), C-2 (δ 40.5) and C-3 (δ 67.6) (Figure 3a). Similarly, C-20 methylene protons at δ 4.09, 3.82 exhibited HMBC interactions with C-13 (δ 46.1), C-16 (δ 34.1), and C-17 (δ 83.2). Stereochemistry at C-2 and C-3 was deduced from the NOE correlations of HC-2 (δ 1.98) with Me-19 (δ 0.87), and HC-3 (δ 4.51) with HC-5 (δ 2.01) (Figure 3b). On the basis of above observations, the new metabolite was characterized as 2α,17α-di(hydroxymethyl)-5α-androstan-3β-17β-diol (**3**).

**Figure 4:**
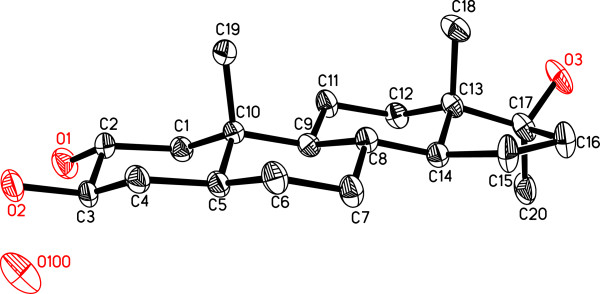
Computer-generated ORTEP diagram of metabolite 4 (Hydrogens are omitted for clarity).

The formula C_20_H_34_O_3_ for **4** (*M*^+^ = *m*/*z* 322.2507, calcd 322.2508) indicated four double bond equivalents and loss of oxidative carbon. The UV and IR spectra indicated the absence of enone functionality. The ^1^H- and ^13^C-NMR (Table 1) of metabolite **4** was substantially different from that of the substrate **1**, first due to the lack of HC-21 olefinic methine signal, and second the appearance of two new hydroxyl-bearing methine H/C signals at δ 4.05 (m, *W*_1/2_ = 20.6 Hz)/δ 73.1 and 3.87 (m, *W*_1/2_ = 20.6 Hz)/δ 76.7. Vicinal correlations between H_2_C-1 (δ 2.27, 1.30), HC-2 (δ 4.05), HC-3 (δ 3.87) and H_2_C-4 (δ 1.88, 1.72) in the COSY 45° spectrum indicated the oxidative loss of C-21 methine. HC-2 (δ 4.05) and HC-3 (δ 3.87) were also correlated to C-1 (δ 46.5), C-4 (δ 37.2), C-5 (δ 45.4), and C-10 (δ 37.6) in the HMBC spectrum. The structure of new metabolite **4** was unambiguously established by single-crystal X-ray diffraction analysis as half water solvate (Figure 4). It showed four *trans* fused rings A, B, C, and D exist in *chair*/ *chair*/*chair*, and *envelop* conformations, respectively. The C-2 and C-3 vicinal diols adopt equatorial orientations, whereas hydroxyl and methyl substituents at C-17 were found in *pseudoequatorial* and *pseudoaxial* orientations, respectively. All bond angles and lengths were in agreement with other related steroidal structures [8].

**Figure 5:**
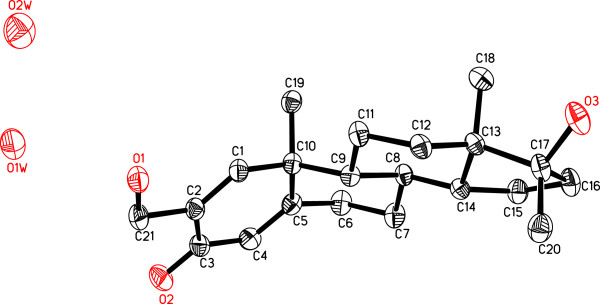
**Computer**-**generated ORTEP diagram of metabolite 5(Hydrogens are omitted for clarity).**

The molecular composition C_21_H_30_O_3_ was deduced from the HREI-MS of metabolite **5** (*M*^+^ = *m*/*z* 330.2183, calcd 330.2195), which was 2 amu less than the metabolite **2**. The UV spectrum of **5** exhibited a λ_max_ at 249.8 nm due to extended conjugation in ring A. The ^1^H- and ^13^C-NMR spectra (Table 1) of metabolite **5** was distinctly similar to **2**, indicating the presence of two olefinic H/C at δ 7.45 (s)/δ 150.0 (HC-1) and 6.28 (d, *J* = 1.0 Hz)/δ 124.0 (HC-4) in ring A, along with hydroxy-methylene H_2_/C signals at δ 5.06, 4.98 (m)/δ 59.5 (H_2_C-21). The methine signals δ 6.28/δ 124.0 and methylene signals δ 5.06, 4.98/δ 59.5 showed similar HMBC correlations as those of HC-1 and H_2_C-21 for metabolite **2**. Similarly, C-4 proton (δ 6.28) was correlated to C-3 (δ 186.0), C-5 (δ 169.4), C-6 (δ 32.5) and C-10 (δ 43.5) in HMBC spectrum. The structure of metabolite **5** was unambiguously deduced by single-crystal X-ray diffraction studies (Figure 5). The asymmetric unit contains two water solvated independent molecules of metabolite **5**. The ORTEP diagrams (Figure 5) showed that compound **5** is consisting of four fused rings A , B, C and D. *Trans* fused rings B, C and D adopt *chair*/*chair* and *envelop* conformations, respectively, with the *pseudo equatorial* orientation of hydroxyl substituent at C-17. Ring A was found to be planner in geometry due to extended conjugation. All the bond angles and lengths were within the normal range as observed in previously reported steroids [8].

**Figure 6:**
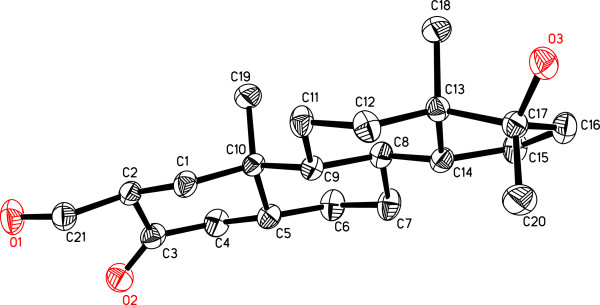
**Computer**-**generated ORTEP diagram of metabolite 6(Hydrogens are omitted for clarity).**

The structure of known metabolite **6** was unambiguously deduced through single-crystal X-ray diffraction studies. ORTEP diagrams (Figure 6) showed that it consists of *trans* fused rings A, B, C, and D with *chair*/*chair*/*chair* and *envelop* conformations, respectively. The hydroxymethylene moiety attached to C-2, and OH at C-17 were found in *equatorial* and *peudo equatorial* orientations, respectively. All the bond angles and lengths were found within normal range. Known metabolite **7** was structurally identified by comparing of its spectral data with the one reported earlier.

### T-Cell proliferation inhibitory activity

Oxymetholone (**1**) is known to modulate cell-mediated immunity *in vivo*. This was in part due to a decrease in T-cell activity [5]. This initial observation prompted us to investigate the T-cell proliferation inhibitory potential of **1** and its metabolites.

**Figure 7:**
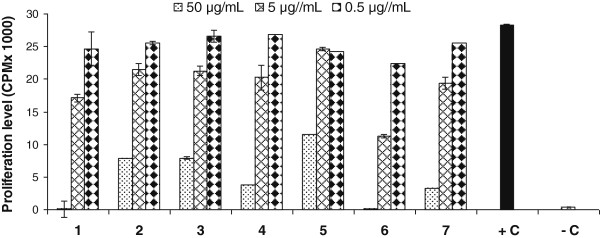
**Effect of compounds 1–7 on phytohemagglutinin** (**PHA**) **activated T**-**cell proliferation.** The effect of compounds on the T-cell proliferation is compared with control. Each bar represents the mean value of triplicate reading ± SD.

Compounds **1**–**7** were evaluated for their effect on T-cell proliferation by employing PHA to activate human peripheral mononuclear cells (PBMC), isolated from the blood sample from healthy human volunteers. The results indicate that metabolite **6** possess more potent T-cell proliferation inhibitory activity (IC_50_ = 2.7 μg/mL), as compared to the substrate **1** (IC_50_ = 7.5 μg/mL) and standard prednisolone (IC_50_ < 3.1 μg/ mL) (Table 2, Figure 7); whereas compounds **7** and **4** showed a moderate inhibitory activity (IC_50_ = 10.6 ± 0.4 and 11.8 ± 0.9 μg/mL, respectively). Compounds **2**–**5** showed a weak inhibition of T-cell proliferation as compared to compounds mentioned earlier. Limited SAR indicated that greater flexibility in ring A, due to reduction of C = C or C = O bonds probably contributes in activity.

**Table 2:** Inhibitory effect of compounds on the T-cell proliferation in comparison to standard

**Compound**	**IC**_**50**_**μg**/**mL**^a^)
**1**	7.5 ± 0.4
**2**	17.4 ± 1.2
**3**	17.0 ± 1.2
**4**	11.8 ± 0.9
**5**	31.2 ± 1.9
**6**	2.7 ± 0.2
**7**	10.6 ± 0.4
**Standard** (Prednisolone)	< 3.1

^a^)Each IC_50_ value represents the mean value of triplicate reading ± SD.

### ROS inhibition activity

**Figure 8:**
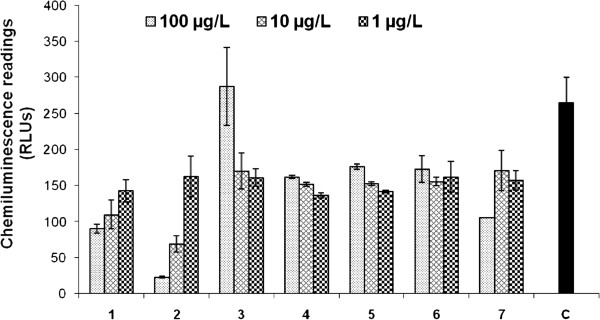
**Effect of compounds 1**–**7 on reactive oxygen species** (**ROS**) **production using whole blood phagocytes.** The compounds activity was compared with the control (C = cells with activator). Each plot and error bar represents reading ± SD of three repeats.

**Figure 9:**
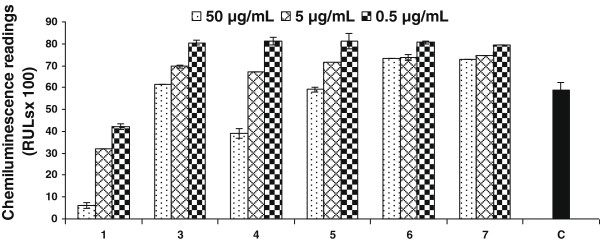
**Effect of compounds 1 and3**–**7 on reactive oxygen species** (**ROS**) **production using isolated neutrophils.** The compounds activity was compared with the control (C = cells with activator). Each plot and error bar represents reading ± SD of three repeats.

Compounds **1**–**7** were also investigated for their effect on ROS production by using whole blood and professional phagocytic PMNs and the detecting probes luminal (Table 3, Figures 8 and 9). Compound **2** showed slightly stronger (p ≤ 0.005) inhibition (IC_50_ = 2.0 μg/mL) as compared to **1** (IC_50_ = 2.3 μg/mL). Both compounds were at least five fold more active than the standard ibuprofen (IC_50_ = 11.2 μg/mL) in whole blood phagocytes. Metabolite **7** showed a moderate inhibitory activity (IC_50_ = 25.6 μg/mL), while other compounds did not show any activity even at the highest concentration (100 μg/mL). Compounds which showed potent inhibitory activities were further evaluated by using professional phagocytes PMNs. Compound **1** showed a significant inhibition of ROS generation in the PMNs (IC_50_ = 6.3 μg/mL). Interestingly ROS generation inhibitory activity of compound **1** and its known metabolites **6** and **7** have not been studied before.

**Table 3:** Inhibitory effects of compounds on ROS production in human whole blood and PMNs

**Compound**	**IC**_**50**_**μg**/**mL**^**a**^) (**Whole Blood phagocytes**)	**IC**_**50**_**μg**/**mL**^**a**^) (**PMNs**)
**1**	2.3 ± 0.0	6.3 ± 0.1
**2**	2.0 ± 0.8	-
**3**	>100	>50
**4**	>100	>50
**5**	>100	>50
**6**	>100	>50
**7**	25.6	>50
**Standard** (Ibuprofen)	11.2 ± 1.9	2.5 ± 0.6

^a^)Each IC_50_ value represents the mean value of triplicate reading ± SD.

### Cytotoxicity

Compounds **1**–**7** were found to be non-toxic towards the normal mouse fibroblast (3T3) cells, even at the highest concentrations tested (100 μg/mL).

## Conclusions

Our study provides an efficient method for the production of new anabolic steroids by the structural transformation of oxymetholone (**1**) by using fungi. The procedure presented here can also be used for the study of the metabolism of oxymetholone (**1**), as well as for the production of potential immunomodulatory and anti-inflammatory drugs. In the current study, compounds **2**–**7** found to posses a strong inhibitory effect on T-cell proliferation, with IC_50_ values between 31.2 to 2.7 μg/mL, while in case of the ROS production, only compounds **1** and **2** exerted significant inhibition (IC_50_ ~ 2.0 μg/mL) on whole blood phagocytes. Whereas only **1** showed significant ROS inhibition (IC_50_ = 6.3 μg/mL) on the isolated PMNs.

## Experimental

### General experimental conditions

Silica gel precoated plates (Merck, PF_254_; 20 × 20, 0.25 mm, Germany) were used for the TLC based separation. Silica gel (70–230 mesh, Merck) was used for column chromatography. Melting points were determined with a Buchi-535, apparatus and are uncorrected. Optical rotations were measured in methanol with a JASCO P-2000 polarimeter. UV Spectra (in nm) were recorded in methanol with a Hitachi U-3200 spectrophotometer. Infrared (IR) spectra (in cm^-1^) were recorded with an FT-IR-8900 spectrophotometer. ^1^H- and ^13^C-NMR spectra were recorded in C_5_D_5_N on a Bruker Avance NMR spectrometer, with residual solvent signal as the internal standard. Standard Bruker pulse sequences were used for 1D- and 2D-NMR experiments. The chemical shifts (δ values) are reported in parts per million (ppm), relative to TMS at 0 ppm. The coupling constants (*J* values) are reported in Hertz. Electron impact (EI-MS), and high-resolution mass spectra (HREI-MS) were recorded on JEOL JMS-600H mass spectrometer (Japan); in *m*/*z* (rel.%). Single-crystal X-ray diffraction data were collected on a Bruker Smart APEX II diffractometer with CCD detector [17]. Data reductions were performed by using SAINT program. The structures were solved by direct methods [18], and refined by full-matrix least squares on F2 by using the SHELXTL-PC package [19]. The figures were plotted with the aid of ORTEP program [20]. The luminometer used was from Luminoskan RS (Labsystem Luminoskan, Helsinki, Finland), and cell harvester and glass fiber filters used were from Inotech (Dottikon, Swetzerland). Liquid scintillation counter used was LS65000 from Beckman Coulter (Fullerton, CA, USA). Microplate reader used was SpectraMax (Molecular Devices, CA, USA).

The chemicals and reagents were purchased from the following sources: Oxymetholone (**1**) (TCI, Japan), Luminol (Research Organics, OH, USA), Hanks balance salts solution (HBSS), phytohemagglutinin-L (PHA-L), penicillin, and streptomycin (Sigma,St. Louis, USA), lymphocytes separation medium (LSM) (MP Biomedicals, Illkirch, France), zymosan-A (*Saccharomyces cerevisiae*) (Fluka BioChemika, Buchs, Switzerland), tritiated thymidine (Amersham Pharmacia Biotech, UK) and 3-[4,5-dimethylthiazol-2-yl]-2,5-diphenyl-tetrazolium bromide (MTT) (Amresco, Solon, OH, USA).

Tissue culture plates were obtained from Iwaki (Japan). Mouse fibroblasts (3T3) were obtained from the European American Culture Collection (EACC). Dulbcco’s modified eagle medium (DMEM) and fetal bovine serum were purchased from Gibco-BRL (Grand Island, NY, USA).

### Microorganisms and culture medium

The fungi were obtained either from the Northern Regional Research Laboratories (NRRL), or Karachi University Culture Collection (KUCC) or American Type Culture Collection (ATCC).

*Macrophomina phaseolina* (KUCC 730) and *Fusarium lini* (NRRL 2204) were grown in a medium composed of the following ingredients and dissolved in distilled H_2_O (4.0 L): glucose (40.0 g), glycerol (40.0 mL), peptone (20.0 g), yeast extract (20.0 g), KH_2_PO_4_ (20.0 g), and NaCl (20.0 g). The media (2.0 L) for *Aspergillus niger* (ATCC 10549), and *Rhizopus stolonifer* (ATCC 10404) were the same as above, except addition of glycerol (10.0 mL) for *A*. *niger* and yeast extract (6.0 g) for *R*. *stolonifer*.

### General fermentation and extraction conditions

The fungal medium was distributed into 250 mL conical flasks (100 mL each) and autoclaved at 121°C. Mycelia of the fungi were transferred to flasks and incubated at 26 ± 2°C for two-three days on rotary shaking (120 rpm). Compound **1**, dissolved in acetone, was evenly distributed among all the flasks which were placed on the rotary shaker (120 rpm) at 26 ± 2°C for fermentation. Parallel control experiments were conducted which included an incubation of the fungus without substrate **1**, and an incubation of **1** in the medium without fungus. The degree of transformation was analyzed on TLC after one day. The culture medium and mycelium were separated by filtration. The mycelium was washed with dichloromethane (CH_2_Cl_2_, 1.0 L). The aqueous filtrate was extracted with CH_2_Cl_2_ (4 L × 3). The CH_2_Cl_2_ extract was dried over anhydrous Na_2_SO_4_, evaporated under reduced pressures, and the resulting brown gum was analyzed by thin-layer chromatography. The control flasks were also harvested in the same manner, and compared with the test to assure the presence of biotransformed products.

### Fermentation of oxymetholone (1) with *Macrophomina phaseolina*

Oxymetholone (**1**; 800 mg) was dissolved in 20 mL acetone, and uniformly distributed to 40 flasks containing 2 days old *M*. *phaseolina* culture. Fermentation was carried out for twelve days. The gummy material (2.5 g), obtained after filtration, extraction and evaporation, was loaded onto a silica gel column for fractionation. The mobile phase was composed of pet. ether and acetone with a gradient of 10%. Three main fractions (OX-1 − 3) were obtained on the basis of TLC analysis. Fraction OX-1 yielded metabolites **2** (07 mg) and **6** (43 mg) on elution from silica gel column (pet. ether: acetone = 9:1), while OX-2, when subjected to silica gel column chromatography, yielded metabolite **7** (21 mg, pet. ether: acetone = 8:2). Fraction OX-3 yielded metabolite **3** (276 mg, pet. ether: acetone = 7:3) after elution from silica gel column.

#### 17β-Hydroxy-2-(hydroxymethyl)-17α-methyl-5α-androstan-1-en-3-one (**2**)

Colorless crystalline solid; m.p. 173–174°C; [α]^25^_D_ -45.4 (*c* 0.03, MeOH); UV (MeOH): λ_max_ nm (log *ε*) 236.4 (3.9); IR (KBr): ν_max_: 3358, 1666, 1629, 1095 cm^-1^; ^1^H-NMR: (500 MHz, C_5_D_5_N) see Table 2; ^13^C-NMR: (125 MHz, C_5_D_5_N) see Table 2; EI-MS (%): *m*/*z* 332 (*M*^+^, 30), 274 (25), 216 (20), 176 (39), 174 (52), 161 (34), 147 (24), 123 (32), 108 (31), 91 (56), 71 (100), 55 (95); HREI-MS: *m*/*z* 332.2333 (M^+^ [C_21_H_32_O_3_]^+^, calcd 332.2351). Crystal data: empirical formula = C_21_H_32_O_3_, *Mr* = 332.47, orthorhombic, space group P2_1_2_1_2_1_, *a* = 7.715 (4) Å, *b* = 13.604 (7) Å, *c* = 36.769 (16) Å, *V* = 3859 (3) Å^3^, Z = 8, *ρ*_calc_ = 1.145 mg m^-3^, F(000) = 1456, *μ* (Mo K*α*) = 0.71073 Å, max/min transmission 0.9948/0.9867, crystal dimensions 0.18 × 0.13 × 0.07 mm, 1.11° < *θ* < 25.50°, 22,826 reflections were collected, out of which 4,091 reflections were observed (*R*_int_ = 0.1228) and 433 parameters were refined. The *R*-values were; *R*_1_ = 0.0610, *wR*_2_ = 0.1293 for I > 2*σ* (I), and *R*_1_ = 0.1210, *wR*_2_ = 0.1691 for all data, max/min residual electron density; 0.226/-0.229 e Å^-3^. Crystallographic data for compound **2** can be obtained from the Cambridge Crystallographic Data Center, through the allocated deposition code CCDC 795529 (Additional file 1 and Additional file 2).

#### 2α,17α-Di(hydroxymethyl)-5α-androstan-3β-17β-diol (**3**)

Amorphous material; [α]^25^_D_ -14.3 (*c* = 0.03, MeOH); IR (KBr): ν_max_ 3382, 1381 cm^-1^. ^1^H-NMR: (600 MHz, C_5_D_5_N) see Table 3; ^13^C-NMR: (150 MHz, C_5_D_5_N) see Table 3; EI-MS (%): *m*/*z* 352 (*M*^+^, 6), 334 (26), 316 (14), 303 (72), 285 (100), 260 (55), 245 (49), 229 (23), 177 (23), 161 (33), 147 (37), 107 (47), 93 (42), 55 (26); HREI-MS: *m*/*z* 352.2623 (M^+^ [C_21_H_36_O_4_]^+^, calcd 352.2613) (Additional file 3).

#### 17β-Hydroxy-2α-(hydroxymethyl)-17α-methyl-5α-androstan-3-one (**6**)

Colorless crystalline solid; m.p. 197–199°C. [lit. 198–200°C] [15]; [α]^25^_D_ +13.5 (*c* 0.04, MeOH) [lit. +19.7]. Crystal data: empirical formula = C_21_H_34_O_3_, *Mr* = 334.48, orthorhombic, space group P2_1_2_1_2_1_, *a* = 7.3859 (3) Å, *b* = 20.6898 (9) Å, *c* = 12.4157 (6) Å, *V* = 1897.27 (15) Å^3^, Z = 4, *ρ*_calc_ = 1.171 mg m^-3^, F(000) = 736, *μ* (Mo K*α*) = 0.71073 Å, max/min transmission 0.9962/0.9754, crystal dimensions 0.33 x 0.21 x 0.05 mm, 1.64° < *θ* < 27.50°, 18840 reflections were collected, out of which 4479 reflections were observed (*R*_int_ = 0.0422) and 437 parameters were refined. The *R*-values were; *R*_1_ = 0.0534, *wR*_2_ = 0.1354 for I > 2*σ* (I), and *R*_1_ = 0.0707, *wR*_2_ = 0.1472 for all data, max/min residual electron density; 0.433/-0.214e Å^-3^. Crystallographic data for compound **6** can be obtained from the Cambridge Crystallographic Data Center (code CCDC 795530) (Additional file 4 and Additional file 5).

#### 2α-(Hydroxymethyl)-17α-methyl-5α-androstan-3β-17β-diol (**7**)

Colorless crystalline solid; m.p. 279–281°C. [lit. 280–282°C] [15]; [α]^25^_D_ -25.4 (*c* 0.02, MeOH) [lit. – 37.0] (Additional file 6).

### Fermentation of oxymetholone (1) with *Aspergillus niger* and *Rhizopus stolonifer*

Incubation of **1** (400 mg/10 mL acetone) with 2 days old culture of *A*. *niger* in 20 flasks for 6 days produced the previously isolated metabolites **6** (64 mg) and **7** (136 mg), while *R*. *stolonifer* (20 flasks) transformed **1** (400 mg/10 mL acetone) into metabolites **3** (86 mg) and **6** (15 mg).

### Fermentation of oxymetholone (1) with *Fusarium lini*

Incubation of **1** (600 mg/15 mL acetone) with 2-day old *F*. *lini* culture in 30 flasks for 12 days produced three metabolites which were purified by silica gel column chromatography to obtain metabolites **2** (146 mg), **4** (32 mg, pet. ether: acetone = 7:3) and **5** (15 mg, pet. ether: acetone = 7:3).

#### 17α-Methyl-5α-androstan-2α,3β-17β-triol (**4**)

Colorless crystalline solid; m.p.: 124–125°C. [α]^25^_D_ : -29.0 (*c* = 0.01, MeOH). IR (KBr): ν_max_ 3409, 2927, 1051 cm^-1^; ^1^H-NMR: (500 MHz, C_5_D_5_N) see Table 3; ^13^C-NMR: (125 MHz, C_5_D_5_N) see Table 3; EI-MS (%): *m*/*z* 322 (*M*^+^, 98), 307 (100), 304 (43), 264 (39), 249 (95), 229 (30), 215 (53), 181 (54), 171 (58), 169 (51), 123 (55), 109 (33), 95 (41), 81 (40), 57 (30), 43 (42); HREI-MS: *m*/*z* 322.2507 (M^+^ [C_20_H_34_O_3_]^+^, calcd 322.2508); Crystal data: empirical formula = C_20_H_35_O_4_ [C_20_H_34_O_3_·OH], *Mr* = 339.48, monoclinic, space group P2_1_, *a* = 11.5169 (19) Å, *b* = 6.7843 (12) Å, *c* = 12.820 (2) Å, *V* = 945.6 (3) Å^3^, Z = 2, *ρ*_calc_ = 1.192 mg m^-3^, F(000) = 374, *μ* (Mo K*α*) = 0.71073 Å, max/min transmission 0.9912/0.9785, crystal dimensions 0.27 × 0.12 × 0.11 mm, 1.68° < *θ* < 25.50°, 4,363 reflections were collected, out of which 1,585 reflections were observed (*R*_int_ = 0.0493) and 221 parameters were refined. The *R*-values were; *R*_1_ = 0.0452, *WR*_2_ = 0.0808 for I > 2*σ* (I), and *R*_1_ = 0.0791, *WR*_2_ = 0.0914 for all data, max/min residual electron density; 0.147/-0.141 e Å^-3^. Crystallographic data for compound **4** can be obtained from the Cambridge Crystallographic Data Center (code CCDC 795528) (Additional file 7 and Additional file 8).

#### 17β-Hydroxy-2-(hydroxymethyl)-17α-methylandrost-1,4-dien-3-one (**5**)

Colorless crystalline solid; m.p. 163–164°C; [α]^25^_D_ -33.0 (*c* = 0.01, MeOH); UV (MeOH): λ_max_ nm (log *ε*): 249.8 (4.0). IR (KBr): ν_max_ 3402, 1664, 1620, 1082, 1033 cm^-1^. ^1^H-NMR: (500 MHz, C_5_D_5_N) see Table 3. ^13^C-NMR: (125 MHz, C_5_D_5_N) see Table 3. EI-MS (%): *m*/*z* 330 (*M*^+^, 35), 312 (19), 294 (13), 254 (17), 161 (24), 152 (76), 147 (29), 134 (100), 121 (34), 107 (18), 91 (14). HREI-MS: *m*/*z* 330.2183 (*M*^+^, [C_21_H_30_O_3_]^+^; calcd 330.2195). Crystal data: empirical formula = C_42_H_62_O_8_, *Mr* = 694.92, monoclinic, space group P2_1_, *a* = 7.7609 (5) Å, *b* = 13.2141 (8) Å, *c* = 18.6769 (11) Å, *V* = 1913.9 (2) Å^3^, Z = 2, *ρ*_calc_ = 1.206 mg m^-3^, F(000) = 756, *μ* (Mo K*α*) = 0.71073 Å, max/min transmission 0.9879/0.9751, crystal dimensions 0.31 × 0.17 × 0.15 mm, 1.09° < *θ* < 25.00°, 10,983 reflections were collected, out of which 3,534 reflections were observed (*R*_int_ = 0.0364) and 458 parameters were refined. The *R*-values were; *R*_1_ = 0.0523, *wR*_2_ = 0.1342 for I > 2*σ* (I), and *R*_1_ = 0.0699, *wR*_2_ = 0.1544 for all data, max/min residual electron density; 0.320/-0.311 e Å^-3^. Crystallographic data for compound **5** can be obtained from the Cambridge Crystallographic Data Center (code CCDC 799213) (Additional file 9 and Additional file 10).

### T-Cell proliferation inhibition assay

Peripheral blood mononuclear cells (PBMC) were isolated from heparinized venous blood of healthy adult donors by Ficoll–Hypaque gradient centrifugation [21]. Cells were proliferated as reported earlier [22]. Briefly, cells were cultured at a concentration of 2 × 10^6^/mL in a 96-well round bottom tissue culture plate. Cells were stimulated with 5 μg/mL of phytohemagglutinin. Various concentrations of compounds were added to obtain final concentrations of 0.5, 5, 50 μg/mL, each in triplicate. The plate was incubated for 72 h at 37°C in 5% CO_2_ environment. After 72 h, cells were pulsed with 0.5 μCi/well, tritiated thymidine, and further incubated for 18 h. Cells were harvested onto a glass fiber filter by using cell harvester. The tritiated thymidine incorporation into the cells, which reflects the proliferation level, was measured by a liquid scintillation counter.

### Phagocyte chemiluminescence assay

Luminol-enhanced chemiluminescence assay was performed according to the previous reported method [23]. Briefly 25 μL of whole blood or neutrophils (1 × 10^6^/mL), suspended in Hank’s solution, were incubated with 25 μL compounds (1, 10, 100 μg/mL for whole blood and 0.5, 5, 50 μg/mL for neutrophils) for 30 min. Zymosan 25 μL (20 mg/mL), followed by 25 μL (7 × 10^-5^ M) of luminol was added to make a final volume of 100 μL. A control without the compound was also run. Peak chemiluminescence was recorded using the luminometer. The luminometer was set with repeated scan mode, 50 scans with 30 s intervals and one second point measuring time.

### Cytotoxicity assay

The cytotoxicity of compounds was determined by using the MTT cellular assay [24,25] against a normal mouse fibroblast (3T3) cell line. Cells were grown in DMEM and MEM (modified Eagle’s medium), containing 10% FBS and 2% antibiotic (penicillin and streptomycin), and maintained at 37°C in 5% CO_2_ for 24 hours in a flask. Cells were plated (1 × 10^5^ cell/mL) in 96-well flat bottom plates and incubated for 24 hours for cell attachment. Various concentrations of compounds, ranging between 1.25-100 μM, were added into the well and incubated for 48 hours. A 50 μL [2 mg/mL] MTT, 3-(4,5-dimethylthiazol-2-yl)-2,5-diphenyltetrazolium bromide was added to the well, 4 hours before the end of incubation. Medium and reagents were aspirated and 100 μL DMSO was added and mixed thoroughly for 15 minutes to dissolve the formazan crystals. The absorbance was measured at 570 nm by using a microplate reader. Finally, IC_50_ (μM) values were calculated, and the experiment was repeated at least three times. Cycloheximide was used as the standard for normal fibroblast cell line.

## Competing interests

The authors have no competing interests.

## Authors’ contributions

MIC, AR, MAM and AMA-M participated in experimental strategy design, supervision and manuscript writing. NTK, MB and IHQ carried out the experiments. SY carried out the X-ray diffraction experiments. AW performed NMR experiments while MAM, ASK and SAS carried out the biological screenings. All authors read and approved the final manuscript.

## Supplementary Material

Additional file 1 **Spectroscopic data of compound 2**. Include spectra of ^1^H-NMR, EI-MS, HREI-MS, IR, and UV experiments. Click here for file

Additional file 2 Crystallographic information file (cif) of compound 2. Click here for file

Additional file 3 **Spectroscopic data of compound 3.** Include spectra of ^1^H-NMR, ^13^C-NMR (BB, DEPT-135), HSQC, HMBC, COSY-45°, NOESY, EI-MS, HREI-MS, and IR experiments. Click here for file

Additional file 4 **Spectroscopic data of compound 6.** Include spectra of ^1^H-NMR, ^13^C-NMR (BB, DEPT-135), HMQC, HMBC, COSY-45°, NOESY, and EI-MS. Click here for file

Additional file 5 Crystallographic information file (cif) of compound 6. Click here for file

Additional file 6 **Spectroscopic data of compound 7.** Include spectra of ^1^H-NMR, ^13^C-NMR (BB, DEPT-135), HMQC, HMBC, COSY-45°, NOESY, and EI-MS. Click here for file

Additional file 7 **Spectroscopic data of compound 4.** Include spectra of ^1^H-NMR, ^13^C-NMR (BB, DEPT-135), HSQC, HMBC, COSY-45°, NOESY, EI-MS, HREI-MS, IR and UV experiments. Click here for file

Additional file 8 Crystallographic information file (cif) of compound 4. Click here for file

Additional file 9 **Spectroscopic data of compound 5.** Include spectra of ^1^H-NMR, ^13^C-NMR (BB, DEPT-135), HSQC, HMBC, COSY-45°, NOESY, EI-MS, HREI-MS, and IR experiments. Click here for file

Additional file 10 Crystallographic information file (cif) of compound 5. Click here for file

## References

[B1] Fernandes P Cruz A Angelova B Pinheiro HM Cabral JMS Microbial conversion of steroid compounds: recent developments Enzyme Microb Technol 2003 32 688 705 10.1016/S0141-0229(03)00029-2

[B2] Charney W Herzog HL Microbial Transformations of Steroids 1967 Academic Press, New York 16 73

[B3] Choudhary MI Erum S Atif M Malik R Khan NT Atta-ur-Rahman Biotransformation of (20S)-20-hydroxymethylpregna-1,4-dien-3-one by four filamentous fungi Steroids 2011 76 1288 1296 21762714 10.1016/j.steroids.2011.06.007

[B4] Choudhary MI Shah SA Atta-ur-Rahman Khan SN Khan MTH Alpha-glucosidase and tyrosinase inhibitors from fungal hydroxylation of tibolone and hydroxytibolones Steroids 2010 75 956 966 10.1016/j.steroids.2010.05.017 20685216

[B5] Al-Aboudi A Mohammad MY Haddad S Al-Far R Choudhary MI Atta-ur-Rahman Biotransformation of methyl cholate by Aspergillus niger Steroids 2009 74 483 486 10.1016/j.steroids.2009.01.002 19428436

[B6] Azizuddin Choudhary MI Biotransformation of danazol by Fusarium solani and Gibberella fujikuorii, and prolylendopeptidase inhibition studies of transformed products Turk J Chem 2010 34 945 951

[B7] Choudhary MI Mohammad MY Musharraf SG Parvez M Al-Aboudi A Atta-ur-Rahman New oxandrolone derivatives by biotransformation using Rhizopus stolonifer Steroids 2009 74 1040 1044 10.1016/j.steroids.2009.08.003 19698730

[B8] Choudhary MI Khan NT Musharraf SG Anjum S Atta-ur-Rahman Biotransformation of adrenosterone by filamentous fungus. Cunninghamella elegans Steroids 2007 72 923 929 10.1016/j.steroids.2007.08.002 17889091

[B9] Hengge UR Stocks K Faulkner S Wiehler H Lorenz C Jentzen W Hengge D Ringham G Oxymetholone for the treatment of HIV-wasting: a double-blind, randomized, placebo-controlled phase III trial in eugonadal men and women HIV Clin Trials 2003 4 150 163 12815555 10.1310/hct.2003.4.3.002

[B10] Pavlatos AM Fultz O Monberg MJ Vootkur A Review of oxymetholone: a 17α-alkylated anabolic-androgenic steroid Clin Ther 2001 23 789 801 10.1016/S0149-2918(01)80070-9 11440282

[B11] Karrow NA McCay JA Brown R Musgrove D Munson AE White KL Jr Oxymetholone modulates cell-mediated immunity in male B6C3F1 mice Drug Chem Toxicol 2000 23 621 644 10.1081/DCT-100101974 11071398

[B12] Mukaida N Morita M Ishikawa Y Rice N Okamoto S Kasahara T Matsushima K Novel mechanism of glucocorticoid-mediated gene repression. Nuclear factor-kappa B is target for glucocorticoid-mediated interleukin 8 gene repression J Biol Chem 1994 269 13289 13295 8175759

[B13] Chuhjo T Yachie A Kanegane H Kimura H Shiobara S Nakao S Epstein-Barr virus (EBV)-associated post-transplantation lymphoproliferative disorder simultaneously affecting both B and T cells after allogeneic bone marrow transplantation Am J Hematol 2003 72 255 258 10.1002/ajh.10303 12666136

[B14] Filippin LI Vercelino R Marroni NP Xavier RM Redox signalling and the inflammatory response in rheumatoid arthritis Clin Exp Immunol 2008 152 415 422 10.1111/j.1365-2249.2008.03634.x 18422737 PMC2453196

[B15] Knox LH Velarde E Steroids. CCXIV.1 2α-Hydroxymethylandrostane derivatives J Org Chem 1962 27 3925 3929 10.1021/jo01058a042

[B16] Bond AM Dakternieks D Deprez PP Zuman P Polarographic and spectroscopic examination of the reaction of the anabolic steroid oxymetholone with methanol and ethanol J Org Chem 1988 53 1991 1996 10.1021/jo00244a027

[B17] Siemens SMART and SAINT 1996 Siemens Analytical X-Ray Instruments Inc, Madison

[B18] Altomare A Cascarano G Giacovazzo C Guagliardi A Completion and refinement of crystal structures with SIR92 J Appl Cryst 1993 26 343 350 10.1107/S0021889892010331

[B19] Sheldrick GM SHELXTL-PC (Version 5.1) 1997 Siemens Analytical Instruments, Inc, Madison

[B20] Jhnson CK ‘ORTEPII’, Report ORNL-5138 1976 Oak Ridge National Laboratory, Tennessee

[B21] Böyum A Isolation of mononuclear cells and granulocytes from human blood. Isolation of monuclear cells by one centrifugation and of granulocytes by combining centrifugation and sedimentation at 1 g Scand J Clin Lab Invest 1968 97 77 89 4179068

[B22] Nielsen M Gerwien J Geisler C Röpke C Svejgaard A Odum N MHC class II ligation induces CD58 (LFA-3)-mediated adhesion in human T cells Exp Clin Immunogenet 1998 15 61 68 10.1159/000019055 9691200

[B23] Helfand SL Werkmeister J Roder JC Chemiluminescence response of human natural killer cells. I. The relationship between target cell binding, chemiluminescence, and cytolysis J Exp Med 1982 156 492 505 10.1084/jem.156.2.492 6178787 PMC2186753

[B24] Mosmann T Rapid colorimetric assay for cellular growth and survival: application to proliferation and cytotoxicity assays J Immunol Methods 1983 65 55 63 10.1016/0022-1759(83)90303-4 6606682

[B25] Choudhary MI Ismail M Shaari K Abbaskhan A Sattar SA Lajis NH Atta-ur-Rahman Cis-Clerodane-type furanoditerpenoids from Tinospora crispa J Nat Prod 2010 73 541 547 10.1021/np900551u 20356064

