# Supplementary material for: Synthesis of some potent immunomodulatory and anti-inflammatory metabolites by fungal transformation of anabolic steroid oxymetholone
Source: Chem Cent J. 2012 Dec 10;6:153. doi: 10.1186/1752-153X-6-153 (PMC3740782; doi:10.1186/1752-153X-6-153)
Supplement: Additional file 1 — Spectroscopic data of compound 2. Include spectra of 1H-NMR, EI-MS, HREI-MS, IR, and UV experiments. [file 1752-153X-6-153-S1.pdf]

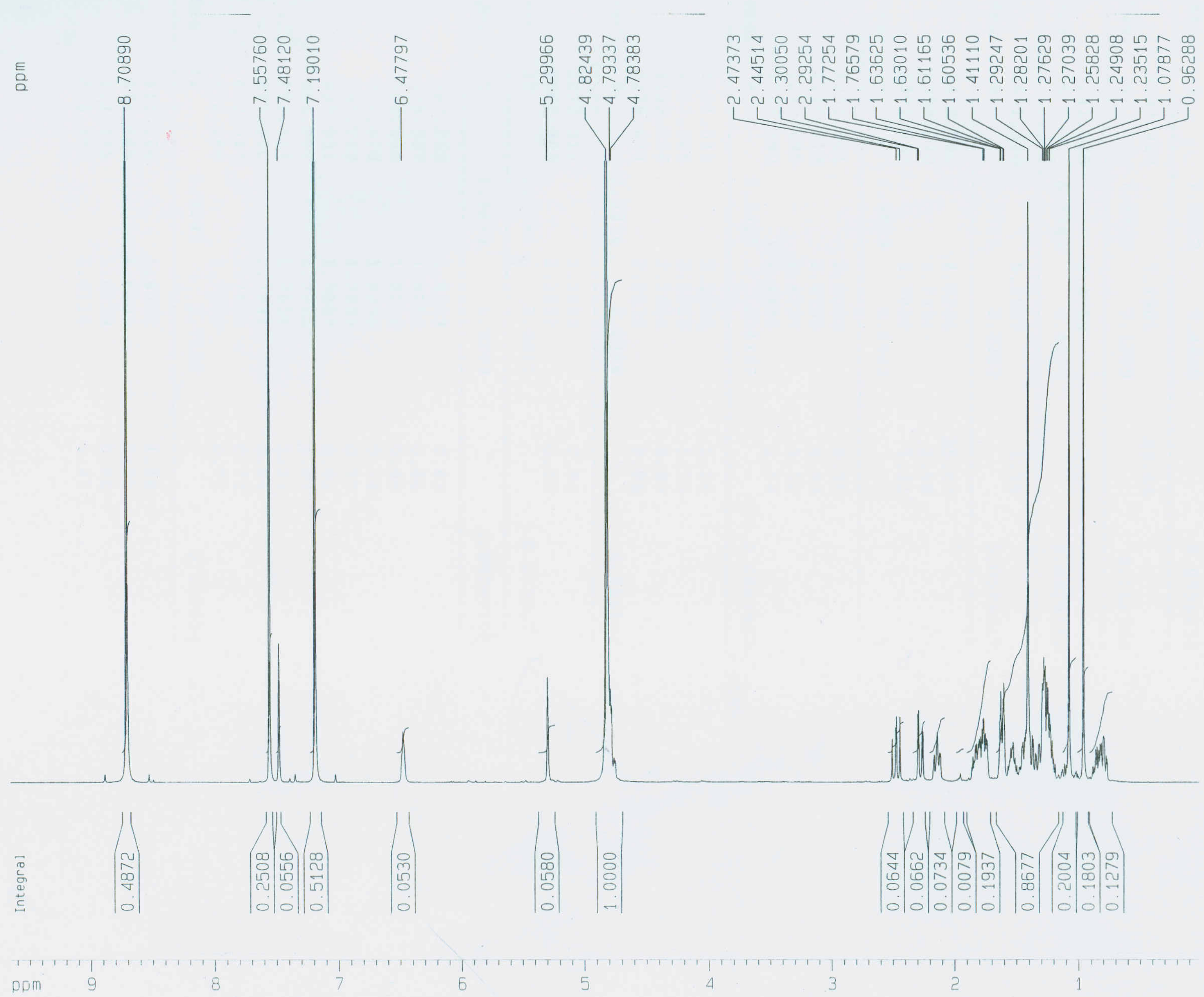

Current Data Parameters
NAME oct29
EXPNO 3
PROCNO 1

F2 - Acquisition Parameters
Date_ 20081029
Time 11.39
INSTRUM spect
PROBHD 5 mm BBI 1H-BB
PULPROG zg30
TD 32768
SOLVENT Pyr
NS 128
DS 0
SWH 10000.000 Hz
FIDRES 0.305176 Hz
AQ 1.6385000 sec
RG 406.4
DW 50.000 usec
DE 6.00 usec
TE 305.4 K
D1 1.00000000 sec
MCREST 0.00000000 sec
MCWRK 0.01500000 sec

======== CHANNEL f1 ========
NUC1 1H
P1 7.00 usec
PL1 0.00 dB
SFO1 500.3340026 MHz

F2 - Processing parameters
SI 16384
SF 500.3307526 MHz
WDW EM
SSB 0
LB 0.30 Hz
GB 0
PC 1.20

1D NMR plot parameters
CX 20.00 cm
CY 25.00 cm
F1P 9.655 ppm
F1 4830.81 Hz
F2P 0.020 ppm
F2 10.18 Hz
PPMCM 0.48174 ppm/cm
HZCM 241.03139 Hz/cm

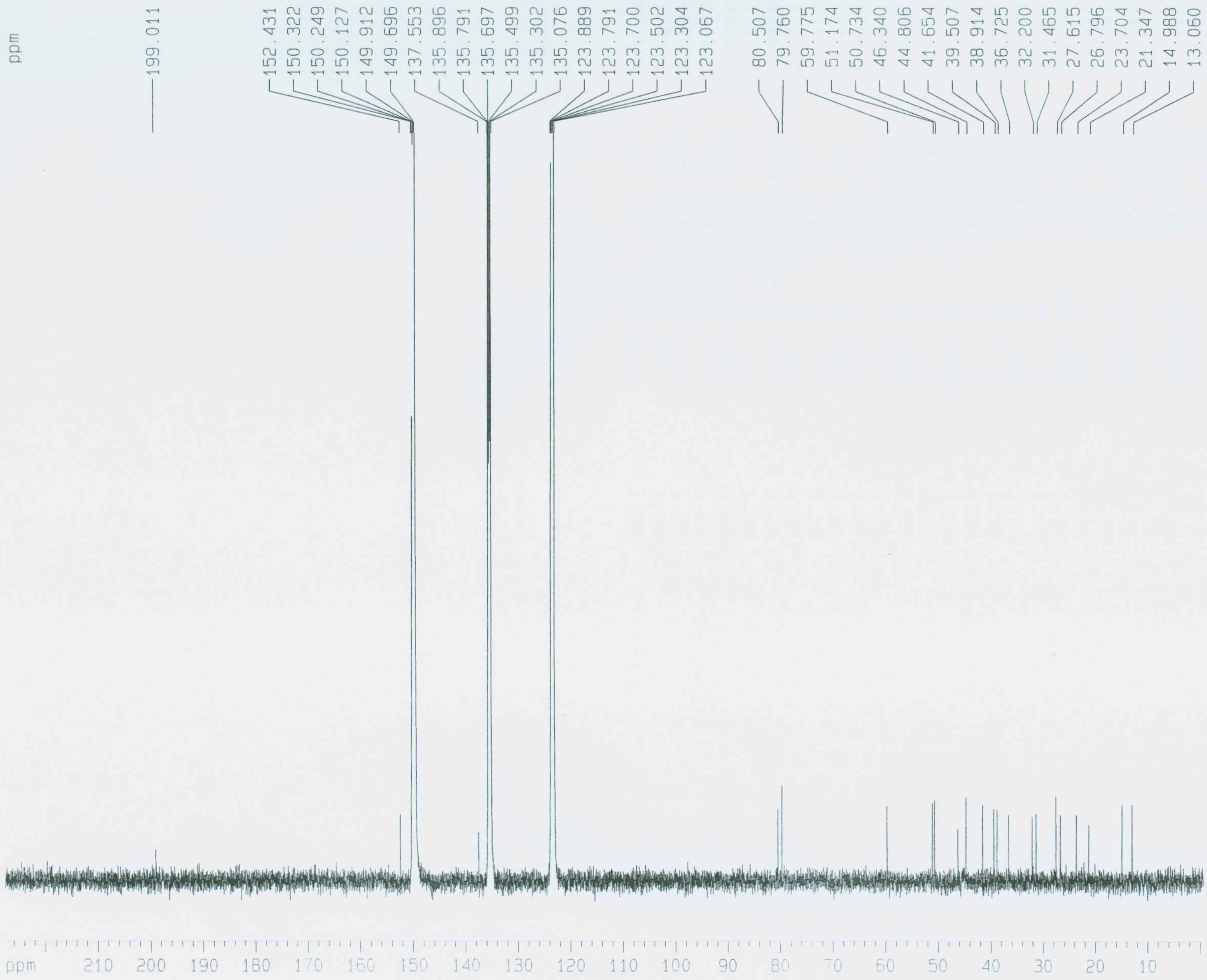

Current Data Parameters

NAME	oct29
EXPNO	8
PROCNO	1

F2 - Acquisition Parameters

Date_	20081030
Time	5.47
INSTRUM	spect
PROBHD	5 mm BBI 1H-BB
PULPROG	zgpg30
TD	65536
SOLVENT	Pyr
NS	20480
DS	4
SWH	30030.029 Hz
FIDRES	0.458222 Hz
AQ	1.0912410 sec
RG	32768
DW	16.650 usec
DE	6.00 usec
TE	304.8 K
D1	1.50000000 sec
d11	0.03000000 sec
DELTA	1.39999998 sec
MCREST	0.00000000 sec
MCWRK	0.01500000 sec

======== CHANNEL f1 ========

NUC1	13C
P1	13.50 usec
PL1	-2.00 dB
SFO1	125.8221695 MHz

======== CHANNEL f2 ========

CPDPRG2	waltz16
NUC2	1H
PCPD2	100.00 usec
PL2	0.00 dB
PL12	24.00 dB
PL13	24.00 dB
SFO2	500.3325016 MHz

F2 - Processing parameters

SI	32768
SF	125.8082360 MHz
WDW	EM
SSB	0
LB	1.50 Hz
GB	0
PC	1.20

1D NMR plot parameters

CX	20.00 cm
CY	250.00 cm
F1P	227.692 ppm
F1	28645.56 Hz
F2P	-0.568 ppm
F2	-71.50 Hz
PPMCM	11.41303 ppm/cm
HZCM	1435.85291 Hz/cm

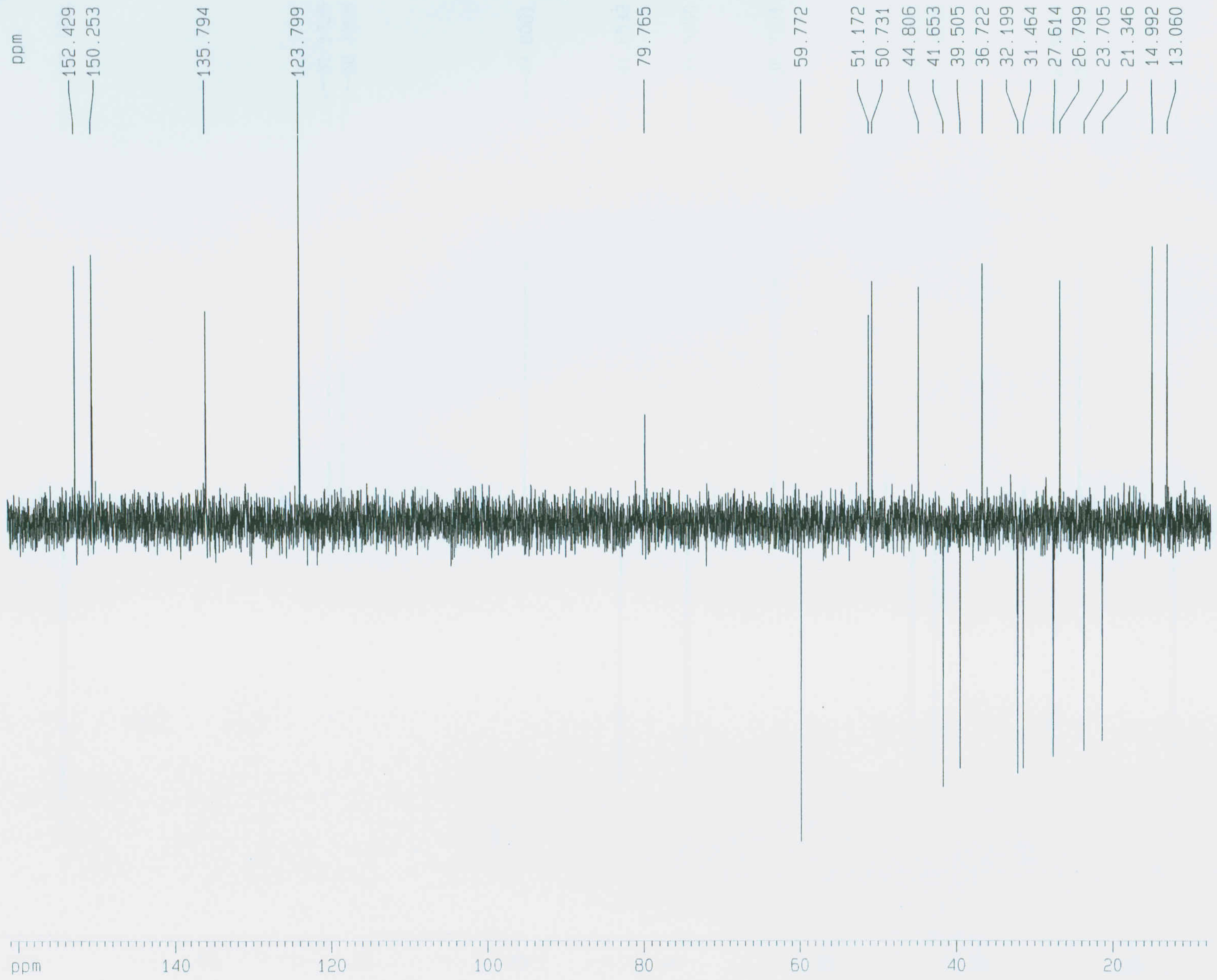

Current Data Parameters
NAME oct29
EXPNO 9
PROCNO 1

F2 - Acquisition Parameters
Date_ 20081030
Time 20.53
INSTRUM spect
PROBHD 5 mm BBI 1H-BB
PULPROG deptsp135
TD 65536
SOLVENT Pyr
NS 10240
DS 4
SWH 30030.029 Hz
FIDRES 0.458222 Hz
AQ 1.0912410 sec
RG 32768
DW 16.650 usec
DE 6.00 usec
TE 305.4 K
CNST2 145.0000000
D1 1.50000000 sec
d2 0.00344828 sec
d12 0.00002000 sec
DELTA 0.00001719 sec
MCREST 0.00000000 sec
MCWRK 0.01500000 sec

======== CHANNEL f1 ========
NUC1 13C
P1 13.50 usec
P12 2000.00 usec
PL0 120.00 dB
PL1 -2.00 dB
SFO1 125.8206594 MHz
SP2 1.99 dB
SPNAM2 Crp60comp.4
SPOFF2 0.00 Hz

======== CHANNEL f2 ========
CPDPRG2 waltz16
NUC2 1H
P3 7.00 usec
p4 14.00 usec
PCPD2 100.00 usec
PL2 0.00 dB
PL12 24.00 dB
SFO2 500.3330020 MHz

F2 - Processing parameters
SI 32768
SF 125.8082360 MHz
WDW EM
SSB 0
LB 1.50 Hz
GB 0
PC 1.20

1D NMR plot parameters
CX 20.00 cm
CY 6.50 cm
F1P 161.061 ppm
F1 20262.74 Hz
F2P 7.460 ppm
F2 938.48 Hz
PPMCM 7.68005 ppm/cm
HZCM 966.21295 Hz/cm

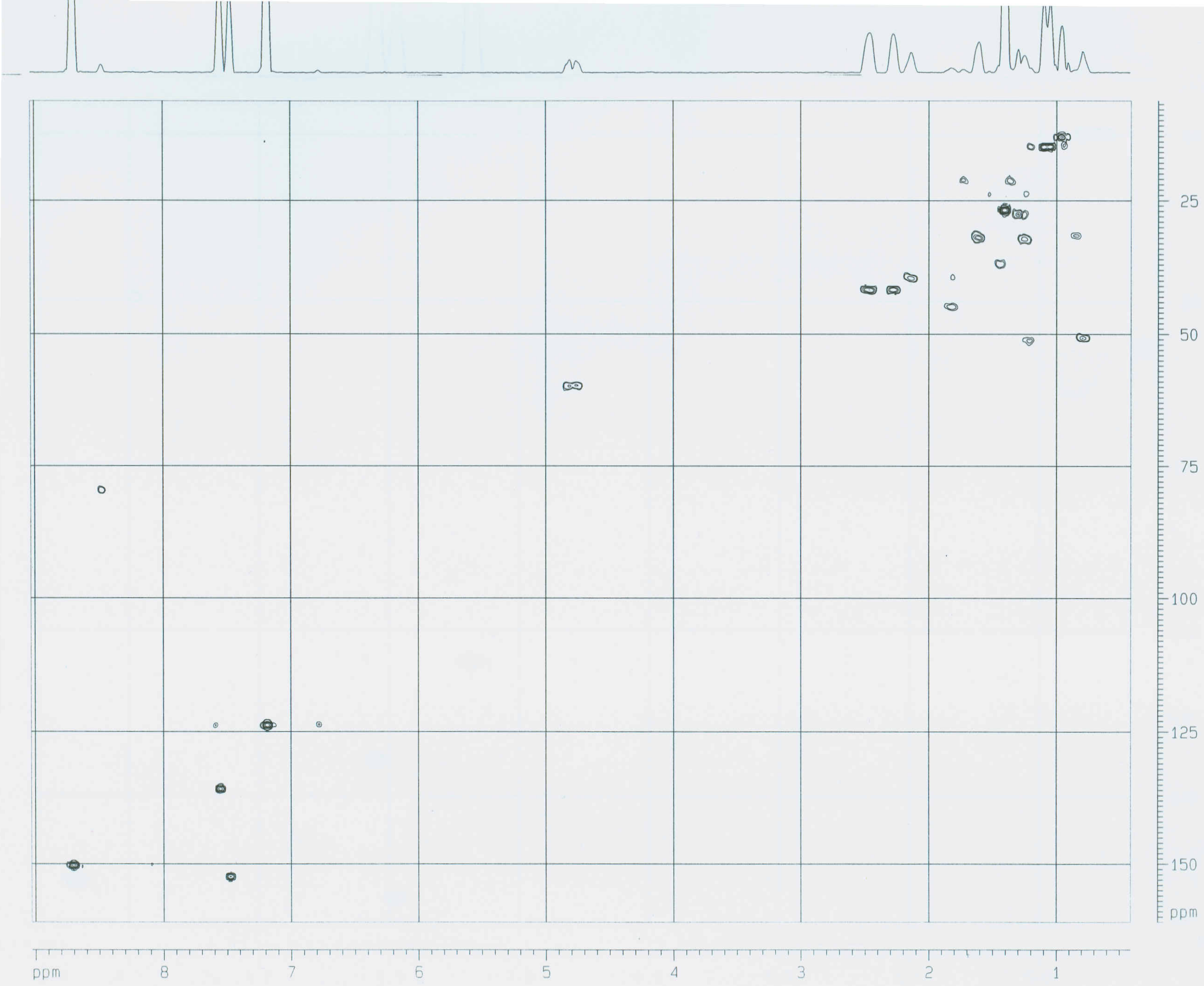

Current Data Parameters

NAME	oct29
EXPNO	6
PROCNO	1

F2 - Acquisition Parameters

Date_	20081029
Time	16.04
INSTRUM	spect
PROBHD	5 mm BBI 1H-BB
PULPROG	hmqcgpqf
TD	1024
SOLVENT	Pyr
NS	32
DS	16
SWH	4496.403 Hz
FIDRES	4.391018 Hz
AQ	0.1140300 sec
RG	26008
DW	111.200 usec
DE	6.00 usec
TE	305.9 K
CNST2	145.0000000
d0	0.00000300 sec
D1	1.50000000 sec
d2	0.00344828 sec
d12	0.00002000 sec
d13	0.00000400 sec
D16	0.00010000 sec
DELTA1	0.00232428 sec
IN0	0.00002208 sec
MCREST	0.00000000 sec
MCWRK	1.50000000 sec

======== CHANNEL f1 ========

NUC1	1H
P1	7.00 usec
p2	14.00 usec
PL1	0.00 dB
SFO1	500.3331020 MHz

======== CHANNEL f2 ========

CPDPRG2	garp
NUC2	13C
P3	13.50 usec
PCPD2	78.00 usec
PL2	-2.00 dB
PL12	13.00 dB
SFO2	125.8194017 MHz

====== GRADIENT CHANNEL =====

GPNAM1	SINE.100
GPNAM2	SINE.100
GPNAM3	SINE.100
GPX1	0.00 %
GPX2	0.00 %
GPX3	0.00 %
GPY1	0.00 %
GPY2	0.00 %
GPY3	0.00 %
GPZ1	50.00 %
GPZ2	30.00 %
GPZ3	40.10 %
P16	1000.00 usec

F1 - Acquisition parameters

ND0	2
TD	256
SFO1	125.8194 MHz
FIDRES	88.476784 Hz
SW	180.020 ppm
FnMODE	QF

F2 - Processing parameters

SI	1024
SF	500.3307525 MHz
WDW	QSINE
SSB	0
LB	0.00 Hz
GB	0
PC	1.40

F1 - Processing parameters

SI	1024
MC2	QF
SF	125.8082360 MHz
WDW	QSINE
SSB	0
LB	0.00 Hz
GB	0

2D NMR plot parameters

CX2	20.00 cm
CX1	15.00 cm
F2PLO	9.031 ppm
F2LO	4518.66 Hz
F2PHI	0.413 ppm
F2HI	206.68 Hz
F1PLO	161.013 ppm
F1LO	20256.75 Hz
F1PHI	6.294 ppm
F1HI	791.86 Hz
F2PPMCM	0.43091 ppm/cm
F2HZCM	215.59901 Hz/cm
F1PPMCM	10.31458 ppm/cm
F1HZCM	1297.65955 Hz/cm

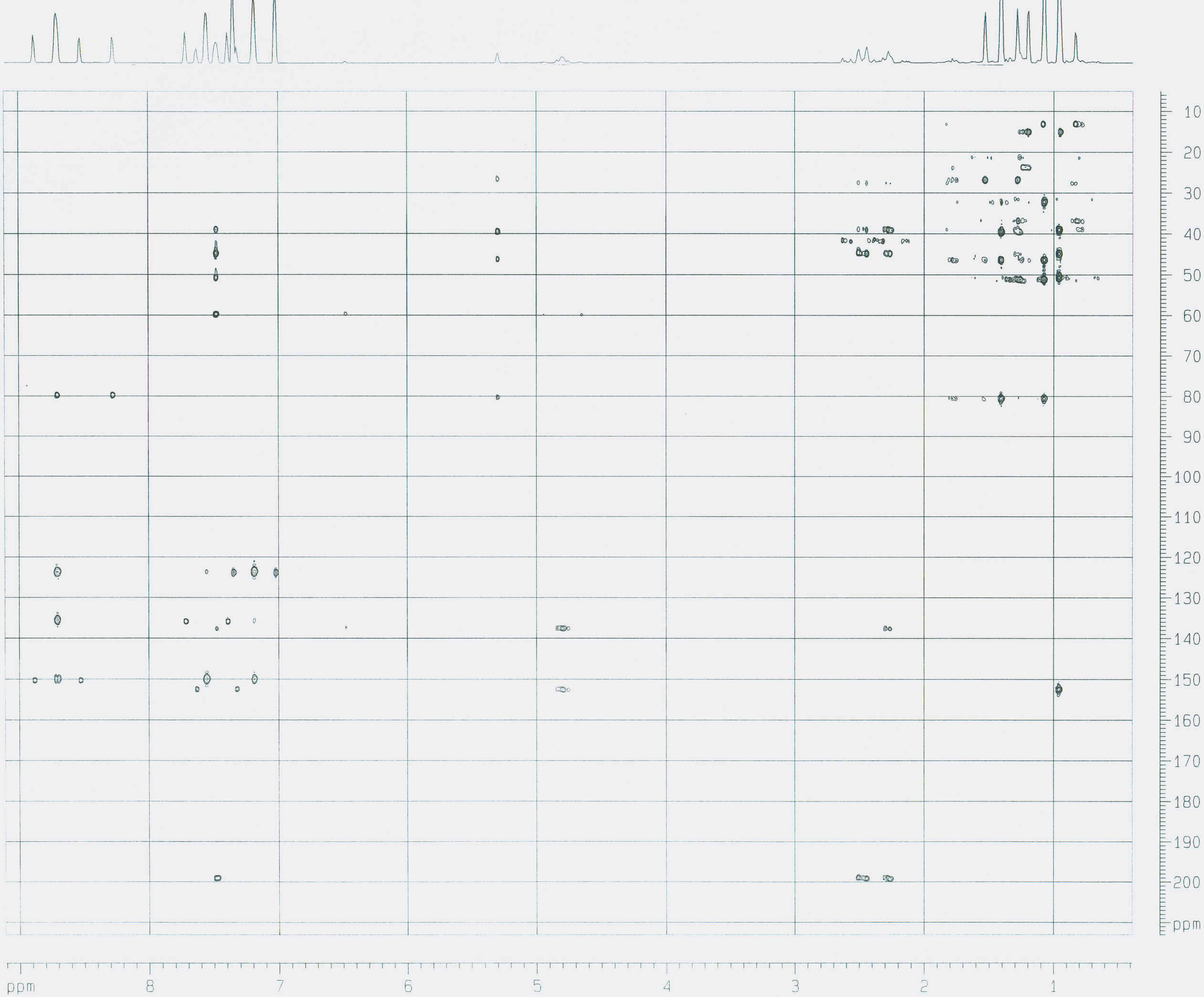

Current Data Parameters

NAME	oct29
EXPNO	7
PROCNO	1

F2 - Acquisition Parameters

Date_	20081029
Time	19.49
INSTRUM	spect
PROBHD	5 mm BBI 1H-BB
PULPROG	hmbcgpndqf
TD	4096
SOLVENT	Pyr
NS	64
DS	16
SWH	4496.403 Hz
FIDRES	1.097755 Hz
AQ	0.4556364 sec
RG	20642.5
DW	111.200 usec
DE	6.00 usec
TE	305.7 K
CNST13	8.0000000
d0	0.00000300 sec
D1	1.50000000 sec
d6	0.06250000 sec
D16	0.00010000 sec
IN0	0.00001728 sec
MCREST	0.00000000 sec
MCWRK	1.50000000 sec

======== CHANNEL f1 ========

NUC1	1H
P1	7.00 usec
p2	14.00 usec
PL1	0.00 dB
SFO1	500.3331020 MHz

======== CHANNEL f2 ========

NUC2	13C
P3	13.50 usec
PL2	-2.00 dB
SFO2	125.8225469 MHz

====== GRADIENT CHANNEL =====

GPNAM1	SINE.100
GPNAM2	SINE.100
GPNAM3	SINE.100
GPX1	0.00 %
GPX2	0.00 %
GPX3	0.00 %
GPY1	0.00 %
GPY2	0.00 %
GPY3	0.00 %
GPZ1	50.00 %
GPZ2	30.00 %
GPZ3	40.10 %
P16	1000.00 usec

F1 - Acquisition parameters

ND0	2
TD	256
SFO1	125.8225 MHz
FIDRES	113.060783 Hz
SW	230.035 ppm
FnMODE	QF

F2 - Processing parameters

SI	1024
SF	500.3307525 MHz
WDW	QSINE
SSB	8
LB	0.00 Hz
GB	0
PC	1.40

F1 - Processing parameters

SI	1024
MC2	QF
SF	125.8082360 MHz
WDW	QSINE
SSB	8
LB	0.00 Hz
GB	0

2D NMR plot parameters

CX2	20.00 cm
CX1	15.00 cm
F2PLO	9.110 ppm
F2LO	4558.18 Hz
F2PHI	0.387 ppm
F2HI	193.51 Hz
F1PLO	213.056 ppm
F1LO	26804.18 Hz
F1PHI	5.013 ppm
F1HI	630.61 Hz
F2PPMCM	0.43618 ppm/cm
F2HZCM	218.23361 Hz/cm
F1PPMCM	13.86956 ppm/cm
F1HZCM	1744.90466 Hz/cm

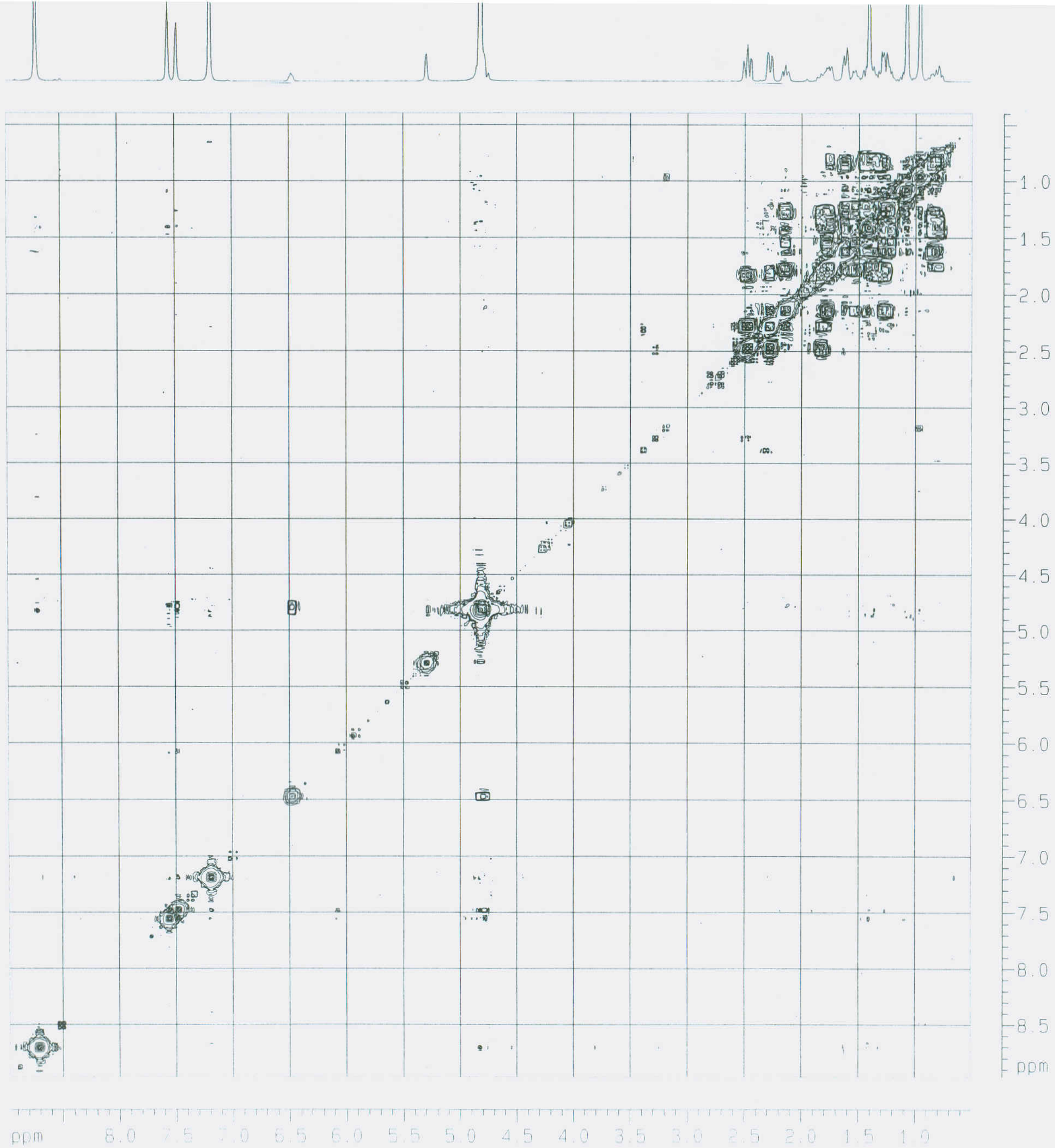

Current Data Parameters

NAME	oct29
EXPNO	4
PROCNO	1

F2 - Acquisition Parameters

Date_	20081029
Time	12.09
INSTRUM	spect
PROBHD	5 mm BBI 1H-BB
PULPROG	cosygf45
TD	2048
SOLVENT	Pyr
NS	16
DS	16
SWH	4496.403 Hz
FIDRES	2.195509 Hz
AQ	0.2278988 sec
RG	256
DW	111.200 usec
DE	6.00 usec
TE	305.5 K
d0	0.00000300 sec
D1	1.50000000 sec
IN0	0.00022240 sec
MCREST	0.00000000 sec
MCWRK	1.50000000 sec

======== CHANNEL f1 ========

NUC1	1H
P1	7.00 usec
PL1	0.00 dB
SFO1	500.3331020 MHz

F1 - Acquisition parameters

ND0	1
TD	128
SFO1	500.3331 MHz
FIDRES	35.128147 Hz
SW	8.987 ppm
FnMODE	QF

F2 - Processing parameters

SI	1024
SF	500.3307525 MHz
WDW	SINE
SSB	0
LB	0.00 Hz
GB	0
PC	1.40

F1 - Processing parameters

SI	1024
MC2	QF
SF	500.3307525 MHz
WDW	SINE
SSB	0
LB	0.00 Hz
GB	0

2D NMR plot parameters

CX2	15.00 cm
CX1	15.00 cm
F2PLO	8.970 ppm
F2LO	4487.92 Hz
F2PHI	0.510 ppm
F2HI	254.98 Hz
F1PLO	8.970 ppm
F1LO	4487.92 Hz
F1PHI	0.396 ppm
F1HI	197.90 Hz
F2PPMCM	0.56402 ppm/cm
F2HZCM	282.19611 Hz/cm
F1PPMCM	0.57163 ppm/cm
F1HZCM	286.00168 Hz/cm

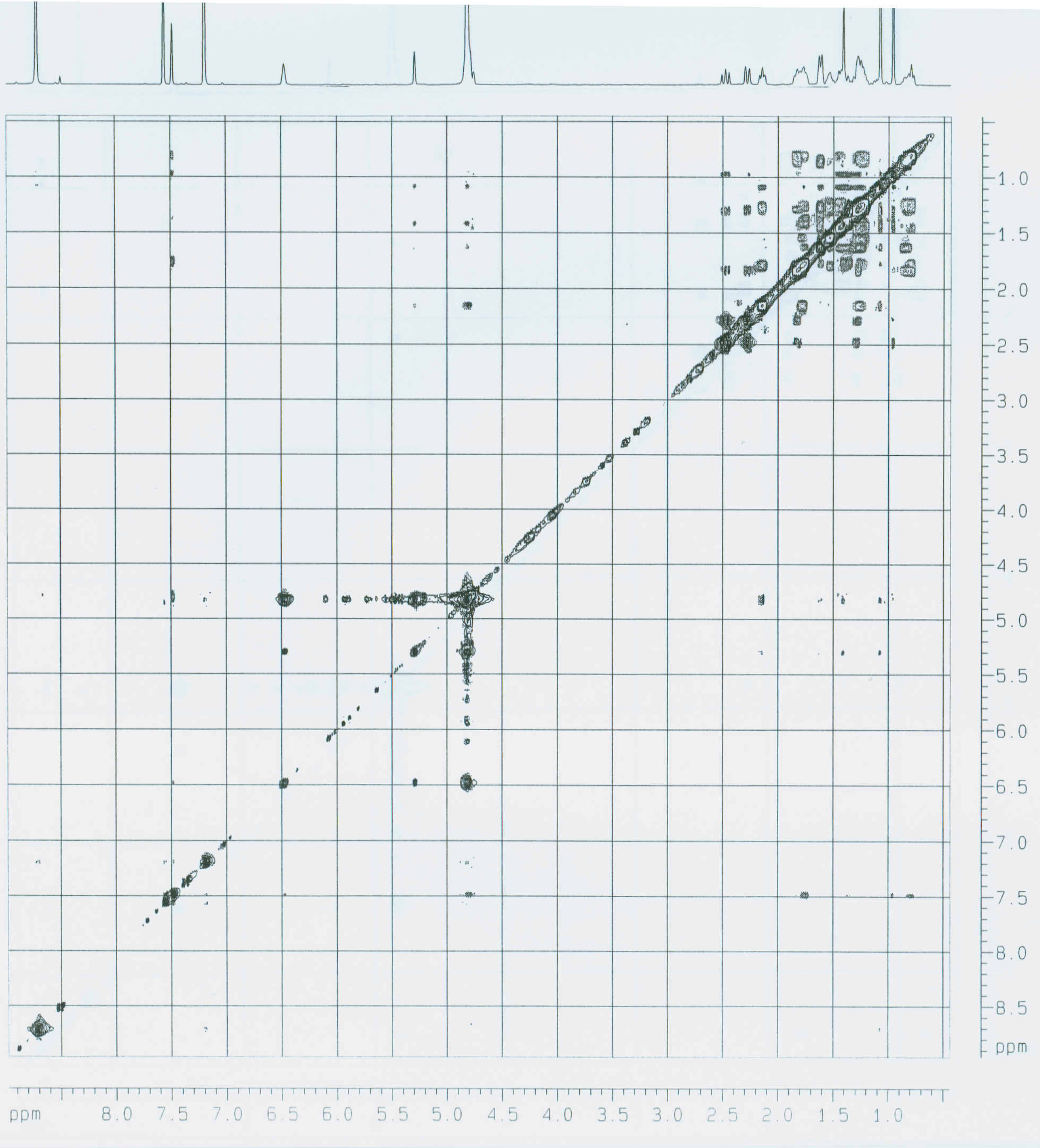

Current Data Parameters

NAME	oct29
EXPNO	5
PROCNO	1

F2 - Acquisition Parameters

Date_	20081029
Time	13.09
INSTRUM	spect
PROBHD	5 mm BBI 1H-BB
PULPROG	noesyph
TD	2048
SOLVENT	Pyr
NS	16
DS	16
SWH	4496.403 Hz
FIDRES	2.195509 Hz
AQ	0.2278988 sec
RG	406.4
DW	111.200 usec
DE	6.00 usec
TE	305.5 K
d0	0.00010229 sec
D1	1.50000000 sec
D8	0.80000001 sec
IN0	0.00022240 sec
MCREST	0.00000000 sec
MCWRK	0.75000000 sec
ST1CNT	128

======== CHANNEL f1 ========

NUC1	1H
P1	7.00 usec
PL1	0.00 dB
SFO1	500.3331020 MHz

F1 - Acquisition parameters

ND0	1
TD	256
SFO1	500.3331 MHz
FIDRES	17.564074 Hz
SW	8.987 ppm
FnMODE	States-TPPI

F2 - Processing parameters

SI	2048
SF	500.3307525 MHz
WDW	QSINE
SSB	2
LB	0.00 Hz
GB	0
PC	1.00

F1 - Processing parameters

SI	512
MC2	States-TPPI
SF	500.3307525 MHz
WDW	QSINE
SSB	2
LB	0.00 Hz
GB	0

2D NMR plot parameters

CX2	15.00 cm
CX1	15.00 cm
F2PLO	8.979 ppm
F2LO	4492.31 Hz
F2PHI	0.435 ppm
F2HI	217.66 Hz
F1PLO	8.961 ppm
F1LO	4483.53 Hz
F1PHI	0.448 ppm
F1HI	224.24 Hz
F2PPMCM	0.56958 ppm/cm
F2HZCM	284.97708 Hz/cm
F1PPMCM	0.56753 ppm/cm
F1HZCM	283.95255 Hz/cm

File Name	:	d:\mswin\data\ox22-hp.mss
Creation Date/Time	:	09/02/09 at 9:50:16
File Type	:	Lo-Res Mass Data (Centroid)
File Source	:	Acquired on MASPEC system [msw/A091]
File Title	:	EI
Operator	:	Barkat Ali
Instrument	:	MAT312

SCAN GRAPH. Flagging=M/z. Filter=[Int:1%.].

Scan 14-4:37. Entries=159. 100% Int.=18403.

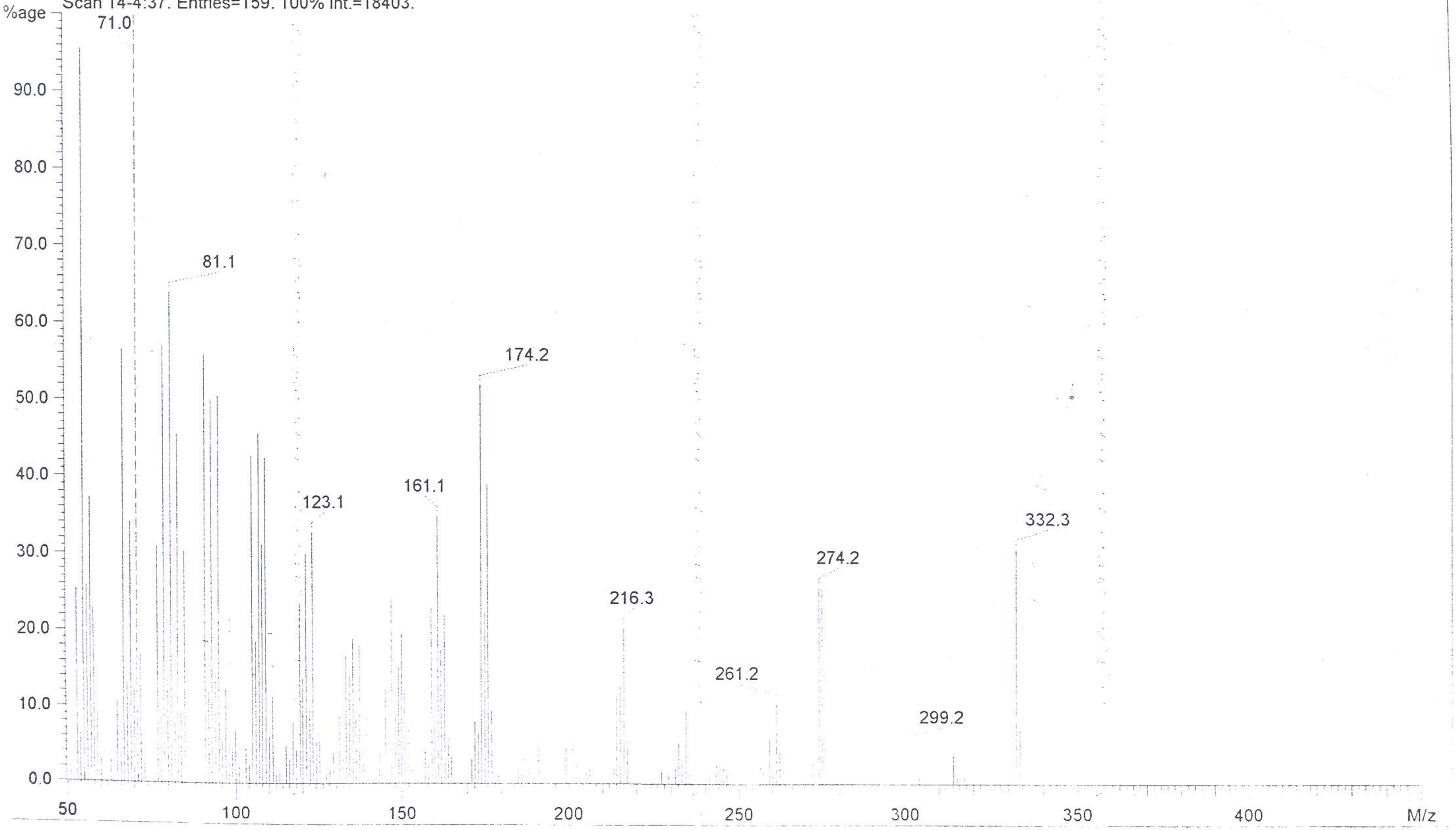

Mass	Relative Intensity	Theoretical Mass	Delta [ppm]	Delta [mmu]	RDB	Composition
275.2003	17.6544	275.2011	-3.1	-0.8	5.5	$C_{18} H_{27} O_{2}$
276.2003	3.3175					
280.9819	9.7604	280.9781	13.5	3.8	5.5	$C_{7} H_{5} O_{12}$
281.1896	2.9451	281.1905	-3.3	-0.9	8.5	$C_{20} H_{25} O_{1}$
284.2129	3.1433	284.2140	-3.9	-1.1	7.0	$C_{20} H_{28} O_{1}$
286.2268	1.4836	286.2297	-10.0	-2.9	6.0	$C_{20} H_{30} O_{1}$
292.9819	9.5245	292.9781	12.9	3.8	6.5	$C_{8} H_{5} O_{12}$
296.2113	1.5328	296.2140	-9.1	-2.7	8.0	$C_{21} H_{28} O_{1}$
297.1846	7.0706	297.1855	-3.0	-0.9	8.5	$C_{20} H_{25} O_{2}$
298.1893	1.9096	298.1933	-13.2	-3.9	8.0	$C_{20} H_{26} O_{2}$
299.2011	28.4630	299.2011	-0.2	-0.1	7.5	$C_{20} H_{27} O_{2}$
300.2045	5.1009	300.2089	-14.9	-4.5	7.0	$C_{20} H_{28} O_{2}$
301.2086	1.4197					
302.2235	3.2691	302.2246	-3.6	-1.1	6.0	$C_{20} H_{30} O_{2}$
304.2426	1.9147	304.2402	7.8	2.4	5.0	$C_{20} H_{32} O_{2}$
304.9819	2.6261	304.9781	12.4	3.8	7.5	$C_{9} H_{5} O_{12}$
312.2072	2.5911	312.2089	-5.6	-1.8	8.0	$C_{21} H_{28} O_{2}$
314.2245	9.1012	314.2246	-0.1	-0.0	7.0	$C_{21} H_{30} O_{2}$
315.2270	2.0671	315.2324	-17.1	-5.4	6.5	$C_{21} H_{31} O_{2}$
316.9819	1.4628	316.9781	12.1	3.8	8.5	$C_{10} H_{5} O_{12}$
330.2205	2.2666	330.2195	3.0	1.0	7.0	$C_{21} H_{30} O_{3}$
330.9787	6.2506	330.9785	0.6	0.2	4.5	$C_{7} H_{7} O_{15}$
332.2333	11.9683	332.2351	-5.5	-1.8	6.0	$C_{21} H_{32} O_{3}$
333.2394	3.3526	333.2430	-10.7	-3.6	5.5	$C_{21} H_{33} O_{3}$
342.9813	3.9285	342.9785	8.2	2.8	5.5	$C_{8} H_{7} O_{15}$
354.9787	1.7820	354.9785	0.6	0.2	6.5	$C_{9} H_{7} O_{15}$
380.9755	3.6226	380.9730	6.5	2.5	12.5	$C_{14} H_{5} O_{13}$
		380.9789	-8.9	-3.4	3.5	$C_{7} H_{9} O_{18}$
392.9755	3.3694	392.9730	6.3	2.5	13.5	$C_{15} H_{5} O_{13}$
		392.9789	-8.6	-3.4	4.5	$C_{8} H_{9} O_{18}$
404.9755	2.1185	404.9730	6.1	2.5	14.5	$C_{16} H_{5} O_{13}$
		404.9789	-8.4	-3.4	5.5	$C_{9} H_{9} O_{18}$
430.9723	3.0147	430.9734	-2.6	-1.1	11.5	$C_{14} H_{7} O_{16}$
442.9723	1.8247	442.9734	-2.5	-1.1	12.5	$C_{15} H_{7} O_{16}$
454.9723	2.1510	454.9734	-2.4	-1.1	13.5	$C_{16} H_{7} O_{16}$

H. E. J. R. I. C., University of Karachi Analytical Laboratory - Pakistan

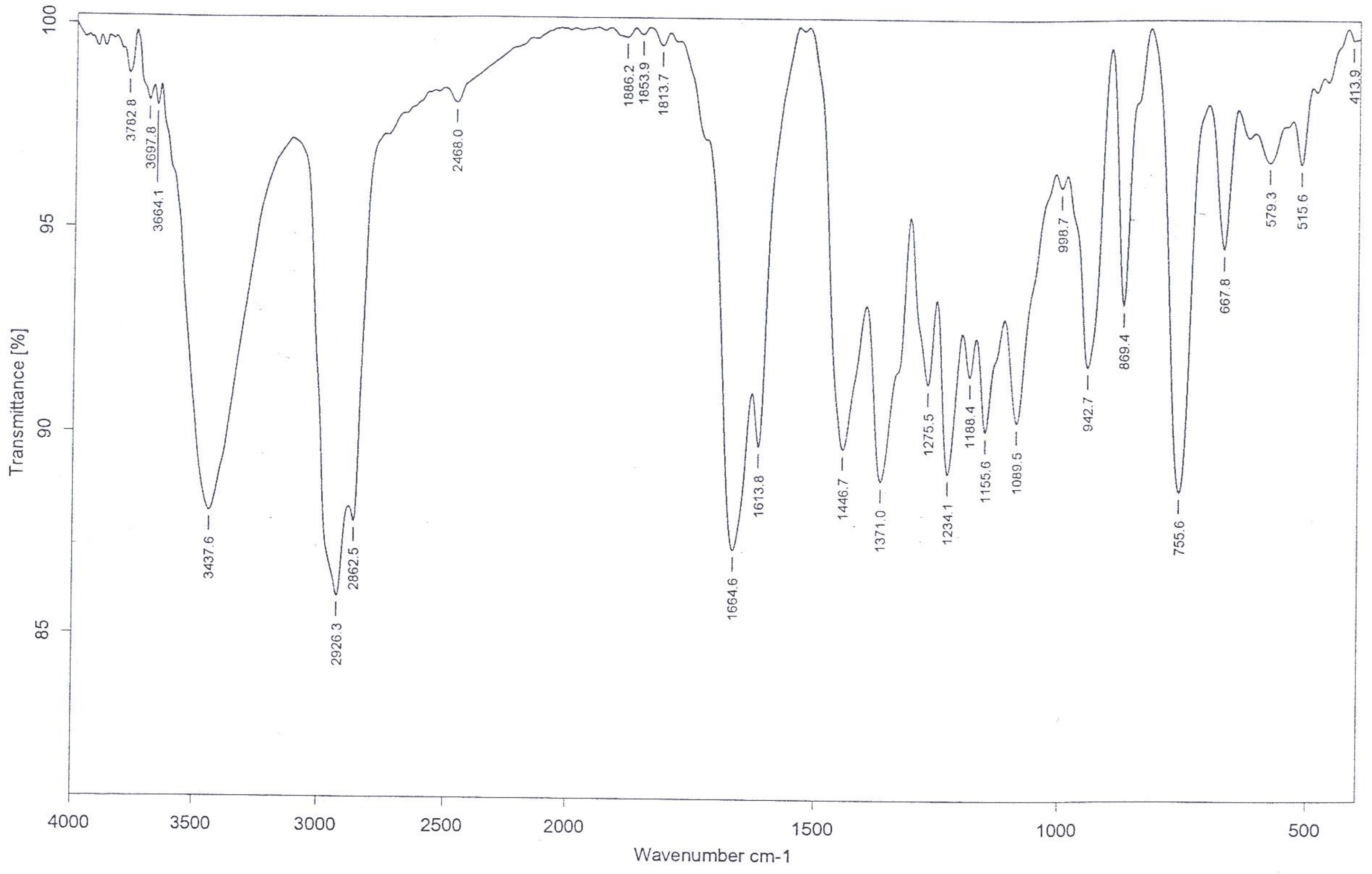

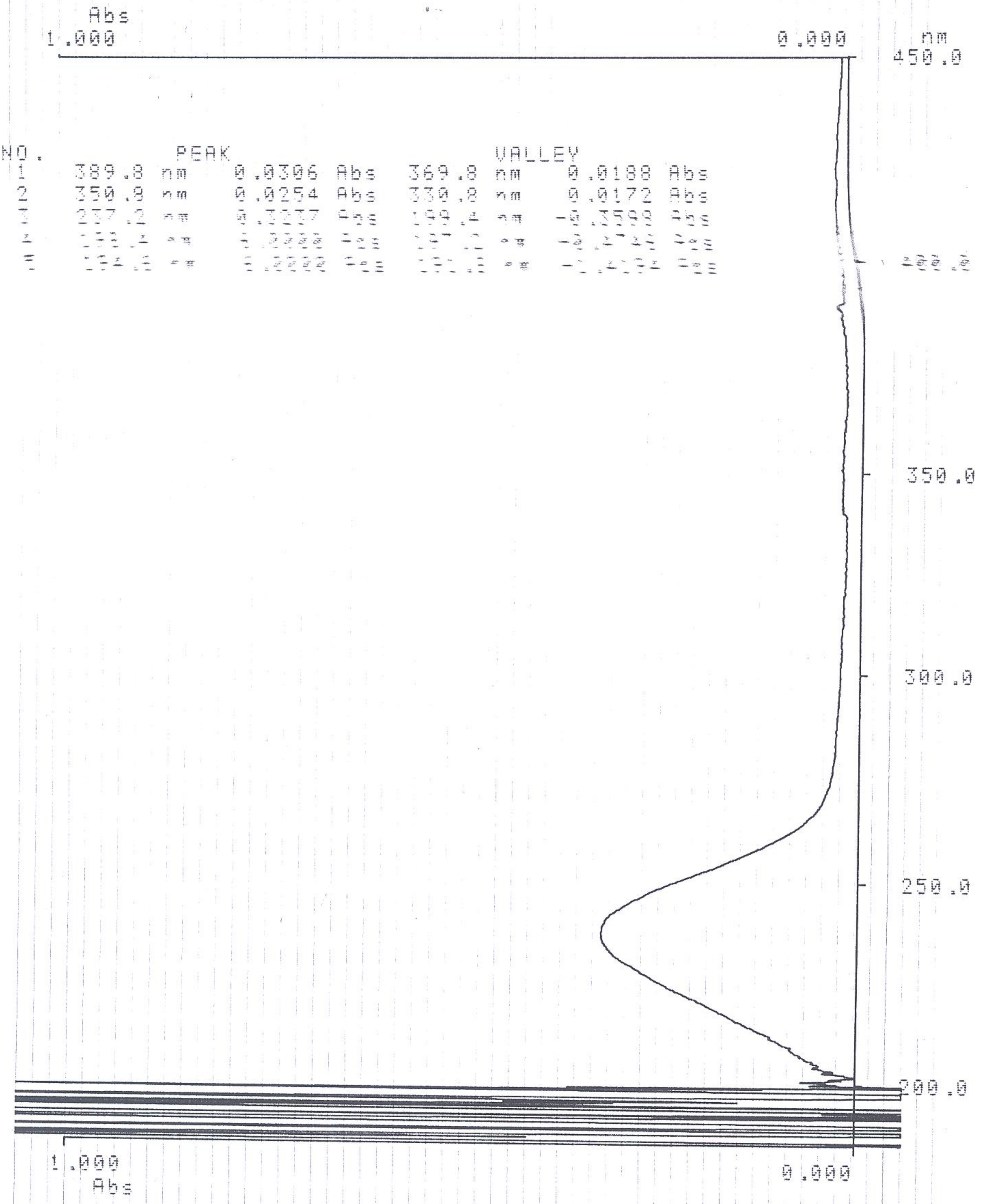

NO.	PEAK		VALLEY	
1	389.8 nm	0.0306 Abs	369.8 nm	0.0188 Abs
2	350.8 nm	0.0254 Abs	330.8 nm	0.0172 Abs
3	237.2 nm	0.3237 Abs	199.4 nm	-0.3599 Abs
[illegible]	[illegible]	[illegible]	[illegible]	[illegible]
[illegible]	[illegible]	[illegible]	[illegible]	[illegible]

TITLE: NAIK/OX-22HP
SCAN SPEED: 120.0 nm/min
BANDPASS: 2.00nm

12:43 PM 1/26/ 9
RESPONSE: MEDIUM

HITACHI
