# Supplementary material for: Synthesis of some potent immunomodulatory and anti-inflammatory metabolites by fungal transformation of anabolic steroid oxymetholone
Source: Chem Cent J. 2012 Dec 10;6:153. doi: 10.1186/1752-153X-6-153 (PMC3740782; doi:10.1186/1752-153X-6-153)
Supplement: Additional file 2 — Crystallographic information file (cif) of compound 2. [file 1752-153X-6-153-S2.pdf]

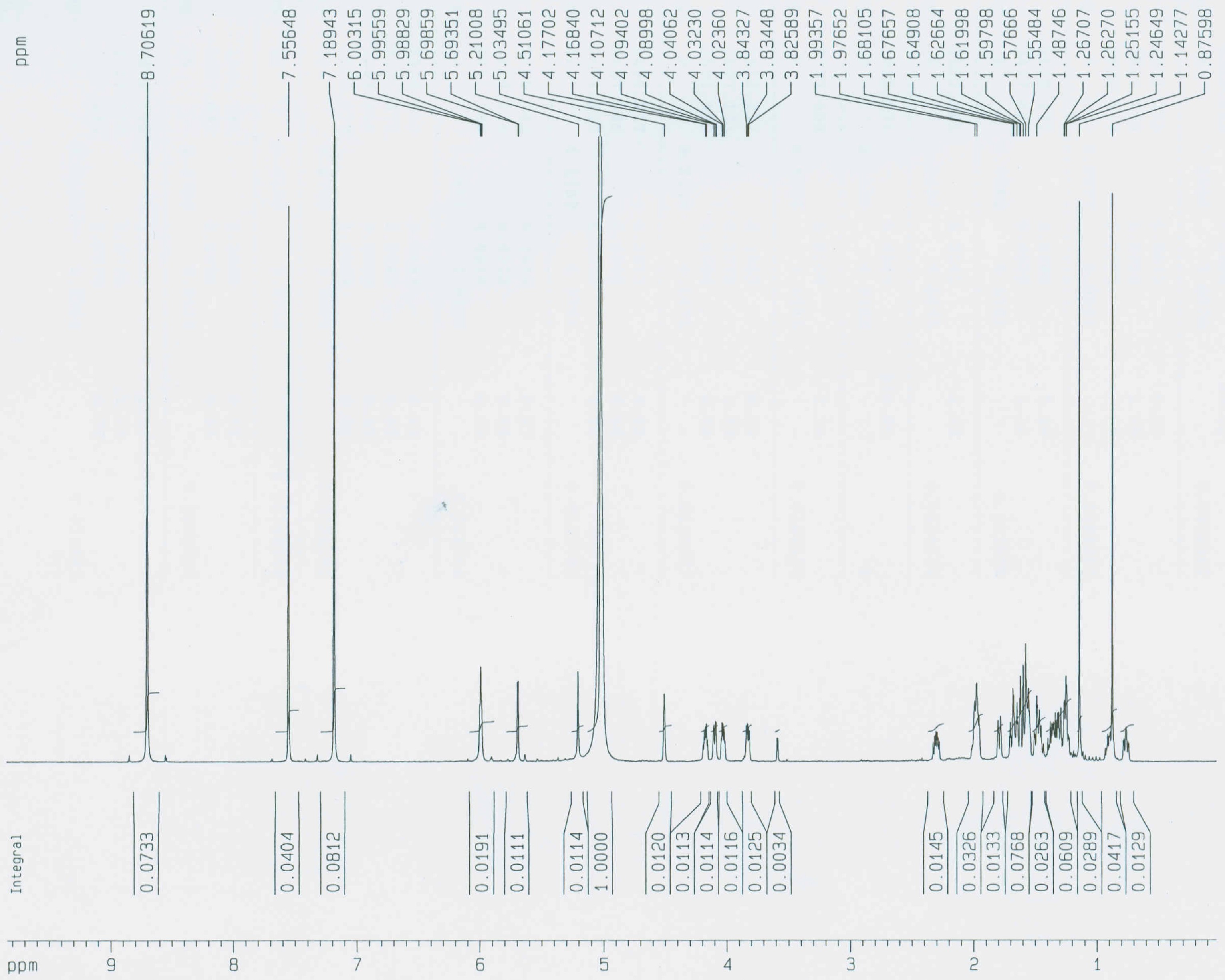

Current Data Parameters	
NAME	August21
EXPNO	1
PROCNO	1

F2 - Acquisition Parameters	
Date_	20080821
Time	10.16
INSTRUM	spect
PROBHD	5 mm CPTCI 1H-
PULPROG	zg30
TD	32768
SOLVENT	Pyr
NS	16
DS	0
SWH	12376.237 Hz
FIDRES	0.377693 Hz
AQ	1.3239176 sec
RG	10.1
DW	40.400 usec
DE	6.00 usec
TE	298.6 K
D1	1.00000000 sec
MCREST	0.00000000 sec
MCWRK	0.01500000 sec

======== CHANNEL f1 ========	
NUC1	1H
P1	7.40 usec
PL1	3.30 dB
SFO1	600.2337067 MHz

F2 - Processing parameters	
SI	16384
SF	600.2306842 MHz
WDW	EM
SSB	0
LB	0.30 Hz
GB	0
PC	1.20

1D NMR plot parameters	
CX	20.00 cm
CY	220.00 cm
F1P	9.845 ppm
F1	5909.35 Hz
F2P	0.029 ppm
F2	17.16 Hz
PPMCM	0.49083 ppm/cm
HZCM	294.60974 Hz/cm

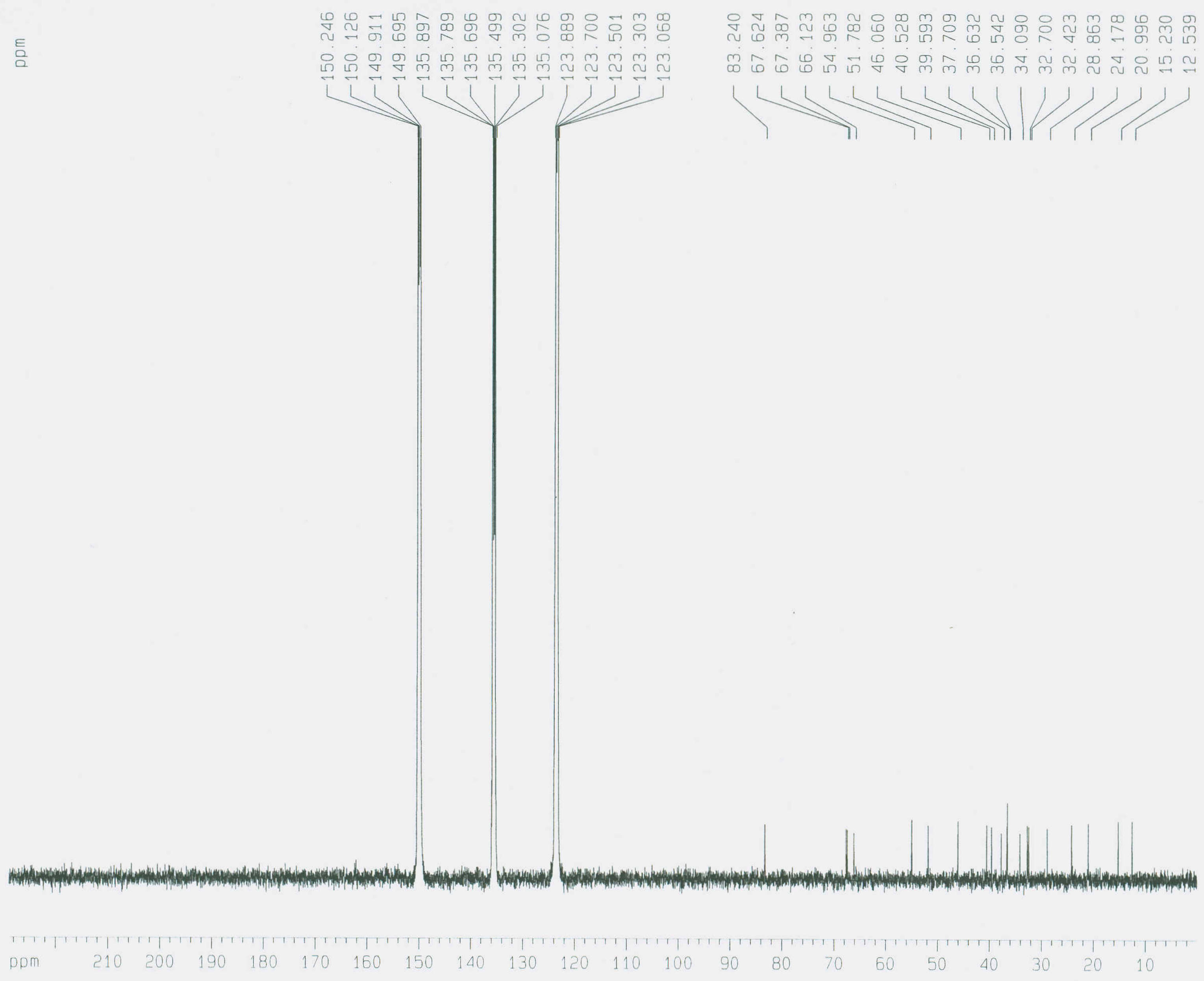

Current Data Parameters	
NAME	nov15
EXPNO	7
PROCNO	1
F2 - Acquisition Parameters	
Date_	20081115
Time	12.28
INSTRUM	spect
PROBHD	5 mm BBI 1H-BB
PULPROG	zgpg30
TD	65536
SOLVENT	Pyr
NS	20480
DS	4
SWH	30030.029 Hz
FIDRES	0.458222 Hz
AQ	1.0912410 sec
RG	32768
DW	16.650 usec
DE	6.00 usec
TE	304.5 K
D1	1.50000000 sec
d11	0.03000000 sec
DELTA	1.39999998 sec
MCREST	0.00000000 sec
MCWRK	0.01500000 sec
======== CHANNEL f1 ========	
NUC1	13C
P1	13.50 usec
PL1	-2.00 dB
SFO1	125.8221695 MHz
======== CHANNEL f2 ========	
CPDPRG2	waltz16
NUC2	1H
PCPD2	100.00 usec
PL2	0.00 dB
PL12	24.00 dB
PL13	24.00 dB
SFO2	500.3325016 MHz
F2 - Processing parameters	
SI	32768
SF	125.8081911 MHz
WDW	EM
SSB	0
LB	1.50 Hz
GB	0
PC	1.20
1D NMR plot parameters	
CX	20.00 cm
CY	200.00 cm
F1P	228.852 ppm
F1	28791.46 Hz
F2P	0.056 ppm
F2	7.07 Hz
PPMCM	11.43979 ppm/cm
HZCM	1439.21936 Hz/cm

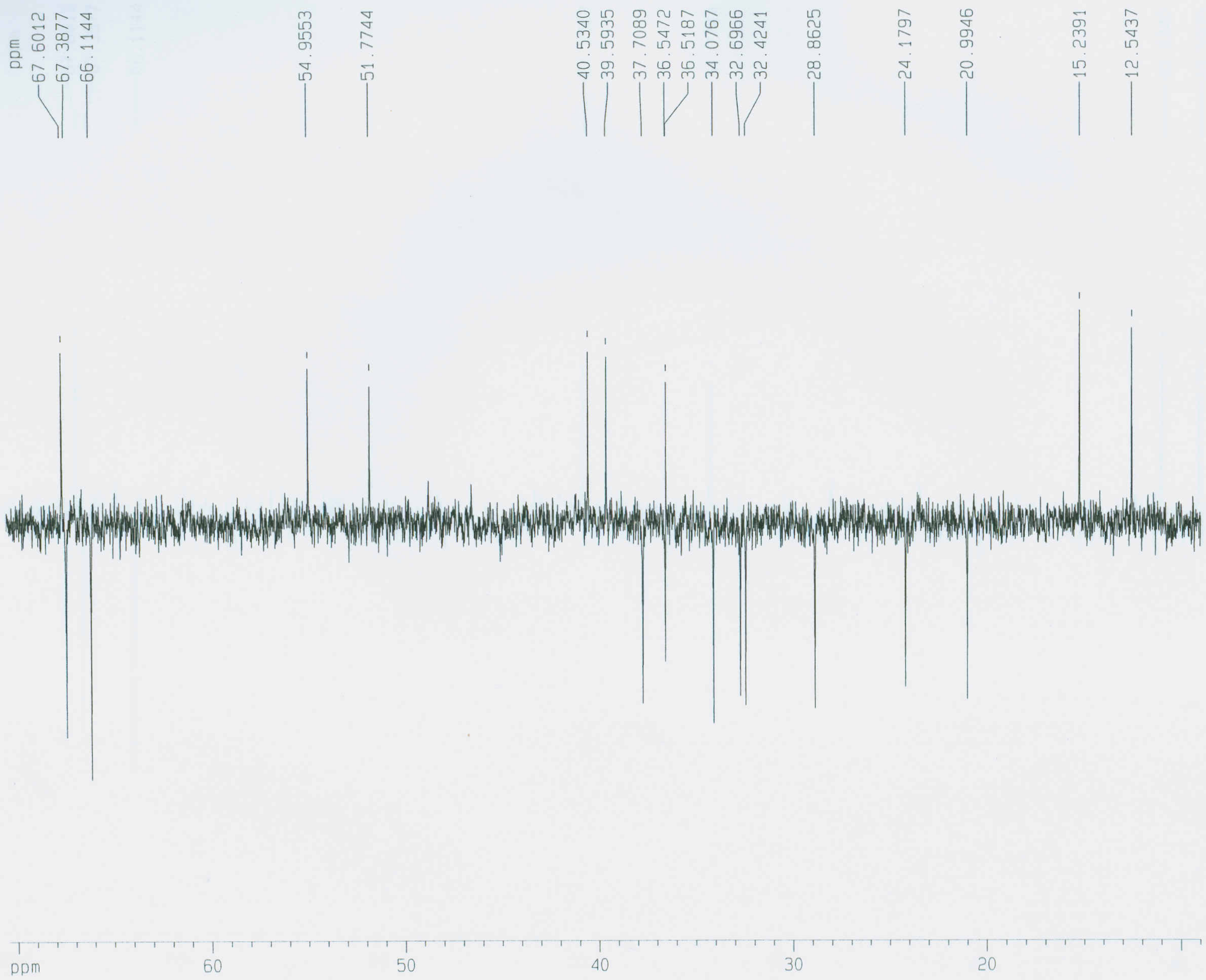

Current Data Parameters

NAME	nov15
EXPNO	8
PROCNO	1

F2 - Acquisition Parameters

Date_	20081116
Time	3.59
INSTRUM	spect
PROBHD	5 mm BBI 1H-BB
PULPROG	deptsp135
TD	65536
SOLVENT	Pyr
NS	9216
DS	4
SWH	30030.029 Hz
FIDRES	0.458222 Hz
AQ	1.0912410 sec
RG	32768
DW	16.650 usec
DE	6.00 usec
TE	302.6 K
CNST2	145.0000000
D1	1.50000000 sec
d2	0.00344828 sec
d12	0.00002000 sec
DELTA	0.00001719 sec
MCREST	0.00000000 sec
MCWRK	0.01500000 sec

======== CHANNEL f1 ========

NUC1	13C
P1	13.50 usec
P12	2000.00 usec
PL0	120.00 dB
PL1	-2.00 dB
SFO1	125.8206594 MHz
SP2	1.99 dB
SPNAM2	Crp60comp.4
SPOFF2	0.00 Hz

======== CHANNEL f2 ========

CPDPRG2	waltz16
NUC2	1H
P3	7.00 usec
p4	14.00 usec
PCPD2	100.00 usec
PL2	0.00 dB
PL12	24.00 dB
SFO2	500.3330020 MHz

F2 - Processing parameters

SI	32768
SF	125.8081911 MHz
WDW	EM
SSB	0
LB	1.50 Hz
GB	0
PC	1.20

1D NMR plot parameters

CX	20.00 cm
CY	5.00 cm
F1P	70.478 ppm
F1	8866.73 Hz
F2P	8.985 ppm
F2	1130.36 Hz
PPMCM	3.07467 ppm/cm
HZCM	386.81876 Hz/cm

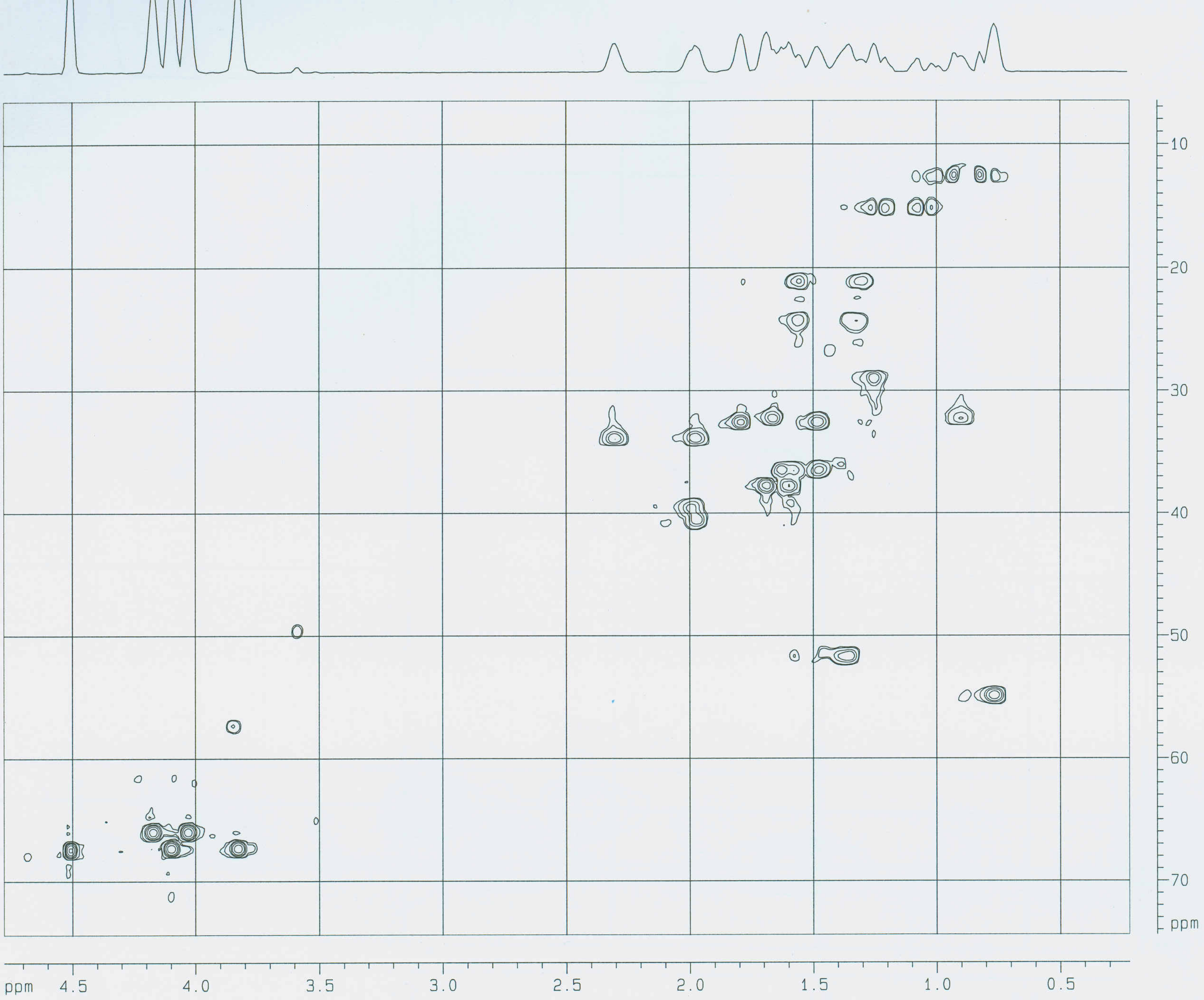

Current Data Parameters

NAME	August21
EXPNO	4
PROCNO	1

F2 - Acquisition Parameters

Date_	20080821
Time	13.10
INSTRUM	spect
PROBHD	5 mm CPTCI 1H-
PULPROG	hsqcetgp
TD	1024
SOLVENT	Pyr
NS	32
DS	16
SWH	6009.615 Hz
FIDRES	5.868765 Hz
AQ	0.0853300 sec
RG	46341
DW	83.200 usec
DE	6.00 usec
TE	298.8 K
CNST2	145.0000000
d0	0.00000300 sec
D1	1.50000000 sec
d4	0.00172414 sec
d11	0.03000000 sec
d13	0.00000400 sec
D16	0.00015000 sec
DELTA	0.00217080 sec
DELTA1	50000000.00000000 sec
IN0	0.00001744 sec
MCREST	0.00000000 sec
MCWRK	0.30000001 sec
ST1CNT	128

======== CHANNEL f1 ========

NUC1	1H
P1	7.40 usec
p2	14.80 usec
P28	0.50 usec
PL1	3.30 dB
SFO1	600.2337214 MHz

======== CHANNEL f2 ========

CPDPRG2	garp
NUC2	13C
P3	16.00 usec
p4	32.00 usec
PCPD2	60.00 usec
PL2	2.00 dB
PL12	17.56 dB
SFO2	150.9422922 MHz

====== GRADIENT CHANNEL =====

GPNAM1	SINE.100
GPNAM2	SINE.100
GPX1	0.00 %
GPX2	0.00 %
GPY1	0.00 %
GPY2	0.00 %
GPZ1	80.00 %
GPZ2	20.10 %
P16	2000.00 usec

F1 - Acquisition parameters

ND0	2
TD	256
SFO1	150.9423 MHz
FIDRES	112.007172 Hz
SW	189.966 ppm
FnMODE	Echo-Antiecho

F2 - Processing parameters

SI	1024
SF	600.2306842 MHz
WDW	QSINE
SSB	2
LB	0.00 Hz
GB	0
PC	1.40

F1 - Processing parameters

SI	1024
MC2	echo-antiecho
SF	150.9280949 MHz
WDW	QSINE
SSB	2
LB	0.00 Hz
GB	0

2D NMR plot parameters

CX2	20.00 cm
CX1	15.00 cm
F2PLO	4.777 ppm
F2LO	2867.00 Hz
F2PHI	0.220 ppm
F2HI	132.16 Hz
F1PLO	74.400 ppm
F1LO	11229.07 Hz
F1PHI	6.496 ppm
F1HI	980.42 Hz
F2PPMCM	0.22782 ppm/cm
F2HZCM	136.74223 Hz/cm
F1PPMCM	4.52695 ppm/cm
F1HZCM	683.24377 Hz/cm

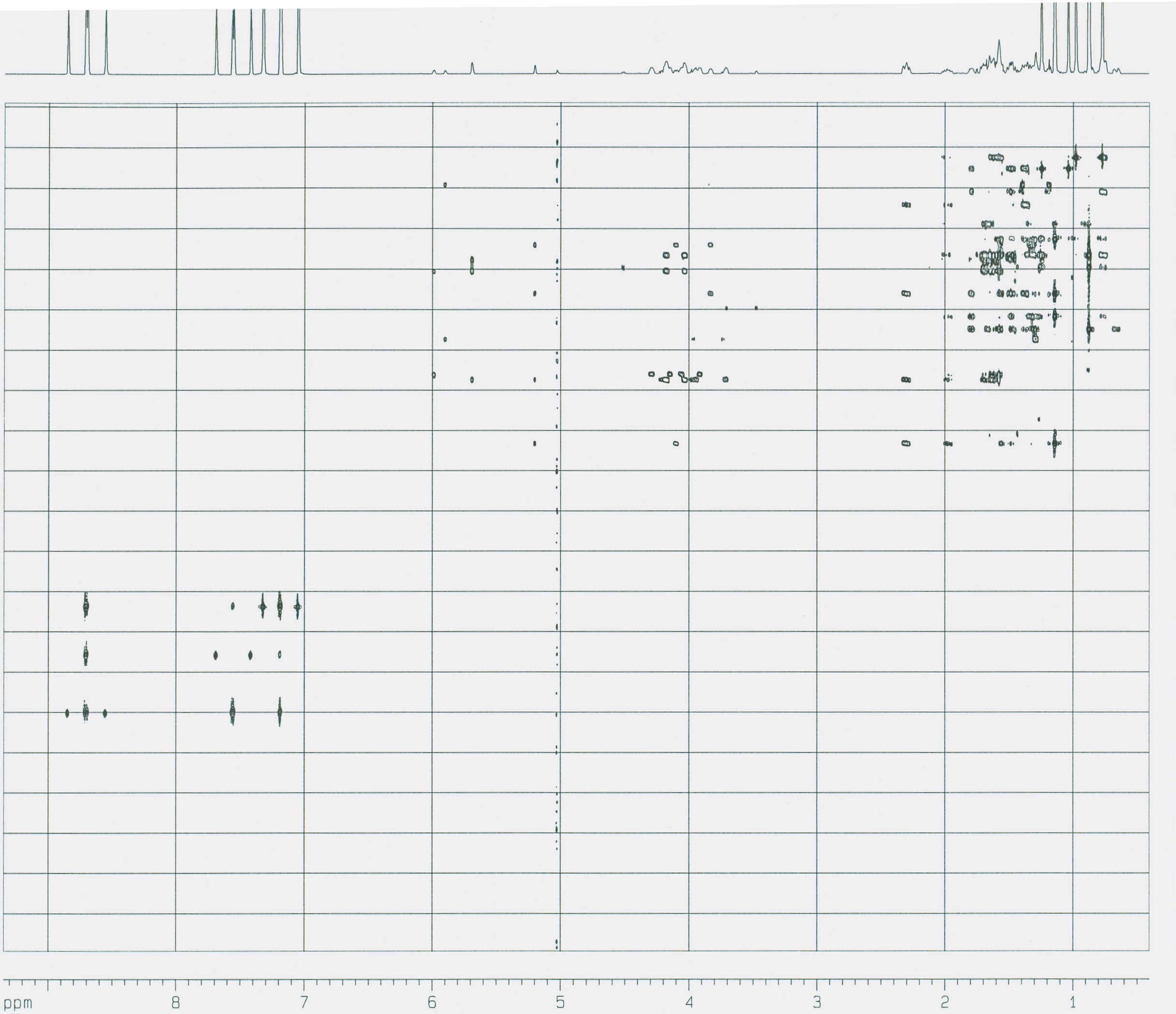

Current Data Parameters

NAME	August21
EXPNO	5
PROCNO	1

F2 - Acquisition Parameters

Date_	20080821
Time	16.50
INSTRUM	spect
PROBHD	5 mm CPTCI 1H-
PULPROG	hmbcgpndqf
TD	4096
SOLVENT	Pyr
NS	32
DS	16
SWH	6009.615 Hz
FIDRES	1.467191 Hz
AQ	0.3409204 sec
RG	32768
DW	83.200 usec
DE	6.00 usec
TE	299.4 K
CNST13	8.0000000
d0	0.00000300 sec
D1	1.50000000 sec
d6	0.06250000 sec
D16	0.00015000 sec
IN0	0.00001427 sec
MCREST	0.00000000 sec
MCWRK	1.50000000 sec

======== CHANNEL f1 ========

NUC1	1H
P1	7.40 usec
p2	14.80 usec
PL1	3.30 dB
SFO1	600.2337214 MHz

======== CHANNEL f2 ========

NUC2	13C
P3	16.00 usec
PL2	2.00 dB
SFO2	150.9454616 MHz

====== GRADIENT CHANNEL =====

GPNAM1	SINE.100
GPNAM2	SINE.100
GPNAM3	SINE.100
GPX1	0.00 %
GPX2	0.00 %
GPX3	0.00 %
GPY1	0.00 %
GPY2	0.00 %
GPY3	0.00 %
GPZ1	50.00 %
GPZ2	30.00 %
GPZ3	40.10 %
P16	2000.00 usec

F1 - Acquisition parameters

ND0	2
TD	256
SFO1	150.9455 MHz
FIDRES	136.821365 Hz
SW	232.046 ppm
FnMODE	QF

F2 - Processing parameters

SI	2048
SF	600.2306842 MHz
WDW	QSINE
SSB	8
LB	0.00 Hz
GB	0
PC	1.40

F1 - Processing parameters

SI	1024
MC2	QF
SF	150.9280949 MHz
WDW	QSINE
SSB	8
LB	0.00 Hz
GB	0

2D NMR plot parameters

CX2	20.00 cm
CX1	15.00 cm
F2PLO	9.347 ppm
F2LO	5610.65 Hz
F2PHI	0.406 ppm
F2HI	243.67 Hz
F1PLO	209.346 ppm
F1LO	31596.18 Hz
F1PHI	-0.970 ppm
F1HI	-146.38 Hz
F2PPMCM	0.44708 ppm/cm
F2HZCM	268.34930 Hz/cm
F1PPMCM	14.02105 ppm/cm
F1HZCM	2116.17065 Hz/cm

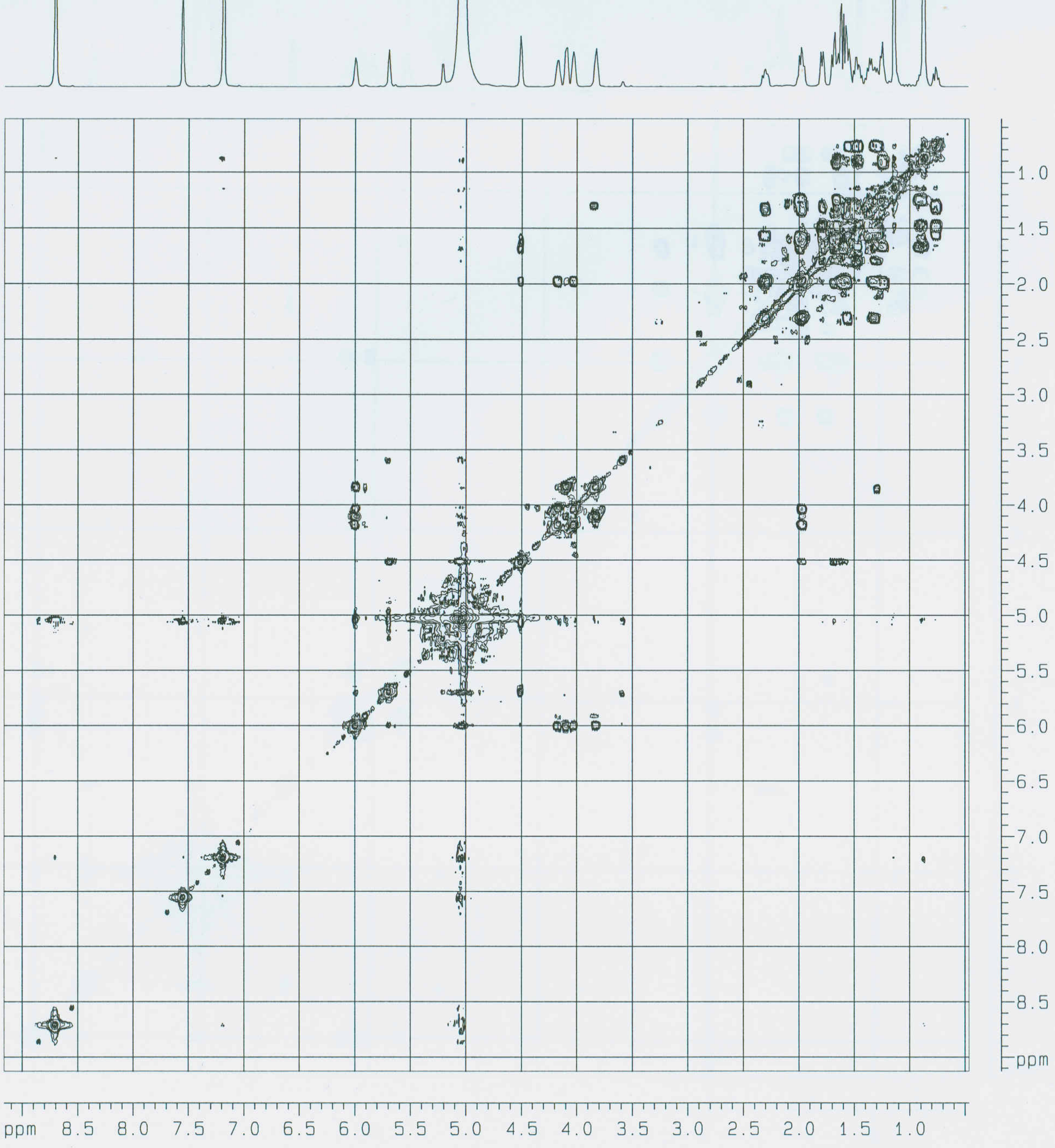

Current Data Parameters

NAME	August21
EXPNO	2
PROCNO	1

F2 - Acquisition Parameters

Date_	20080821
Time	10.46
INSTRUM	spect
PROBHD	5 mm CPTCI 1H-
PULPROG	cosygf45
TD	2048
SOLVENT	Pyr
NS	8
DS	4
SWH	6009.615 Hz
FIDRES	2.934382 Hz
AQ	0.1705268 sec
RG	28.5
DW	83.200 usec
DE	6.00 usec
TE	298.5 K
d0	0.00000300 sec
D1	1.50000000 sec
IN0	0.00016640 sec
MCREST	0.00000000 sec
MCWRK	1.50000000 sec

======== CHANNEL f1 ========

NUC1	1H
P1	7.40 usec
PL1	3.30 dB
SFO1	600.2337214 MHz

F1 - Acquisition parameters

ND0	1
TD	256
SFO1	600.2337 MHz
FIDRES	23.475060 Hz
SW	10.012 ppm
FnMODE	QF

F2 - Processing parameters

SI	1024
SF	600.2306842 MHz
WDW	SINE
SSB	0
LB	0.00 Hz
GB	0
PC	1.40

F1 - Processing parameters

SI	512
MC2	QF
SF	600.2306842 MHz
WDW	SINE
SSB	0
LB	0.00 Hz
GB	0

2D NMR plot parameters

CX2	15.00 cm
CX1	15.00 cm
F2PLO	9.167 ppm
F2LO	5502.08 Hz
F2PHI	0.465 ppm
F2HI	278.88 Hz
F1PLO	9.127 ppm
F1LO	5478.60 Hz
F1PHI	0.523 ppm
F1HI	314.09 Hz
F2PPMCM	0.58013 ppm/cm
F2HZCM	348.21341 Hz/cm
F1PPMCM	0.57361 ppm/cm
F1HZCM	344.30090 Hz/cm

[illegible]

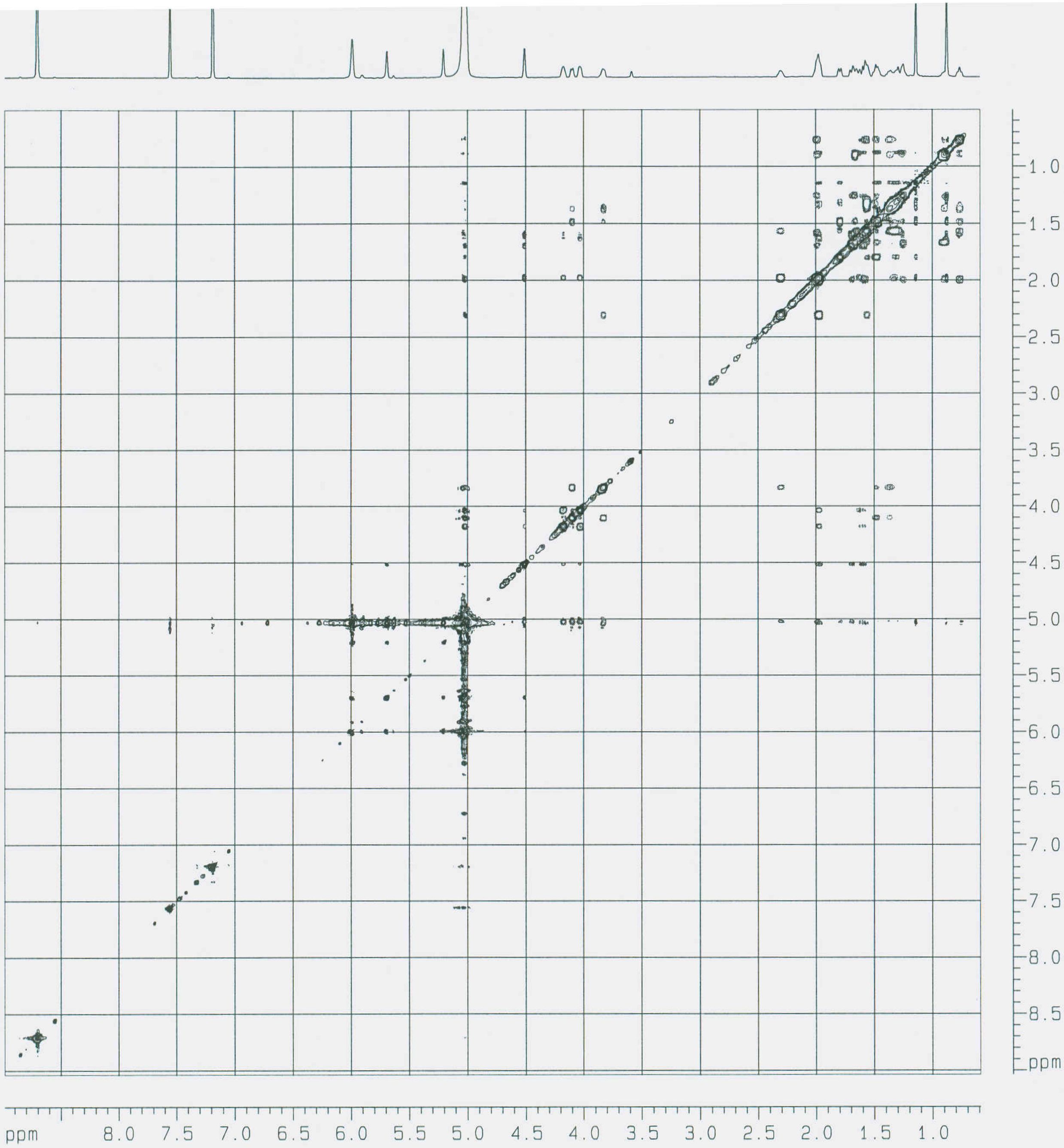

Current Data Parameters

NAME	August21
EXPNO	3
PROCNO	1

F2 - Acquisition Parameters

Date_	20080821
Time	11.44
INSTRUM	spect
PROBHD	5 mm CPTCI 1H-
PULPROG	noesyph
TD	2048
SOLVENT	Pyr
NS	8
DS	4
SWH	6009.615 Hz
FIDRES	2.934382 Hz
AQ	0.1705268 sec
RG	20.2
DW	83.200 usec
DE	6.00 usec
TE	299.1 K
d0	0.00007378 sec
D1	1.50000000 sec
D8	0.80000001 sec
IN0	0.00016640 sec
MCREST	0.00000000 sec
MCWRK	0.75000000 sec
ST1CNT	128

======== CHANNEL f1 ========

NUC1	1H
P1	7.40 usec
PL1	3.30 dB
SFO1	600.2337214 MHz

F1 - Acquisition parameters

ND0	1
TD	256
SFO1	600.2337 MHz
FIDRES	23.475060 Hz
SW	10.012 ppm
FnMODE	States-TPPI

F2 - Processing parameters

SI	2048
SF	600.2306842 MHz
WDW	QSINE
SSB	2
LB	0.00 Hz
GB	0
PC	1.00

F1 - Processing parameters

SI	1024
MC2	States-TPPI
SF	600.2306842 MHz
WDW	QSINE
SSB	2
LB	0.00 Hz
GB	0

2D NMR plot parameters

CX2	15.00 cm
CX1	15.00 cm
F2PLO	8.986 ppm
F2LO	5393.51 Hz
F2PHI	0.587 ppm
F2HI	352.24 Hz
F1PLO	9.040 ppm
F1LO	5425.79 Hz
F1PHI	0.504 ppm
F1HI	302.35 Hz
F2PPMCM	0.55993 ppm/cm
F2HZCM	336.08463 Hz/cm
F1PPMCM	0.56905 ppm/cm
F1HZCM	341.56216 Hz/cm

LAB. NO. 108

HEJ

2/7/2009

File: OX-4A
Sample: NAIK/H.E.J
Instrument: JEOL MSRoute
Inlet: Direct Probe

Date Run: 02-07-2009 (Time Run: 12:00:05)

Ionization mode: EI+

Scan: 64
Base: m/z 285; 15.7%FS TIC: 3250692 (Max Inten : 164548)

R.T.: 2.42

#Ions: 121

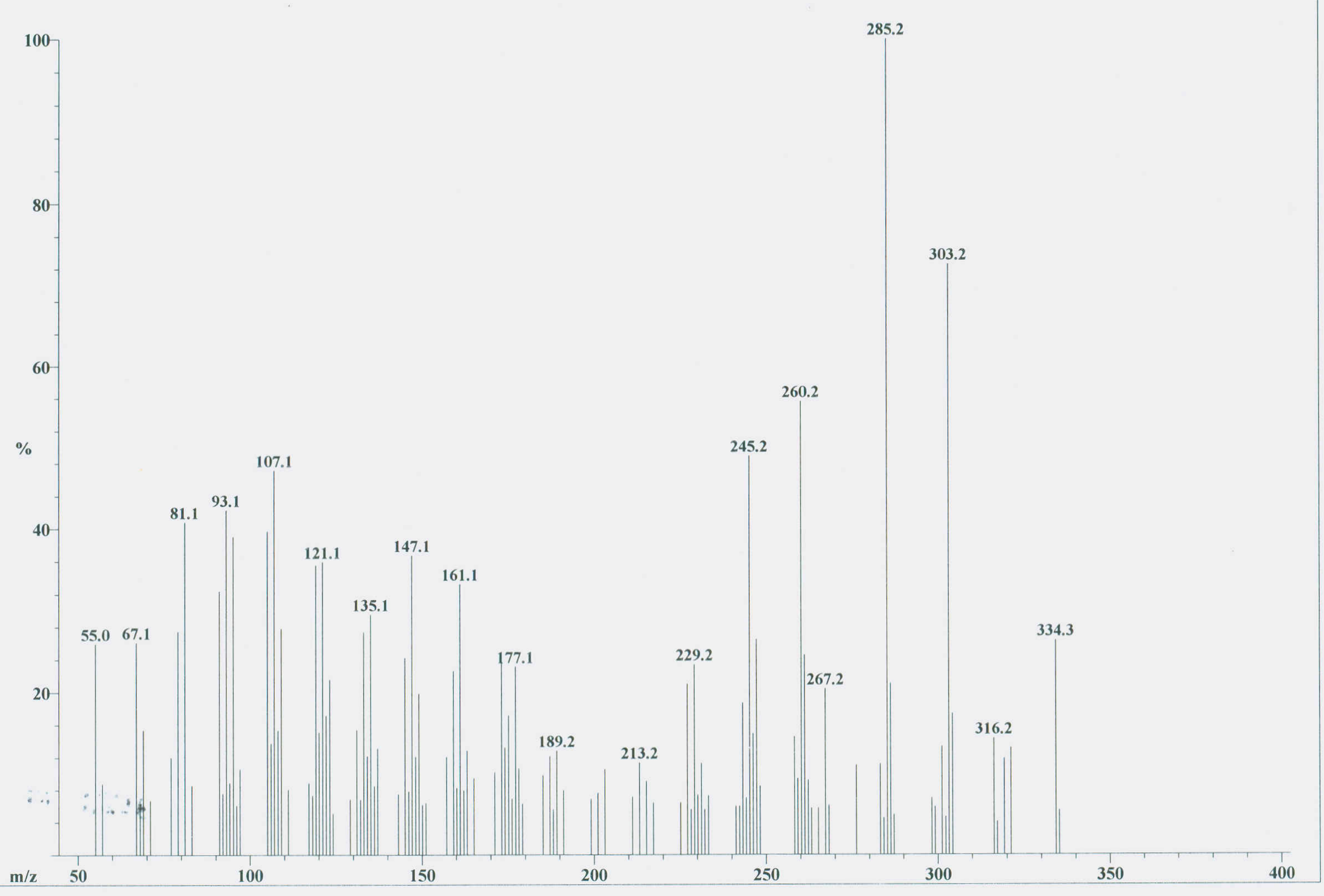

Mass	Relative Intensity	Theoretical Mass	Delta [ppm]	Delta [mmu]	RDB	Composition
309.9865	0.3384	309.9808	18.3	5.7	6.0	C[illegible] H[illegible] O[illegible]
312.2381	0.2453					
312.2725	0.1567					
312.9848	0.2521	312.9832	5.2	1.6	9.5	$C_{11}H_{5}O_{11}$
		312.9891	-13.6	-4.2	0.5	$C_{4}H_{9}O_{16}$
314.2217	0.6752	314.2246	-9.3	-2.9	7.0	$C_{21}H_{30}O_{2}$
315.2243	0.1962					
316.2337	0.6086					
316.9807	1.2960	316.9781	8.2	2.6	8.5	$C_{10}H_{5}O_{12}$
317.2368	0.3055	317.2328	12.7	4.0	1.5	$C_{17}H_{33}O_{5}$
318.0935	0.3873	318.0951	-5.0	-1.6	5.0	$C_{13}H_{18}O_{9}$
		318.0892	13.4	4.3	14.0	$C_{20}H_{14}O_{4}$
318.9823	0.9505	318.9785	11.8	3.8	3.5	$C_{6}H_{7}O_{15}$
319.0184	0.2496	319.0149	10.9	3.5	2.5	$C_{7}H_{11}O_{14}$
319.0242	0.2142	319.0301	-18.7	-6.0	6.5	$C_{11}H_{11}O_{11}$
319.2225	0.3706	319.2273	-15.2	-4.9	5.5	$C_{20}H_{31}O_{3}$
321.2492	0.4317					
326.9820	0.1385	326.9836	-4.9	-1.6	5.5	$C_{8}H_{7}O_{14}$
		326.9777	13.1	4.3	14.5	$C_{15}H_{3}O_{9}$
326.9861	0.1554	326.9836	7.8	2.6	5.5	$C_{8}H_{7}O_{14}$
328.9816	0.5175	328.9840	-7.1	-2.3	0.5	$C_{4}H_{9}O_{17}$
		328.9781	10.7	3.5	9.5	$C_{11}H_{5}O_{12}$
330.9787	4.4707	330.9785	0.6	0.2	4.5	$C_{7}H_{7}O_{15}$
333.2270	0.2305	333.2277	-2.2	-0.7	1.5	$C_{17}H_{33}O_{6}$
		333.2218	15.4	5.1	10.5	$C_{24}H_{29}O_{1}$
334.2476	0.7175	334.2508	-9.5	-3.2	5.0	$C_{21}H_{34}O_{3}$
339.2723	0.1616	339.2688	10.4	3.5	7.5	$C_{24}H_{35}O_{1}$
340.9860	0.3410	340.9840	5.9	2.0	1.5	$C_{5}H_{9}O_{17}$
342.9787	2.7963	342.9785	0.6	0.2	5.5	$C_{8}H_{7}O_{15}$
352.2643	0.1491	352.2614	8.2	2.9	4.0	$C_{21}H_{36}O_{4}$
354.9787	1.5436	354.9785	0.6	0.2	6.5	$C_{9}H_{7}O_{15}$

----> for sample peak, [illegible].S. -1/40, Def. 8.0 m[illegible]u, Formula, up to 1461 mass
C 2/100, H 0/100, O 0/1[illegible]

----> for standard peak, U.S. 0/10, Def. 5.0 mmu, Formula, up to 495 mass
C 0/20, H 0/1, F 0/30

Mass	Int.	mmu	U.S.	No:	Elemental Formula
351.2551	100.00	1.6	4.5	1:	C21 H35 O4

OX4A.

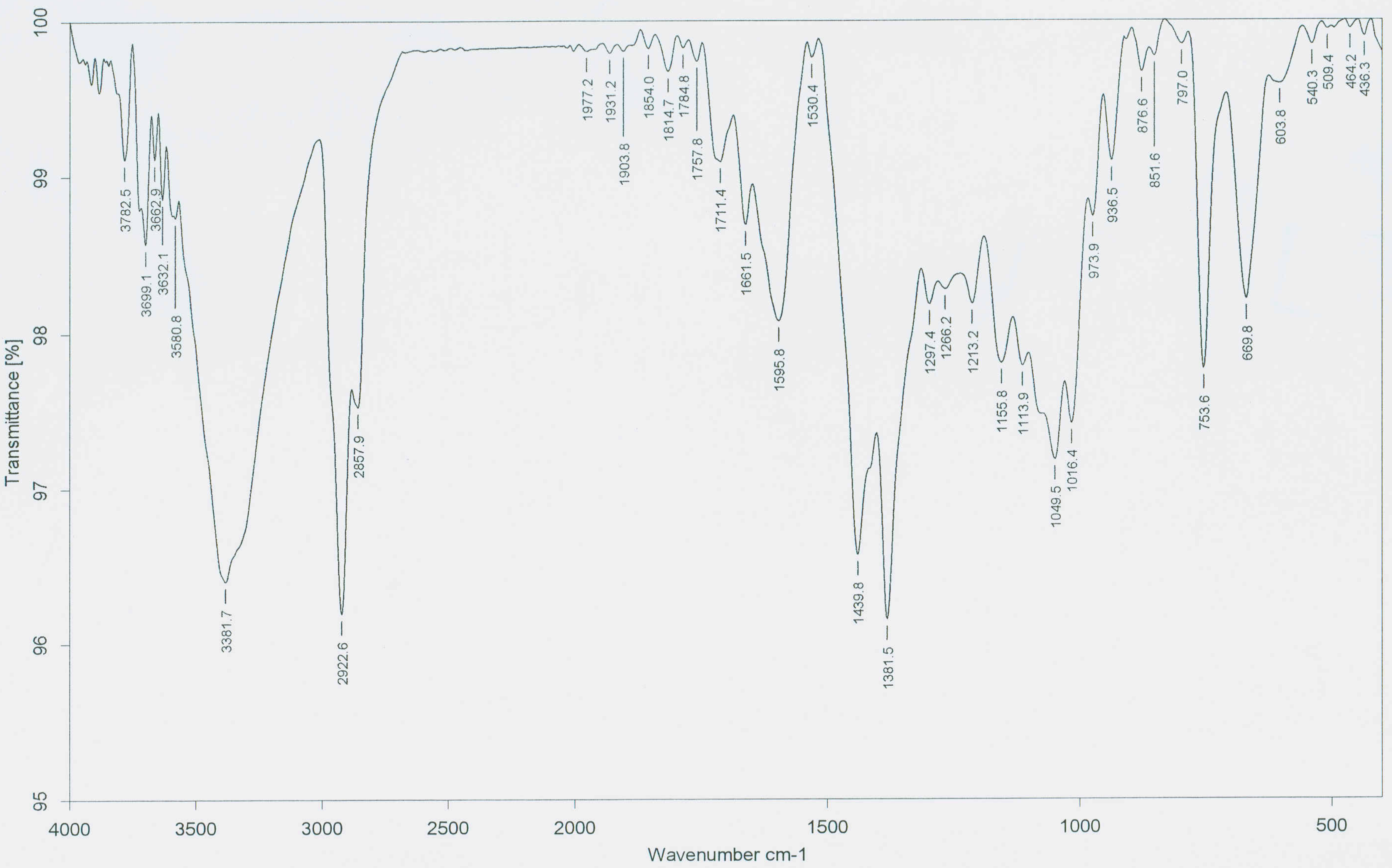

Transmittance [%]
100
99
98
97
96
95
4000
3500
3000
2500
2000
1500
1000
500
Wavenumber cm-1
3782.5
3699.1
3662.9
3632.1
3580.8
3381.7
2922.6
2857.9
1977.2
1931.2
1903.8
1854.0
1814.7
1784.8
1757.8
1711.4
1661.5
1595.8
1530.4
1439.8
1381.5
1297.4
1266.2
1213.2
1155.8
1113.9
1049.5
1016.4
973.9
936.5
876.6
851.6
797.0
753.6
669.8
603.8
540.3
509.4
464.2
436.3
