# Supplementary material for: Synthesis of some potent immunomodulatory and anti-inflammatory metabolites by fungal transformation of anabolic steroid oxymetholone
Source: Chem Cent J. 2012 Dec 10;6:153. doi: 10.1186/1752-153X-6-153 (PMC3740782; doi:10.1186/1752-153X-6-153)
Supplement: Additional file 3 — Spectroscopic data of compound 3. Include spectra of 1H-NMR, 13C-NMR (BB, DEPT-135), HSQC, HMBC, COSY-45°, NOESY, EI-MS, HREI-MS, and IR experiments. [file 1752-153X-6-153-S3.pdf]

[illegible]

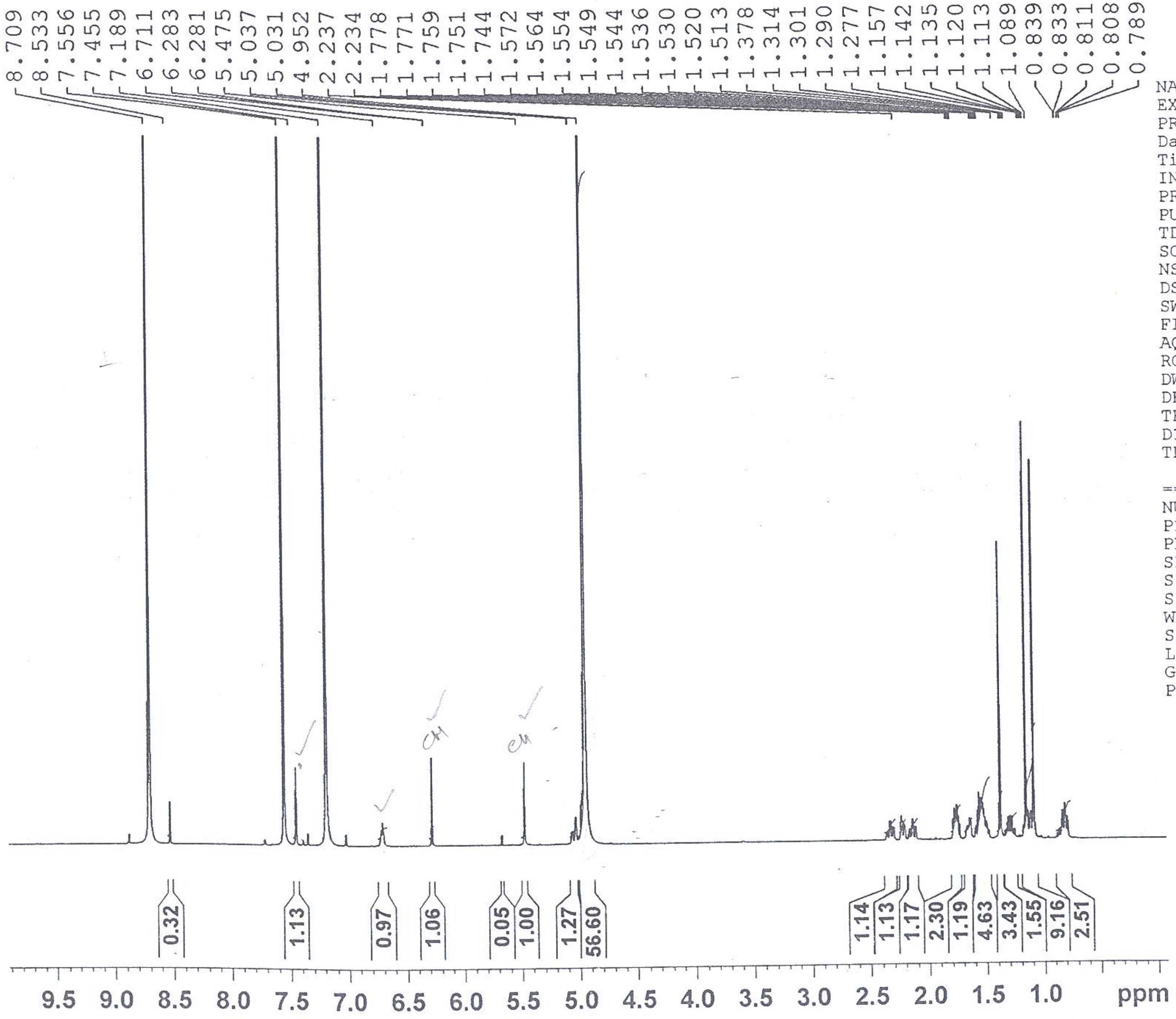

NAME	mar16
EXPNO	2
PROCNO	1
Date_	20100316
Time	12.02
INSTRUM	spect
PROBHD	5 mm BBI 1H-BB
PULPROG	zg30
TD	32768
SOLVENT	Pyr
NS	128
DS	0
SWH	10000.000 Hz
FIDRES	0.305176 Hz
AQ	1.6385000 sec
RG	228.1
DW	50.000 usec
DE	6.50 usec
TE	302.2 K
D1	1.00000000 sec
TD0	1

======== CHANNEL f1 ========

NUC1	1H
P1	6.70 usec
PL1	6.00 dB
SFO1	500.1340010 MHz
SI	32768
SF	500.1299985 MHz
WDW	EM
SSB	0
LB	0.30 Hz
GB	0
PC	1.00

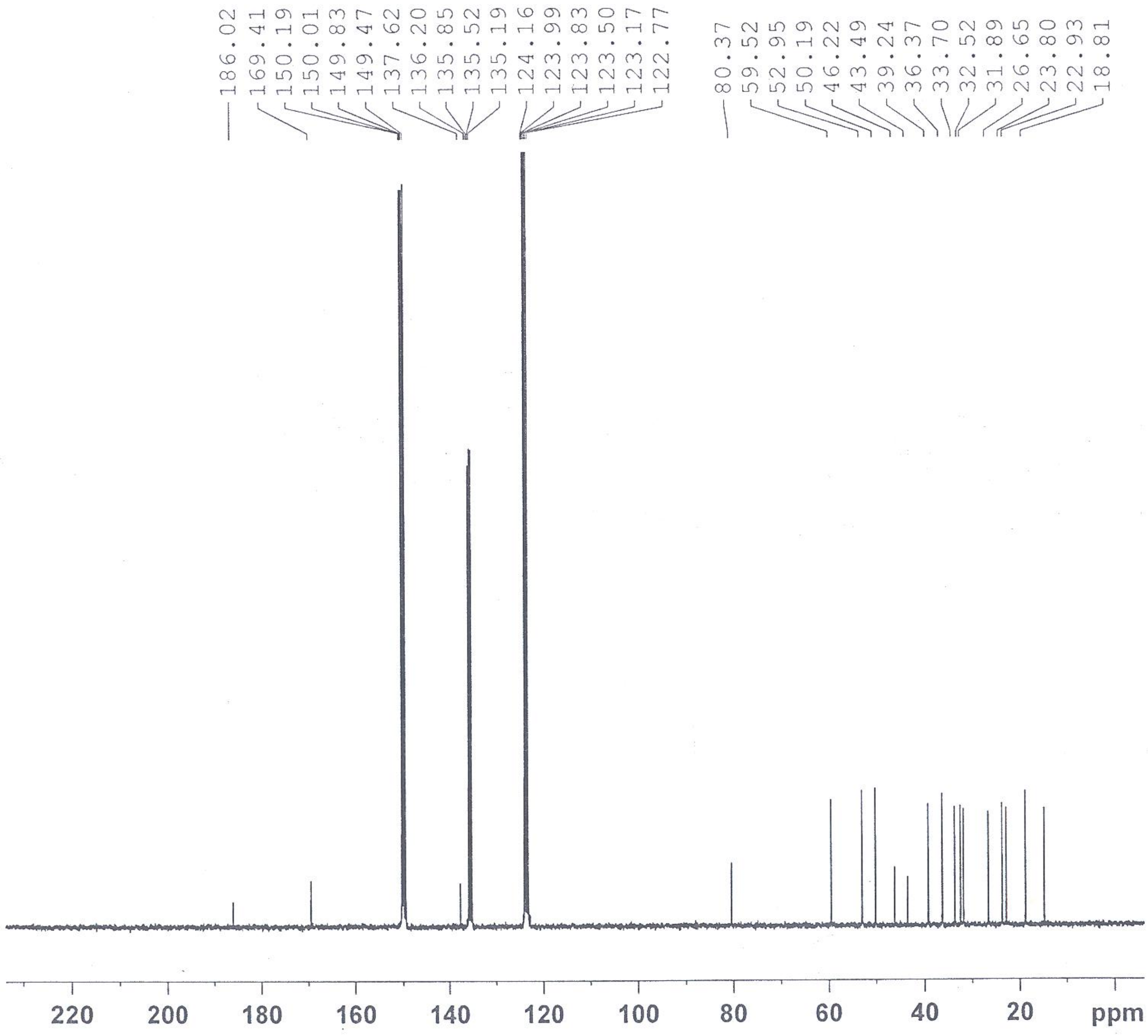

BRUKER

NAME	Apr 15
EXPNO	1
PROCNO	1
Date_	20100415
Time_	11.10
INSTRUM	spect
PROBHD	5 mm DUL 13C-1
PULPROG	zgpg
TD	32768
SOLVENT	Pyr
NS	7977
DS	2
SWH	18115.941 Hz
FIDRES	0.552855 Hz
AQ	0.9044468 sec
RG	32768
DW	27.600 usec
DE	6.50 usec
TE	297.2 K
D1	1.50000000 sec
D11	0.03000000 sec
TD0	18

======== CHANNEL f1 ========

NUC1	13C
P1	9.20 usec
PL1	4.00 dB
SFO1	75.4764278 MHz

======== CHANNEL f2 ========

CPDPRG2	waltz16
NUC2	1H
PCPD2	80.00 usec
PL2	5.00 dB
PL12	20.92 dB
PL13	19.00 dB
SFO2	300.1318008 MHz
SI	32768
SF	75.4678462 MHz
WDW	EM
SSB	0
LB	1.00 Hz
GB	0
PC	1.40

Sample: [illegible]

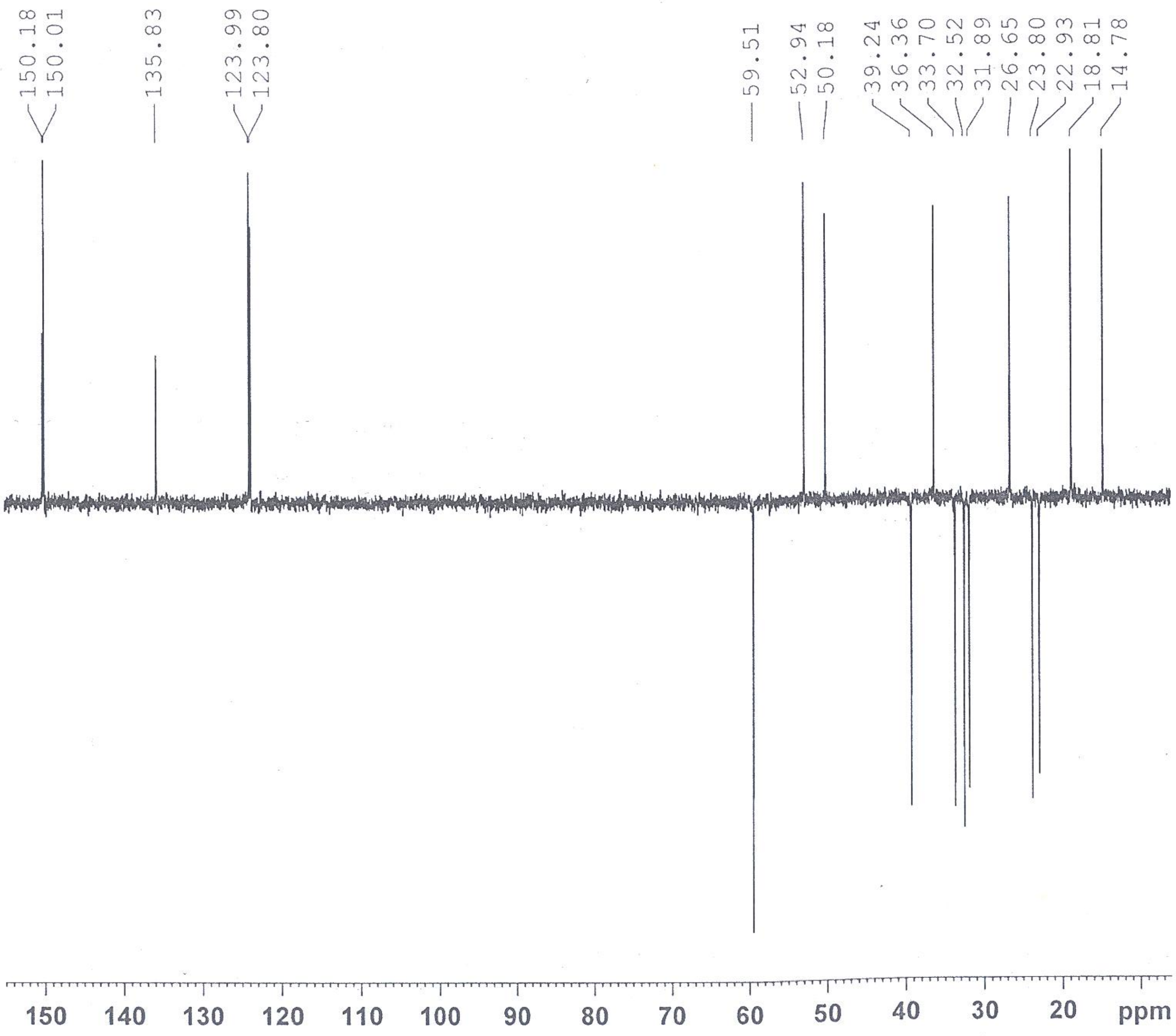

BRUKER

NAME	Apr 15
EXPNO	2
PROCNO	1
Date_	20100415
Time_	16.06
INSTRUM	spect
PROBHD	5 mm DUL 13C-1
PULPROG	dept135
TD	32768
SOLVENT	Pyr
NS	9216
DS	2
SWH	15060.241 Hz
FIDRES	0.459602 Hz
AQ	1.0879476 sec
RG	32768
DW	33.200 usec
DE	6.50 usec
TE	297.2 K
CNST2	145.0000000
D1	1.50000000 sec
D2	0.00344828 sec
D12	0.00002000 sec
TD0	9

======== CHANNEL f1 ========

NUC1	13C
P1	9.20 usec
P2	18.40 usec
PL1	4.00 dB
SFO1	75.4752203 MHz

======== CHANNEL f2 ========

CPDPRG2	waltz16
NUC2	1H
P3	12.80 usec
P4	25.60 usec
PCPD2	80.00 usec
PL2	5.00 dB
PL12	20.92 dB
SFO2	300.1315007 MHz
SI	32768
SF	75.4678462 MHz
WDW	EM
SSB	0
LB	1.00 Hz
GB	0
PC	1.40

LAB. No. 108

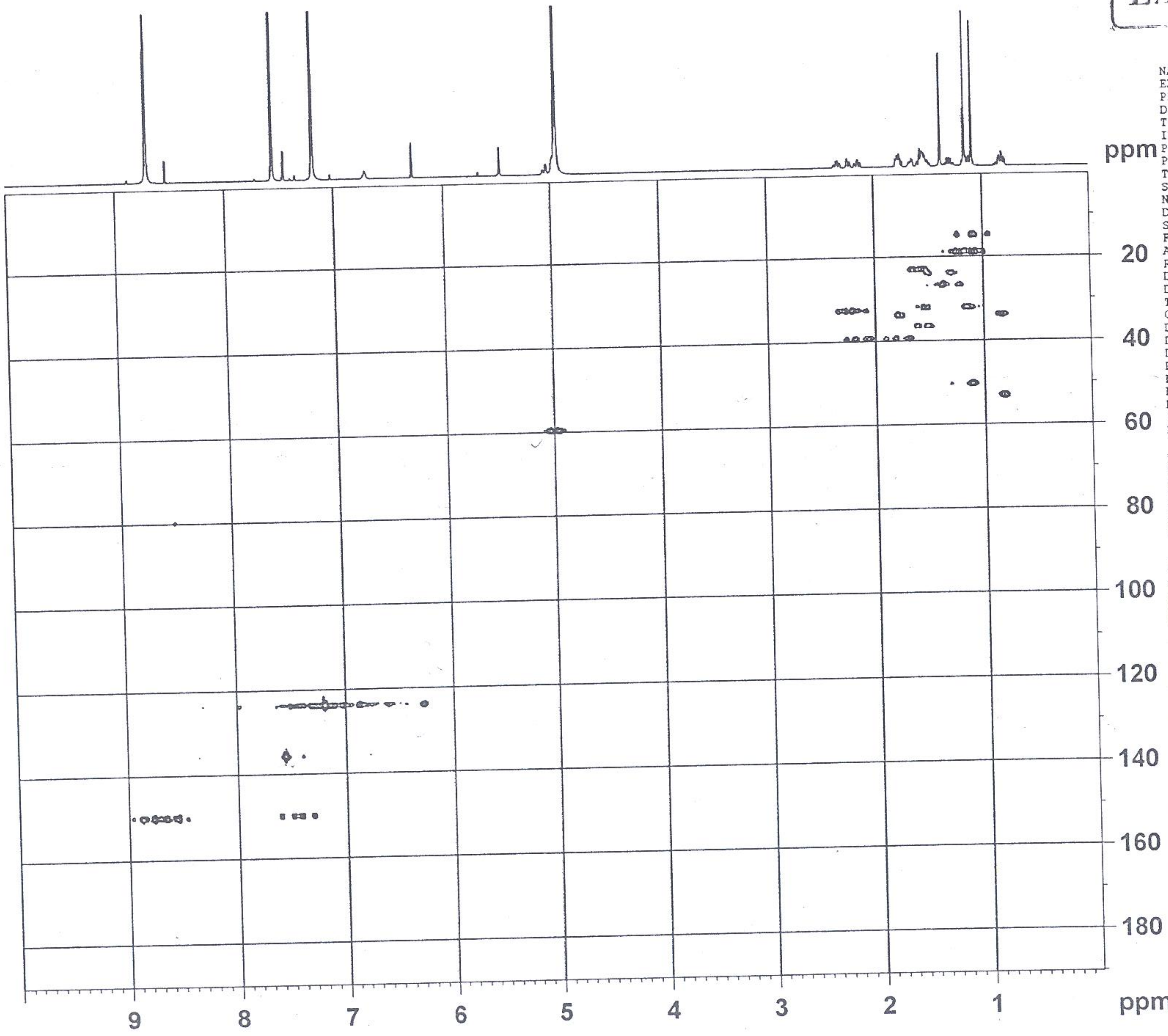

NAME	mar17
EXPNO	12
PROCNO	1
Date_	20100317
Time	17.13
INSTRUM	spect
PROBHD	5 mm BBI 1H-BB
PULPROG	hsqcetgpsi
TD	1024
SOLVENT	Pyr
NS	32
DS	8
SWH	5000.000 Hz
FIDRES	4.882813 Hz
AQ	0.1025500 sec
RG	26008
DW	100.000 usec
DE	6.50 usec
TE	305.2 K
CNST2	145.0000000
D0	0.00000300 sec
D1	1.50000000 sec
D4	0.00172414 sec
D11	0.03000000 sec
D13	0.00000400 sec
D16	0.00015000 sec
D24	0.00110000 sec
IN0	0.00002090 sec
ZGOPTNS	
======== CHANNEL f1 ========	
NUC1	1H
P1	6.70 usec
P2	13.40 usec
P28	1000.00 usec
PL1	6.00 dB
SFO1	500.1325007 MHz
======== CHANNEL f2 ========	
CPDPRG2	garp
NUC2	13C
P3	11.10 usec
P4	22.20 usec
PCPD2	100.00 usec
PL2	-3.00 dB
PL12	18.00 dB
SFO2	125.7697360 MHz
====== GRADIENT CHANNEL =====	
GPNAM1	SINE.100
GPNAM2	SINE.100
GPZ1	80.00 %
GPZ2	20.10 %
P16	1500.00 usec
ND0	2
TD	256
SFO1	125.7697 MHz
FIDRES	93.344727 Hz
SW	190.000 ppm
FnMODE	Echo-Antiecho
SI	1024
SF	500.1299984 MHz
WDW	QSINE
SSB	2
LB	0.00 Hz
GB	0
PC	4.00
SI	1024
MC2	echo-antiecho
SF	125.7577603 MHz
WDW	QSINE
SSB	2
LB	0.00 Hz
GB	0

LAB No. 109-B

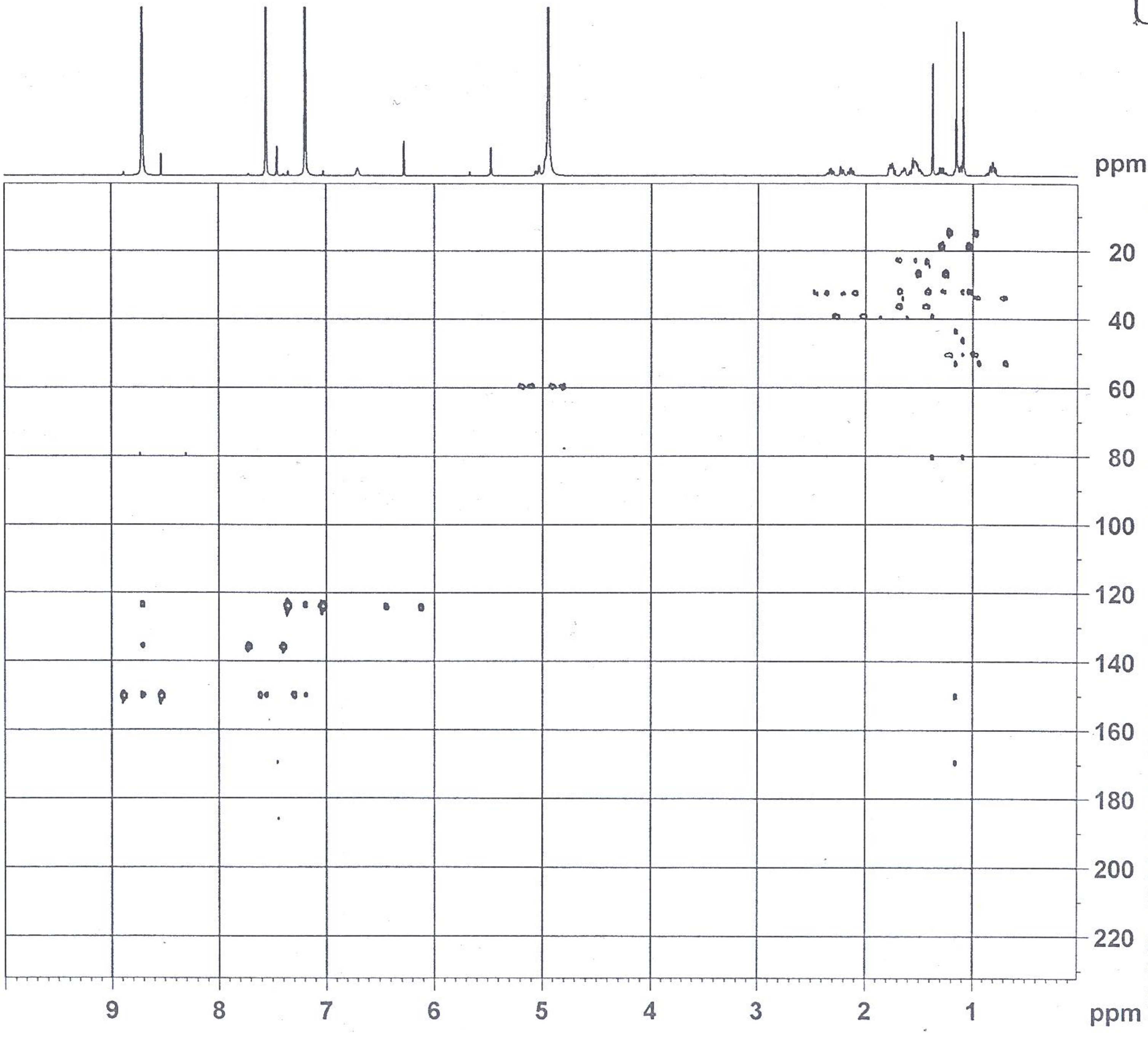

NAME	mar17	
EXPNO	13	
PROCNO	1	
Date_	20100317	
Time	20.56	
INSTRUM	spect	
PROBHD	5 mm BBI 1H-BB	
PULPROG	hmbcgpndqf	
TD	4096	
SOLVENT	Pyr	
NS	64	
DS	8	
SWH	5000.000	Hz
FIDRES	1.220703	Hz
AQ	0.4097500	sec
RG	23170.5	
DW	100.000	usec
DE	6.50	usec
TE	305.4	K
CNST13	145.0000000	
D0	0.00000300	sec
D1	1.50000000	sec
D6	0.00344828	sec
D16	0.00020000	sec
IN0	0.00001715	sec
======== CHANNEL f1 ========		
NUC1	1H	
P1	6.70	usec
P2	13.40	usec
PL1	6.00	dB
SFO1	500.1325007	MHz
======== CHANNEL f2 ========		
NUC2	13C	
P3	11.10	usec
PL2	-3.00	dB
SFO2	125.7723769	MHz
====== GRADIENT CHANNEL =====		
GPNAM1	SINE.100	
GPNAM2	SINE.100	
GPNAM3	SINE.100	
GPZ1	50.00	%
GPZ2	30.00	%
GPZ3	40.10	%
P16	1000.00	usec
ND0	2	
TD	256	
SFO1	125.7724	MHz
FIDRES	113.981216	Hz
SW	232.000	ppm
FnMODE	QF	
SI	1024	
SF	500.1299984	MHz
WDW	QSINE	
SSB	0	
LB	0.00	Hz
GB	0	
PC	4.00	
SI	1024	
MC2	QF	
SF	125.7577603	MHz
WDW	QSINE	
SSB	0	
LB	0.00	Hz
GB	0	

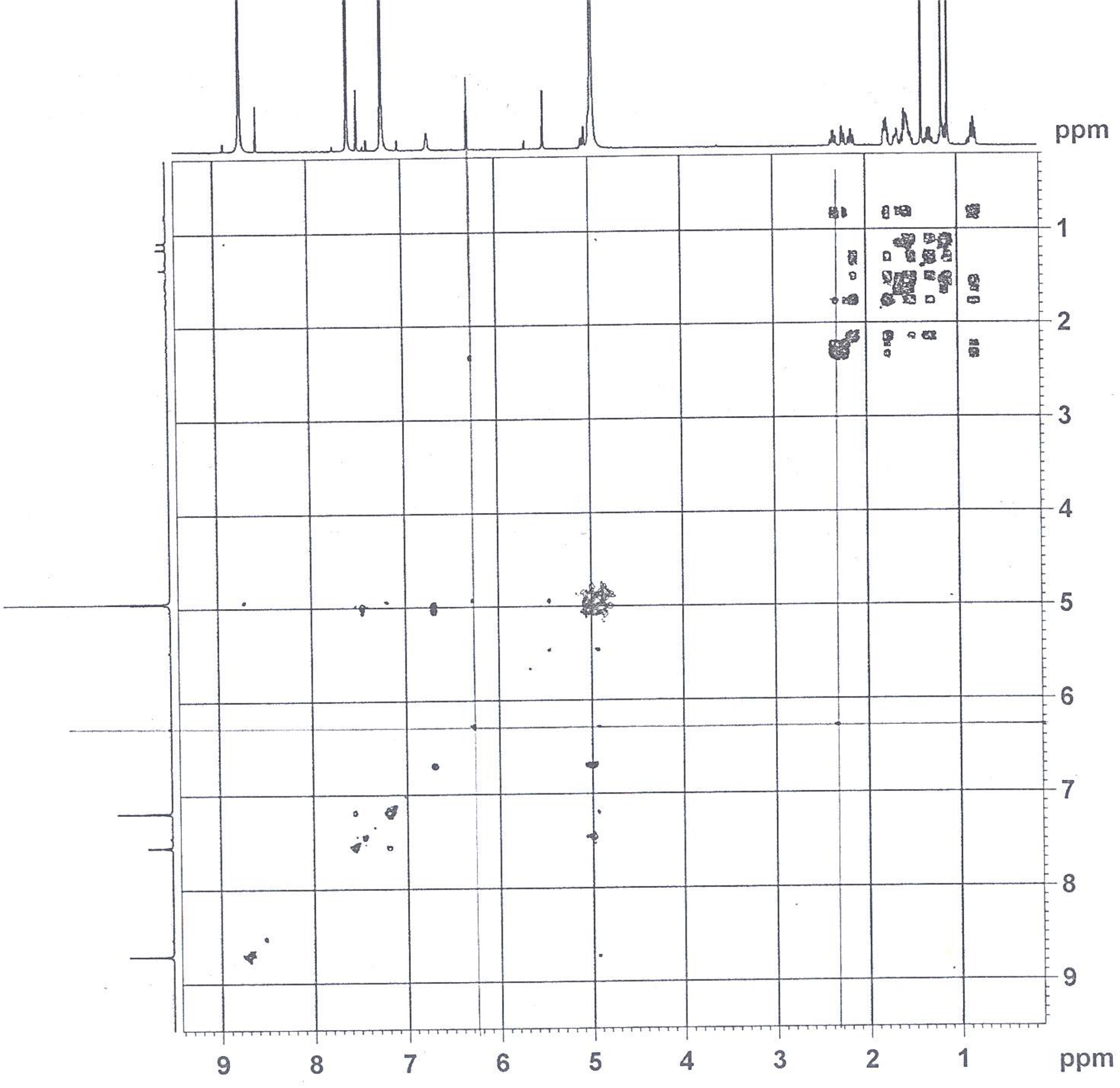

NAME	mar17
EXPNO	10
PROCNO	1
Date_	20100317
Time	13.20
INSTRUM	spect
PROBHD	5 mm BBI 1H-BB
PULPROG	cosydfqf
TD	2048
SOLVENT	Pyr
NS	8
DS	8
SWH	5000.000 Hz
FIDRES	2.441406 Hz
AQ	0.2049500 sec
RG	256
DW	100.000 usec
DE	6.50 usec
TE	302.9 K
D0	0.00000300 sec
D1	1.50000000 sec
D13	0.00000400 sec
D20	0.00000200 sec
IN0	0.00020000 sec

======== CHANNEL f1 ========

NUC1	1H
P1	6.70 usec
PL1	6.00 dB
SFO1	500.1325007 MHz
ND0	1
TD	256
SFO1	500.1325 MHz
FIDRES	19.531250 Hz
SW	9.997 ppm
FnMODE	QF
SI	1024
SF	500.1299984 MHz
WDW	SINE
SSB	0
LB	0.00 Hz
GB	0
PC	4.00
SI	1024
MC2	QF
SF	500.1299984 MHz
WDW	SINE
SSB	0
LB	0.00 Hz
GB	0

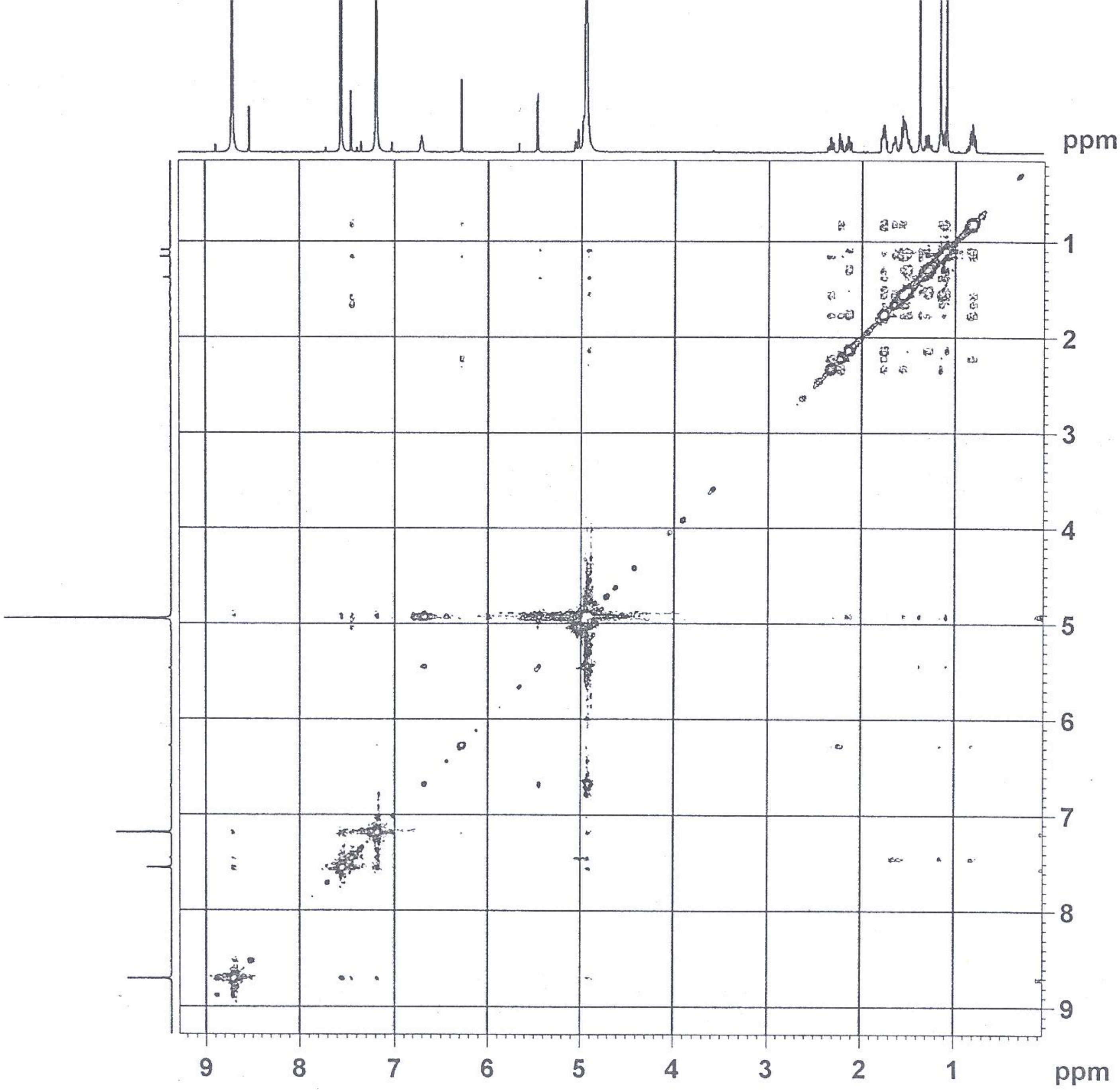

NAME	mar17
EXPNO	11
PROCNO	1
Date_	20100317
Time_	14.20
INSTRUM	spect
PROBHD	5 mm BBI 1H-BB
PULPROG	noesygpph
TD	2048
SOLVENT	Pyr
NS	16
DS	2
SWH	5000.000 Hz
FIDRES	2.441406 Hz
AQ	0.2049500 sec
RG	812.7
DW	100.000 usec
DE	6.50 usec
TE	304.5 K
D0	0.00009147 sec
D1	1.50000000 sec
D8	0.80000001 sec
D16	0.00020000 sec
IN0	0.00020000 sec
======== CHANNEL f1 ========	
NUC1	1H
P1	6.70 usec
P2	13.40 usec
PL1	6.00 dB
SFO1	500.1325007 MHz
====== GRADIENT CHANNEL =====	
GPNAM1	SINE.100
GPNAM2	SINE.100
GPZ1	40.00 %
GPZ2	-40.00 %
P16	1000.00 usec
ND0	1
TD	256
SFO1	500.1325 MHz
FIDRES	19.531250 Hz
SW	9.997 ppm
FnMODE	States-TPPI
SI	1024
SF	500.1299984 MHz
WDW	QSINE
SSB	2
LB	0.00 Hz
GB	0
PC	4.00
SI	1024
MC2	States-TPPI
SF	500.1299984 MHz
WDW	QSINE
SSB	2
LB	0.00 Hz
GB	0

File: OMF-4
Sample: MARIUM
Instrument: JEOL MSRoute
Inlet: Direct Probe

Date Run: 03-27-2010 (Time Run: 11:45:52)

Ionization mode: EI+

Scan: 26
Base: m/z 134; 99.8%FS TIC: 12195756 (Max Inten : 1046043)

R.T.: 2.22

#Ions: 212

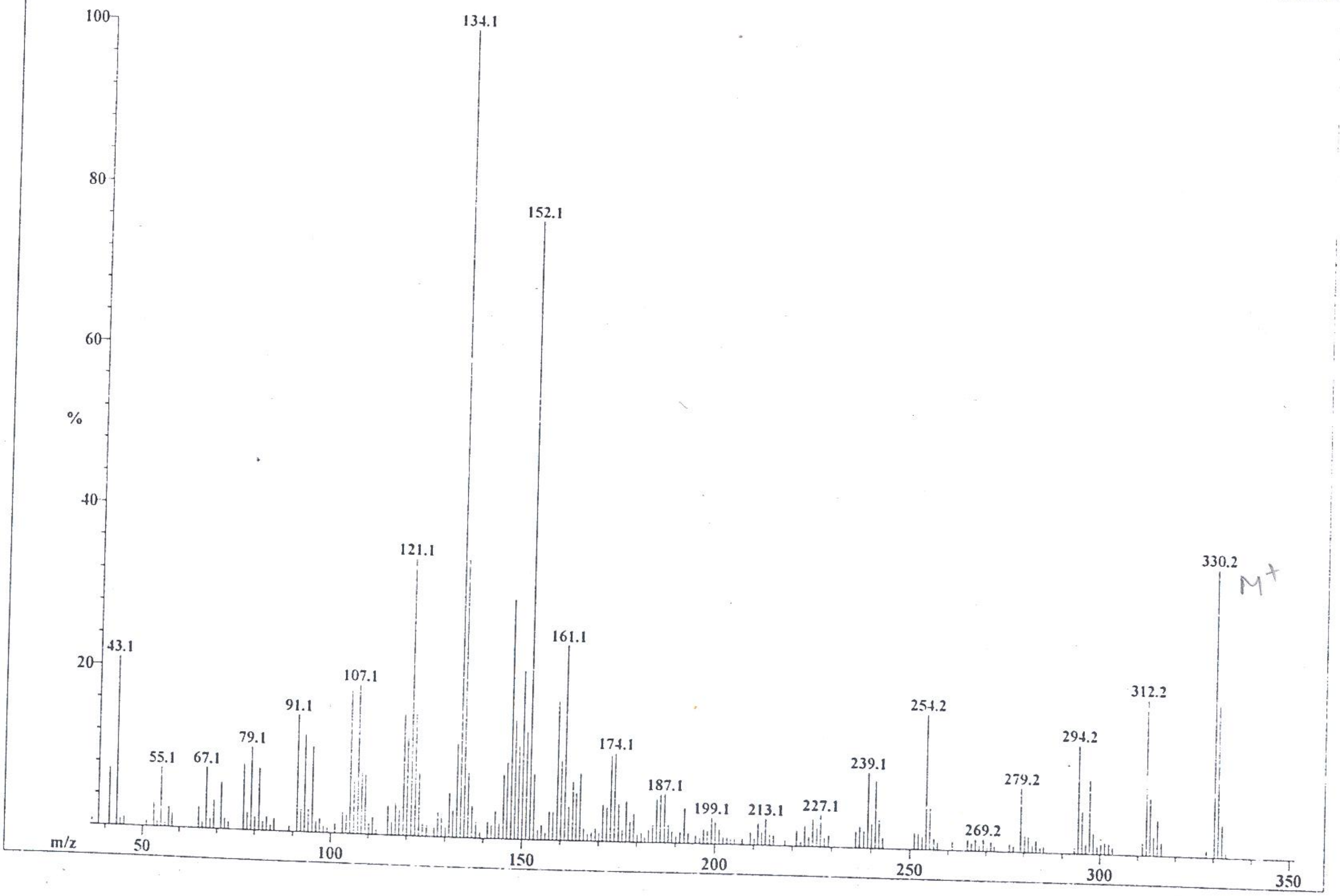

Mass	Relative Intensity	Theoretical Mass	Delta [ppm]	Delta [mmu]	RDB	Composition
279.1770	11.4654	279.1749	7.6	2.1	9.5	$C_{20} H_{23} O_{1}$
280.1803	3.1230	280.1827	-8.5	-2.4	9.0	$C_{20} H_{24} O_{1}$
280.9819	6.1515					
281.1895	2.4487	281.1905	-3.6	-1.0	8.5	$C_{20} H_{25} O_{1}$
282.1980	5.8308	282.1984	-1.2	-0.3	8.0	$C_{20} H_{26} O_{1}$
292.9819	3.9573					
293.1626	2.1839					
293.1841	2.0115					
294.1988	12.9178	294.1984	1.4	0.4	9.0	$C_{21} H_{26} O_{1}$
295.1761	6.3851					
296.2098	3.8371	296.2140	-14.4	-4.3	8.0	$C_{21} H_{28} O_{1}$
297.1875	6.6315	297.1855	6.9	2.0	8.5	$C_{20} H_{25} O_{2}$
298.1911	2.3555	298.1933	-7.5	-2.2	8.0	$C_{20} H_{26} O_{2}$
300.2096	6.0528	300.2089	2.4	0.7	7.0	$C_{20} H_{28} O_{2}$
310.1950	6.6282	310.1933	5.6	1.7	9.0	$C_{21} H_{26} O_{2}$
311.1976	2.5403	311.2011	-11.1	-3.5	8.5	$C_{21} H_{27} O_{2}$
312.2079	10.6525	312.2089	-3.3	-1.0	8.0	$C_{21} H_{28} O_{2}$
313.1878	4.8427					
314.2220	3.3635	314.2246	-8.4	-2.6	7.0	$C_{21} H_{30} O_{2}$
315.1967	2.3920	315.1960	2.1	0.7	7.5	$C_{20} H_{27} O_{3}$
328.2032	3.9015	328.2038	-1.9	-0.6	8.0	$C_{21} H_{28} O_{3}$
330.2183	7.1294	330.2195	-3.6	-1.2	7.0	$C_{21} H_{30} O_{3}$
330.9787	3.4826					
342.9787	2.4331					

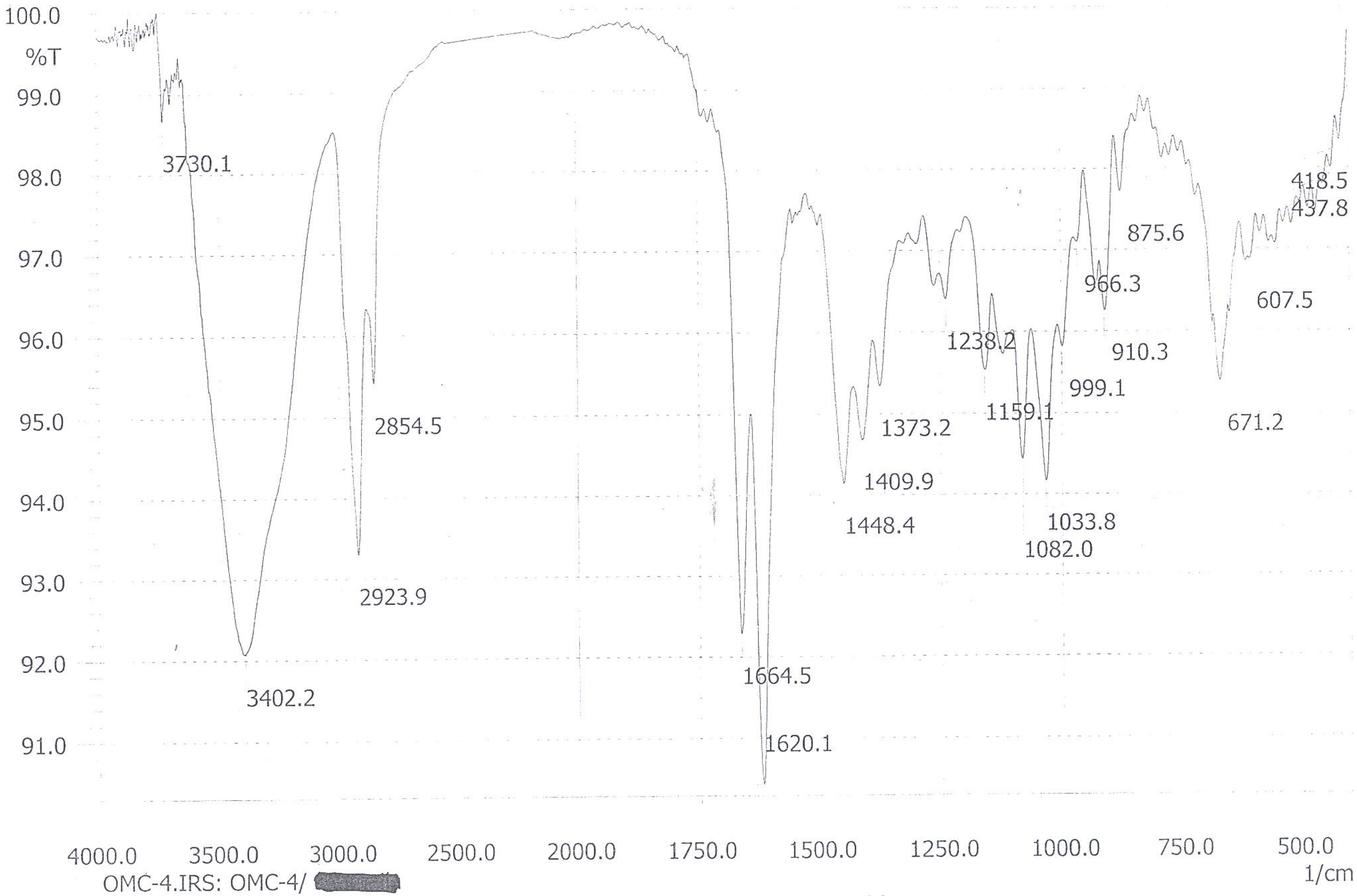

OMC-4.IRS: OMC-4/ [redacted]

Date:	05/10/2010	Time:	15:01:32	NScans:	20
Type:	HYPER IR	User:	Zubair Ahmad	Detector:	standard
Abscissa:	1/cm	Ordinate:	%T	Apodization:	Happ
Min:	401.17	Max:	3998.16	Range:	1/cm
Ndp:	1866	Data Interval:	1.92868	Resolution:	4.0
Gain:	auto	Aperture:	auto	Mirror Speed:	2.8(low)

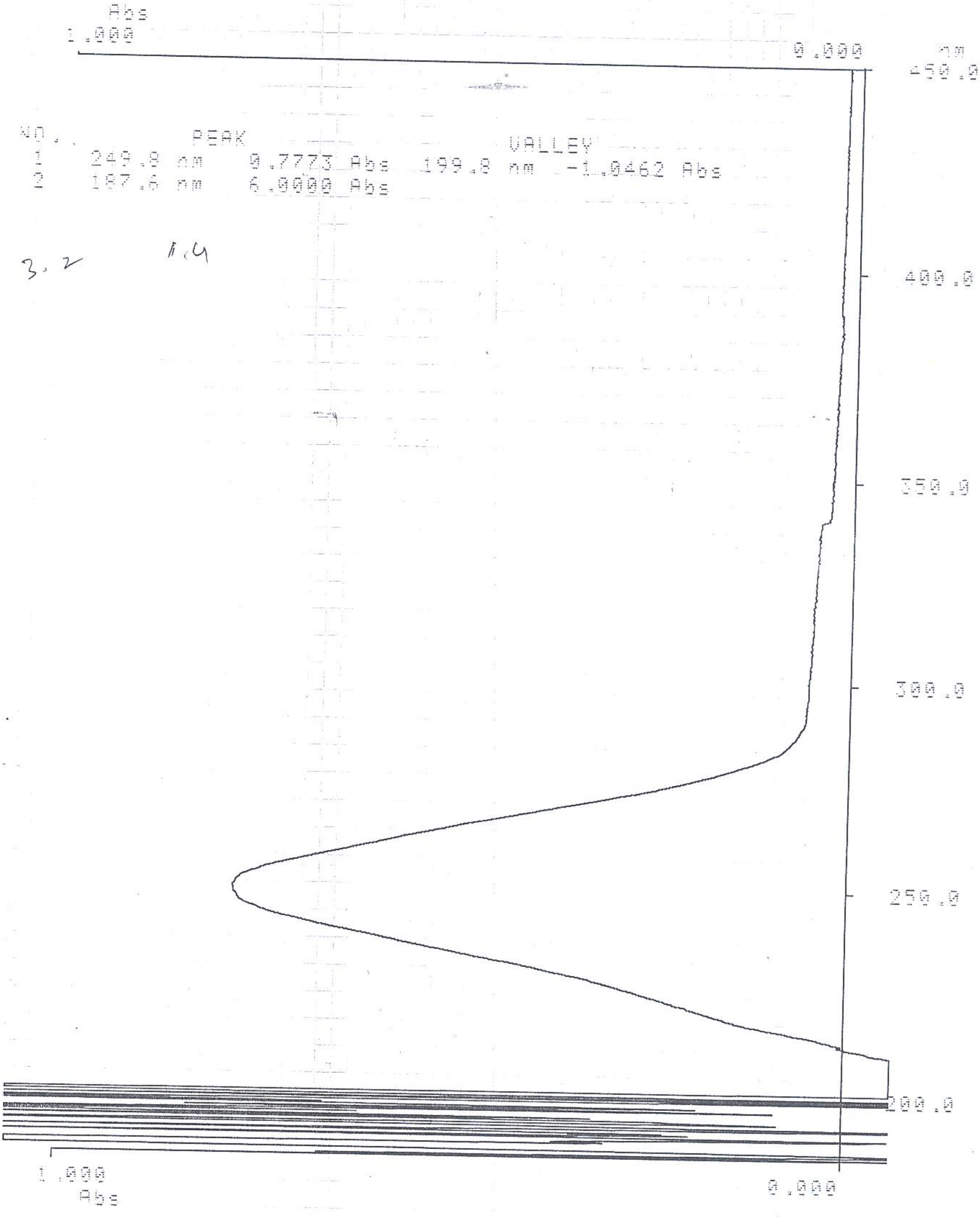

NO.	PEAK		VALLEY	
1	249.8 nm	0.7773 Abs	199.8 nm	-1.0462 Abs
2	187.6 nm	6.0000 Abs		

3.2 1.4

TITLE: FTSHAD/DMC-4
SCAN SPEED: 120.0 nm/min
BANDPASS: 2.00nm

9:50 AM 9/27/10
RESPONSE: MEDIUM
