# Supplementary material for: Synthesis of some potent immunomodulatory and anti-inflammatory metabolites by fungal transformation of anabolic steroid oxymetholone
Source: Chem Cent J. 2012 Dec 10;6:153. doi: 10.1186/1752-153X-6-153 (PMC3740782; doi:10.1186/1752-153X-6-153)
Supplement: Additional file 4 — Spectroscopic data of compound 6. Include spectra of 1H-NMR, 13C-NMR (BB, DEPT-135), HMQC, HMBC, COSY-45°, NOESY, and EI-MS. [file 1752-153X-6-153-S4.pdf]

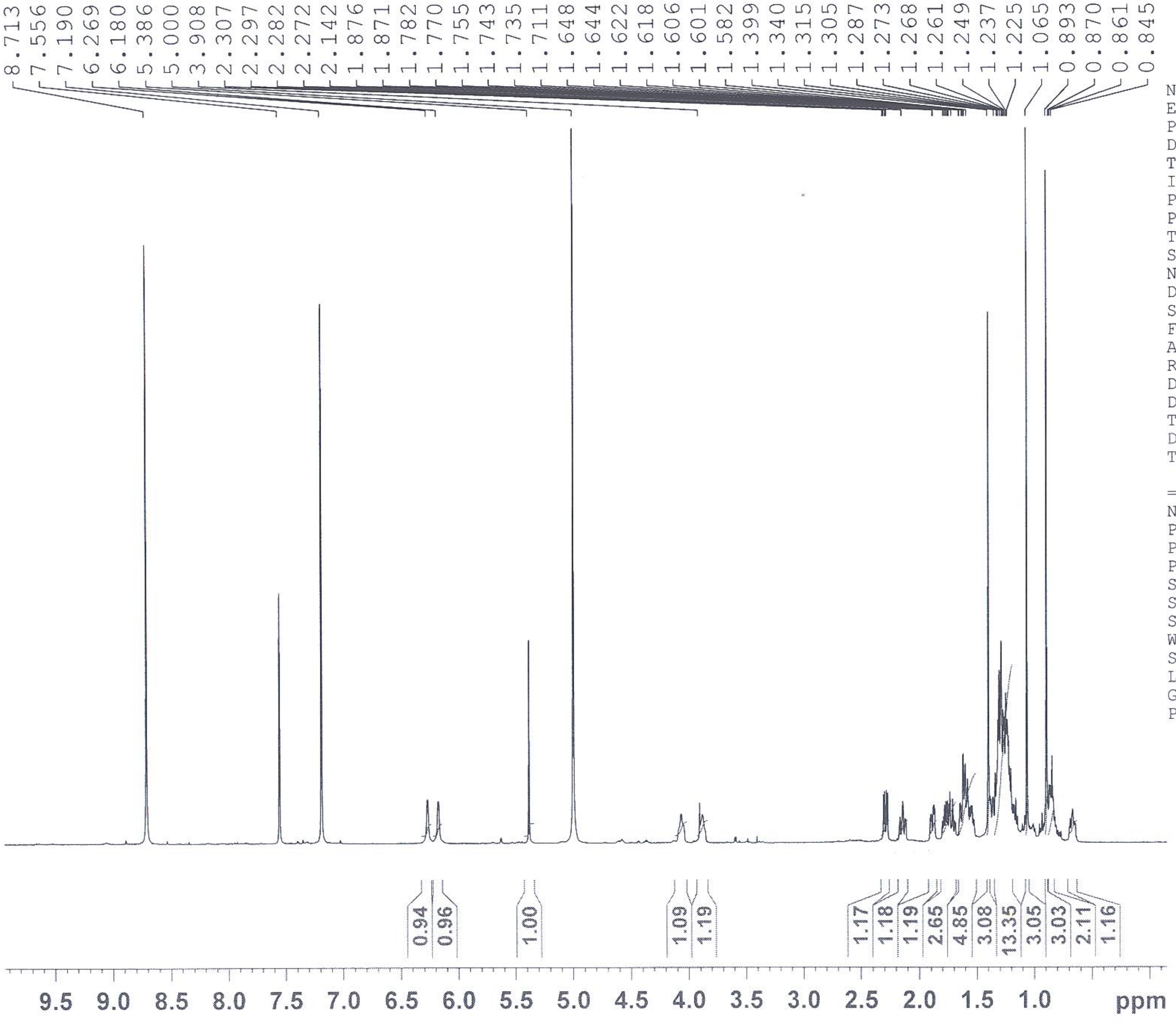

NAME	oct29-11
EXPNO	7
PROCNO	1
Date_	20111029
Time_	11.25
INSTRUM	spect
PROBHD	5 mm CPDUL 13C
PULPROG	zg30
TD	32768
SOLVENT	Pyr
NS	32
DS	0
SWH	10000.000 Hz
FIDRES	0.305176 Hz
AQ	1.6385000 sec
RG	10.1
DW	50.000 usec
DE	6.50 usec
TE	295.0 K
D1	1.00000000 sec
TD0	1

======== CHANNEL f1 ========

NUC1	1H
P1	13.50 usec
PL1	2.00 dB
PL1W	11.25274181 W
SFO1	500.3335023 MHz
SI	32768
SF	500.3299952 MHz
WDW	EM
SSB	0
LB	0.30 Hz
GB	0
PC	1.40

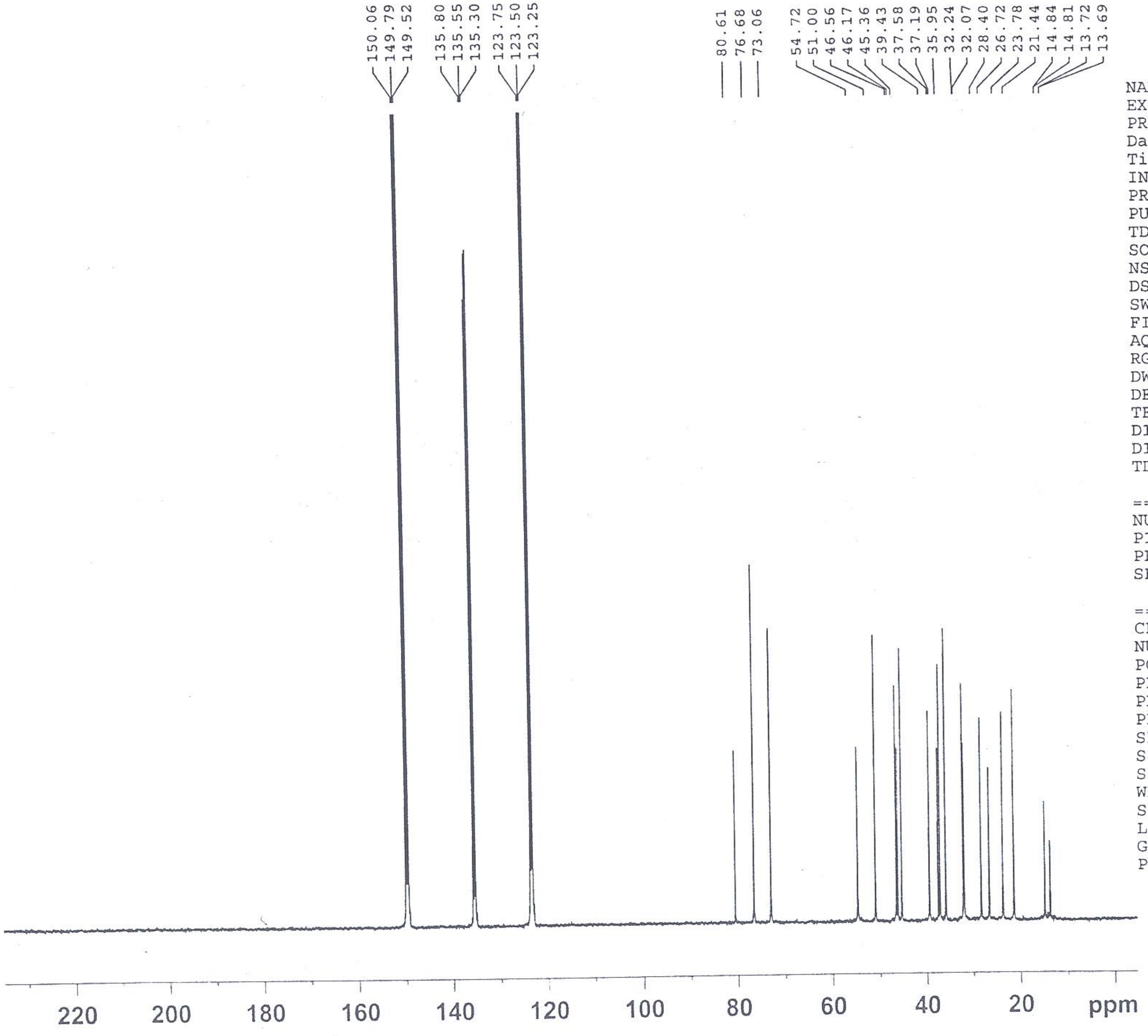

NAME	apr19	
EXPNO	1	
PROCNO	1	
Date_	20100419	
Time	13.21	
INSTRUM	spect	
PROBHD	5 mm DUL 13C-1	
PULPROG	zgpg	
TD	32768	
SOLVENT	Pyr	
NS	20480	
DS	2	
SWH	24154.590	Hz
FIDRES	0.737140	Hz
AQ	0.6783476	sec
RG	32768	
DW	20.700	usec
DE	6.50	usec
TE	300.0	K
D1	1.50000000	sec
D11	0.03000000	sec
TD0	20	
======== CHANNEL f1 ========		
NUC1	13C	
P1	10.50	usec
PL1	0.00	dB
SFO1	100.6243395	MHz
======== CHANNEL f2 ========		
CPDPRG2	waltz16	
NUC2	1H	
PCPD2	100.00	usec
PL2	-4.00	dB
PL12	15.00	dB
PL13	15.00	dB
SFO2	400.1332010	MHz
SI	32768	
SF	100.6127494	MHz
WDW	EM	
SSB	0	
LB	1.50	Hz
GB	0	
PC	1.00	

DEPT135

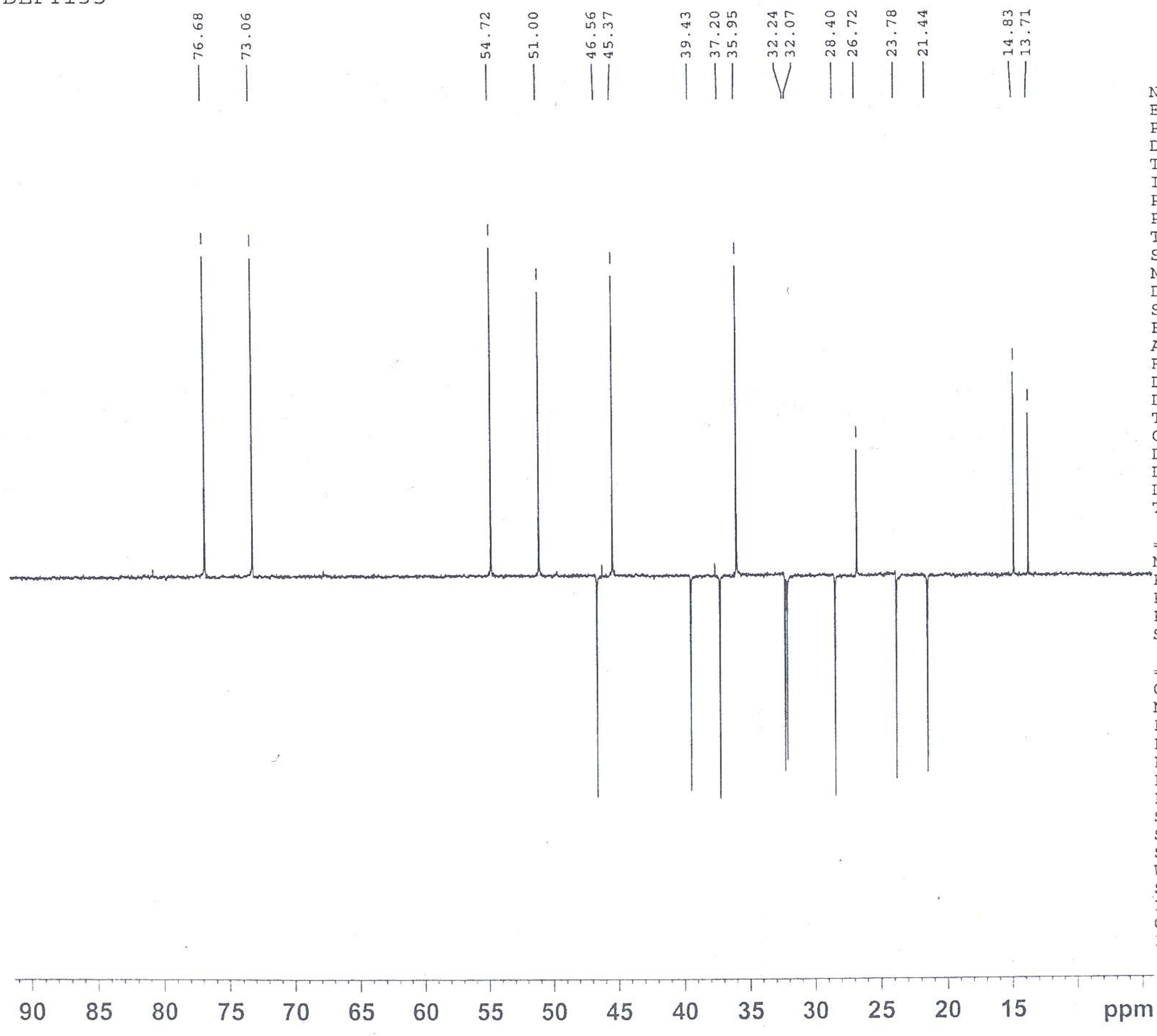

NAME	apr19
EXPNO	2
PROCNO	1
Date_	20100420
Time	2.09
INSTRUM	spect
PROBHD	5 mm DUL 13C-1
PULPROG	dept135
TD	32768
SOLVENT	Pyr
NS	10623
DS	2
SWH	19157.088 Hz
FIDRES	0.584628 Hz
AQ	0.8552948 sec
RG	32768
DW	26.100 usec
DE	6.50 usec
TE	300.0 K
CNST2	145.0000000
D1	1.50000000 sec
D2	0.00344828 sec
D12	0.00002000 sec
TD0	12
======== CHANNEL f1 ========	
NUC1	13C
P1	10.50 usec
P2	21.00 usec
PL1	0.00 dB
SFO1	100.6223272 MHz
======== CHANNEL f2 ========	
CPDPRG2	waltz16
NUC2	1H
P3	14.20 usec
P4	28.40 usec
PCPD2	100.00 usec
PL2	-4.00 dB
PL12	15.00 dB
SFO2	400.1320007 MHz
SI	32768
SF	100.6127494 MHz
WDW	EM
SSB	0
LB	1.00 Hz
GB	0
PC	1.40

LAB. No. 108

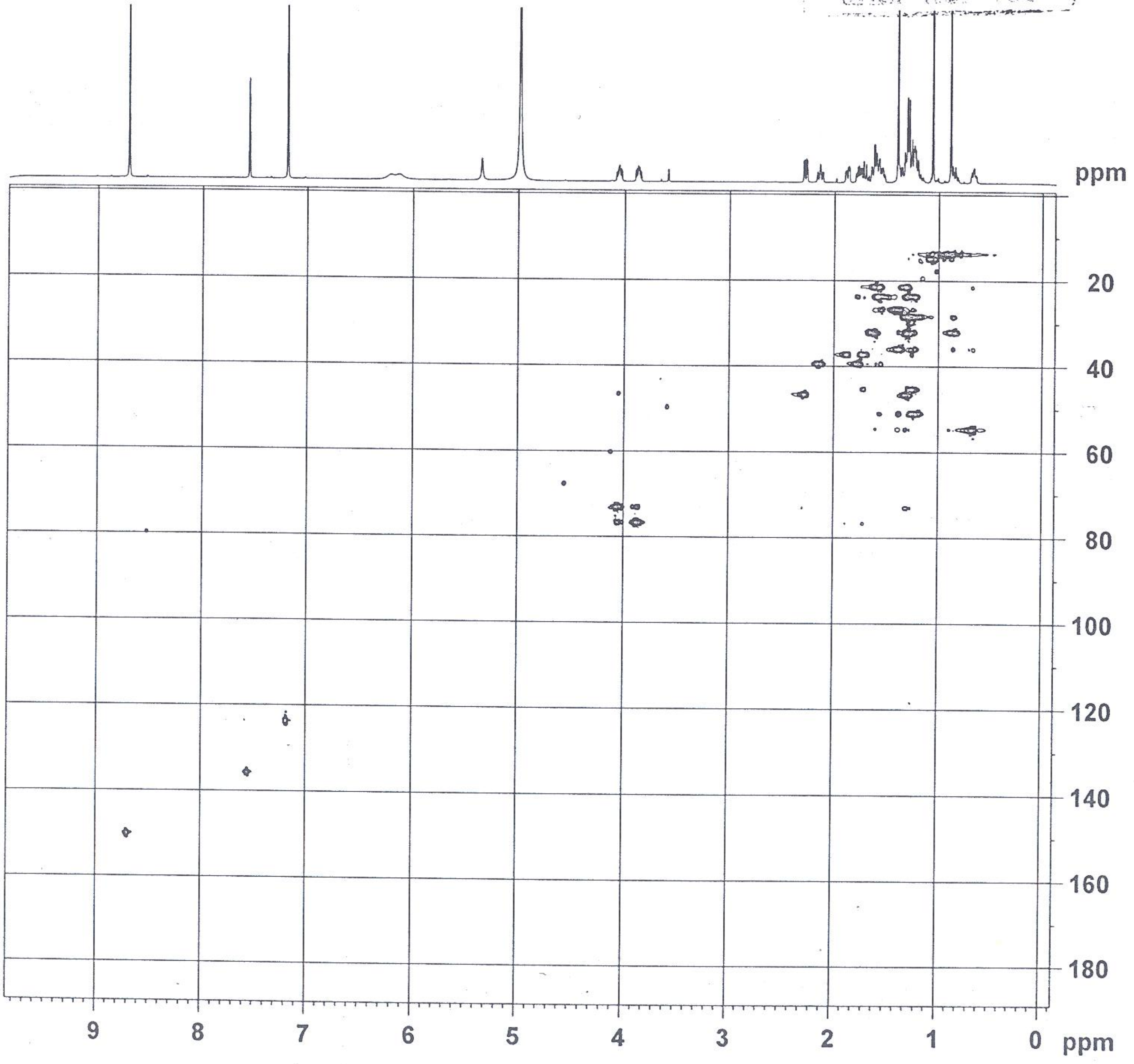

BRUKER

NAME	Feb 22
EXPNO	4
PROCNO	1
Date_	20100222
Time	12.59
INSTRUM	spect
PROBHD	5 mm PASEI 1H-
PULPROG	hsqcetgpsi
TD	1024
SOLVENT	Pyr
NS	32
DS	8
SWH	5000.000 Hz
FIDRES	4.882813 Hz
AQ	0.1025500 sec
RG	32768
DW	100.000 usec
DE	6.50 usec
TE	297.7 K
CNST2	145.0000000
D0	0.00000300 sec
D1	1.50000000 sec
D4	0.00172414 sec
D11	0.03000000 sec
D13	0.00000400 sec
D16	0.00010000 sec
D24	0.00110000 sec
IN0	0.00002090 sec
ZGOPTNS	
======== CHANNEL f1 ========	
NUC1	1H
P1	8.30 usec
P2	16.60 usec
P28	1000.00 usec
PL1	2.00 dB
PL1W	11.25274181 W
SFO1	500.3330020 MHz
======== CHANNEL f2 ========	
CPDPRG2	garp
NUC2	13C
P3	13.70 usec
P4	27.40 usec
PCPD2	80.00 usec
PL2	-1.00 dB
PL12	15.00 dB
PL2W	83.80036163 W
PL12W	2.10496974 W
SFO2	125.8200308 MHz
====== GRADIENT CHANNEL =====	
GPNAM1	SINE.100
GPNAM2	SINE.100
GPZ1	80.00 %
GPZ2	20.10 %
P16	1500.00 usec
ND0	2
TD	256
SFO1	125.82 MHz
FIDRES	93.382057 Hz
SW	190.000 ppm
FnMODE	Echo-Antiecho
SI	1024
SF	500.3305697 MHz
WDW	QSINE
SSB	2
LB	0.00 Hz
GB	0
PC	4.00
SI	1024
MC2	echo-antiecho
SF	125.8081941 MHz
WDW	QSINE
SSB	2
LB	0.00 Hz
GB	0

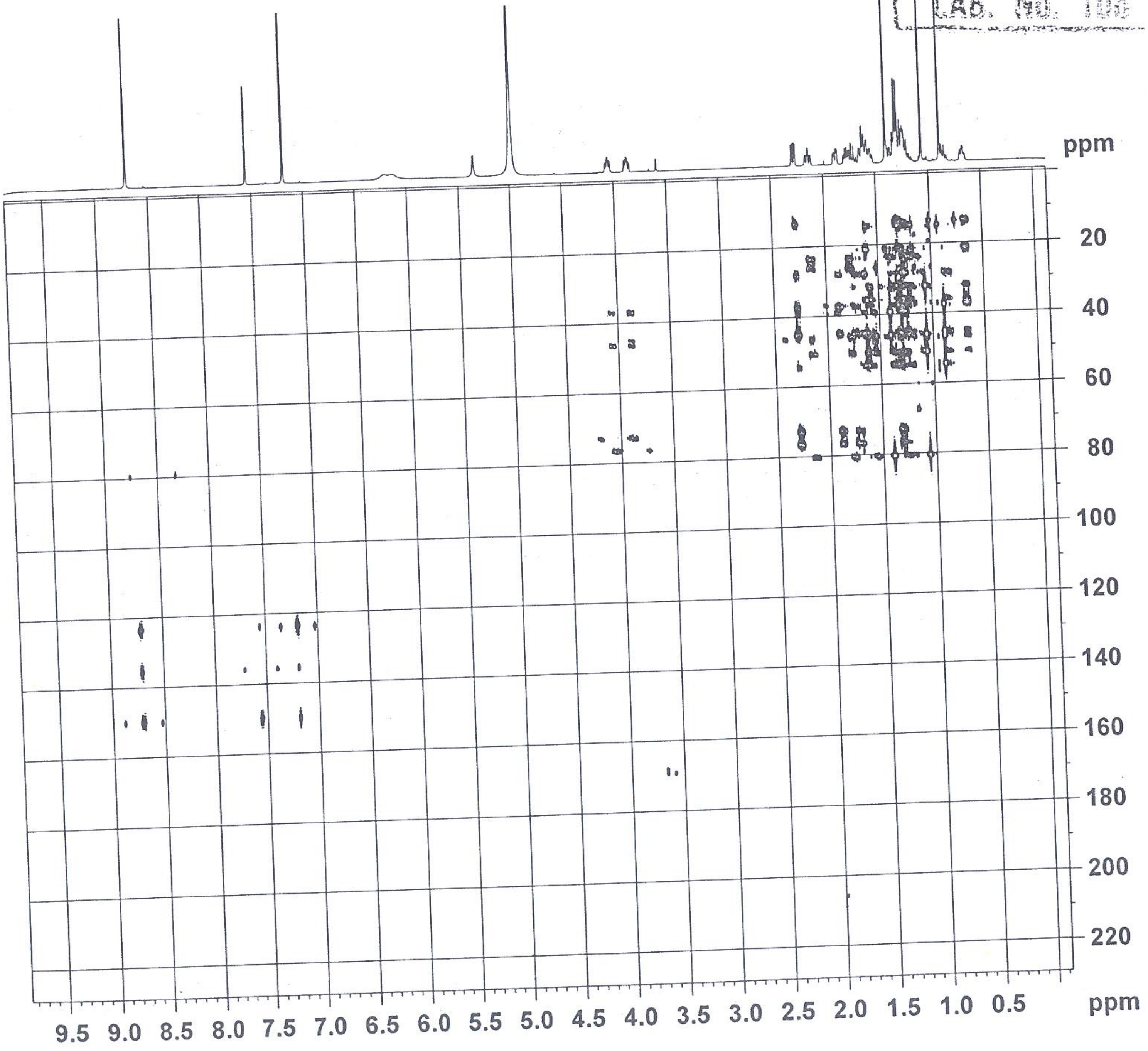

NAME	Feb 22
EXPNO	5
PROCNO	1
Date_	20100222
Time	16.43
INSTRUM	spect
PROBHD	5 mm PASEI 1H-
PULPROG	hmbcgplpndqf
TD	4096
SOLVENT	Pyr
NS	64
DS	4
SWH	5000.000 Hz
FIDRES	1.220703 Hz
AQ	0.4097500 sec
RG	23170.5
DW	100.000 usec
DE	6.50 usec
TE	296.3 K
CNST2	145.0000000
CNST13	10.0000000
D0	0.00000300 sec
D1	1.50000000 sec
D2	0.00344828 sec
D6	0.05000000 sec
D16	0.00010000 sec
IN0	0.00001730 sec

======== CHANNEL f1 ========

NUC1	1H
P1	8.30 usec
P2	16.60 usec
PL1	2.00 dB
PL1W	11.25274181 W
SFO1	500.3330020 MHz

======== CHANNEL f2 ========

NUC2	13C
P3	13.70 usec
PL2	-1.00 dB
PL2W	83.80036163 W
SFO2	125.8225469 MHz

====== GRADIENT CHANNEL =====

GPNAM1	SINE.100
GPNAM2	SINE.100
GPNAM3	SINE.100
GPZ1	50.00 %
GPZ2	30.00 %
GPZ3	40.10 %
P16	1500.00 usec
ND0	2
TD	256
SFO1	125.8225 MHz
FIDRES	113.043694 Hz
SW	230.000 ppm
FnMODE	QF
SI	2048
SF	500.3305697 MHz
WDW	SINE
SSB	0
LB	0.00 Hz
GB	0
PC	4.00
SI	1024
MC2	QF
SF	125.8081941 MHz
WDW	SINE
SSB	0
LB	0.00 Hz
GB	0

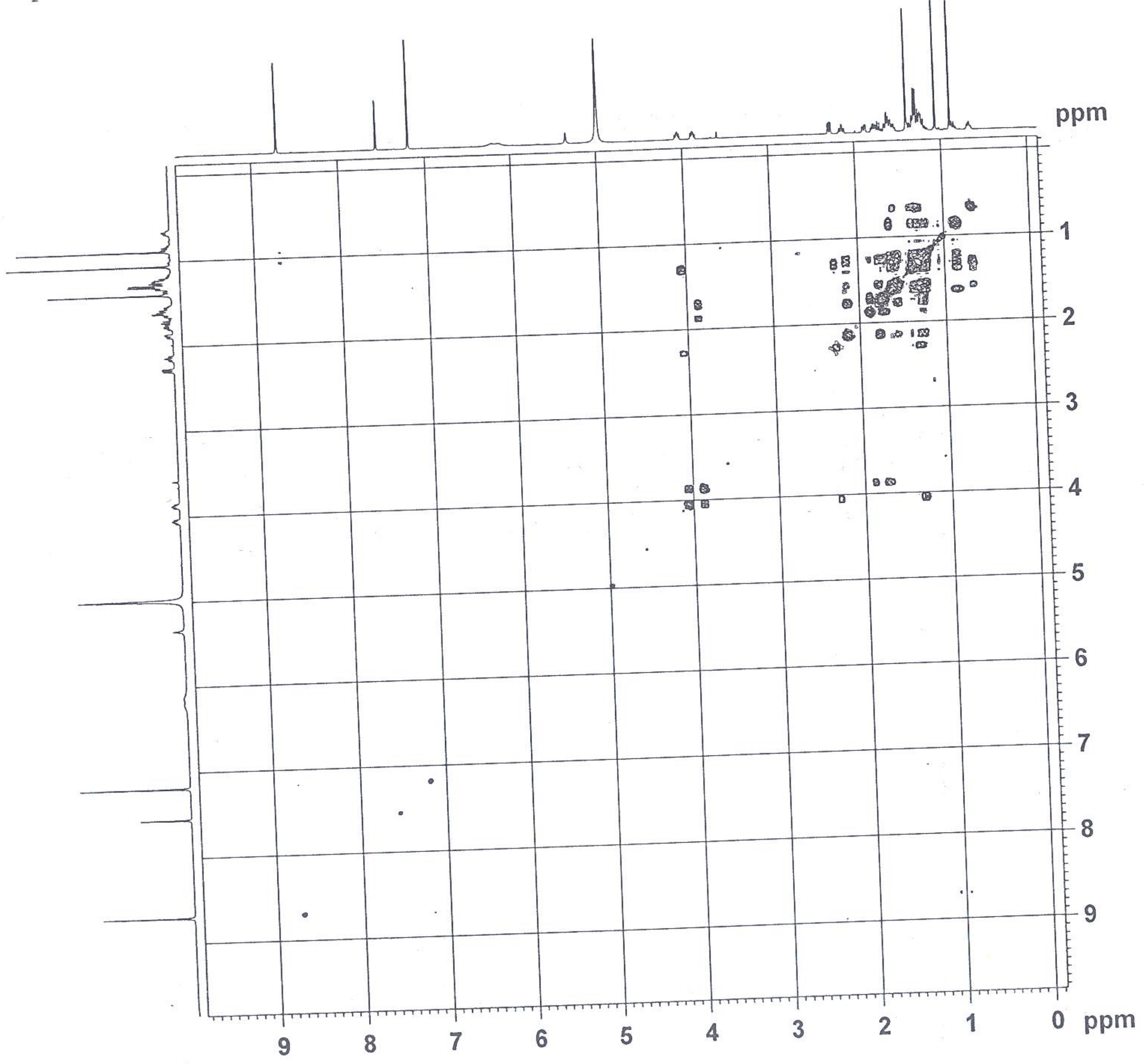

NAME	Feb 22
EXPNO	2
PROCNO	1
Date_	20100222
Time	10.16
INSTRUM	spect
PROBHD	5 mm PASEI 1H-
PULPROG	cosydfqf
TD	2048
SOLVENT	Pyr
NS	8
DS	2
SWH	5000.000 Hz
FIDRES	2.441406 Hz
AQ	0.2049500 sec
RG	45.3
DW	100.000 usec
DE	6.50 usec
TE	297.1 K
D0	0.00000300 sec
D1	1.50000000 sec
D13	0.00000400 sec
D20	0.00000400 sec
IN0	0.00020000 sec

======== CHANNEL f1 ========

NUC1	1H
P1	8.30 usec
PL1	2.00 dB
PL1W	11.25274181 W
SFO1	500.3330020 MHz
ND0	1
TD	256
SFO1	500.333 MHz
FIDRES	19.531250 Hz
SW	9.993 ppm
FnMODE	QF
SI	1024
SF	500.3305697 MHz
WDW	SINE
SSB	0
LB	0.00 Hz
GB	0
PC	4.00
SI	1024
MC2	QF
SF	500.3305697 MHz
WDW	SINE
SSB	0
LB	0.00 Hz
GB	0

[illegible] No. 108

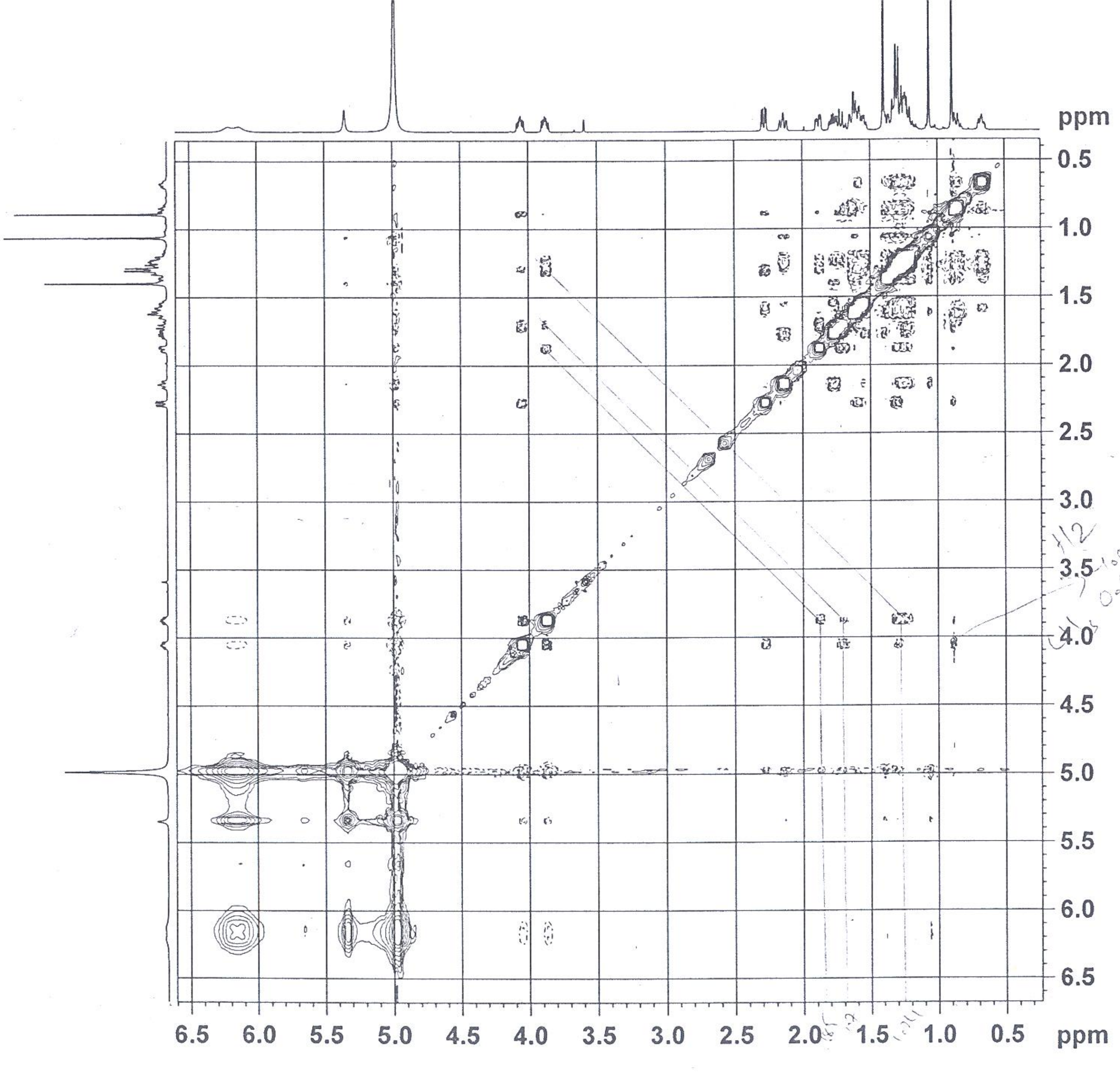

BRUKER

NAME	Feb 22
EXPNO	3
PROCNO	1
Date_	20100222
Time	11.15
INSTRUM	spect
PROBHD	5 mm PASEI 1H-
PULPROG	noesygpph
TD	2048
SOLVENT	Pyr
NS	8
DS	2
SWH	5000.000 Hz
FIDRES	2.441406 Hz
AQ	0.2049500 sec
RG	90.5
DW	100.000 usec
DE	6.50 usec
TE	297.4 K
D0	0.00008943 sec
D1	2.00000000 sec
D8	0.80000001 sec
D16	0.00010000 sec
IN0	0.00020000 sec

======== CHANNEL f1 ========

NUC1	1H
P1	8.30 usec
P2	16.60 usec
PL1	2.00 dB
PL1W	11.25274181 W
SFO1	500.3330020 MHz

====== GRADIENT CHANNEL =====

GPNAM1	SINE.100
GPNAM2	SINE.100
GPZ1	40.00 %
GPZ2	-40.00 %
P16	1500.00 usec
ND0	1
TD	256
SFO1	500.333 MHz
FIDRES	19.531250 Hz
SW	9.993 ppm
FnMODE	States-TPPI
SI	1024
SF	500.3305697 MHz
WDW	QSINE
SSB	2
LB	0.00 Hz
GB	0
PC	4.00
SI	1024
MC2	States-TPPI
SF	500.3305697 MHz
WDW	QSINE
SSB	2
LB	0.00 Hz
GB	0

File: OMC-2
Sample: MARIUM
Instrument: JEOL MSRoute
Inlet: Direct Probe

Date Run: 03-27-2010 (Time Run: 11:27:12)

Ionization mode: EI+

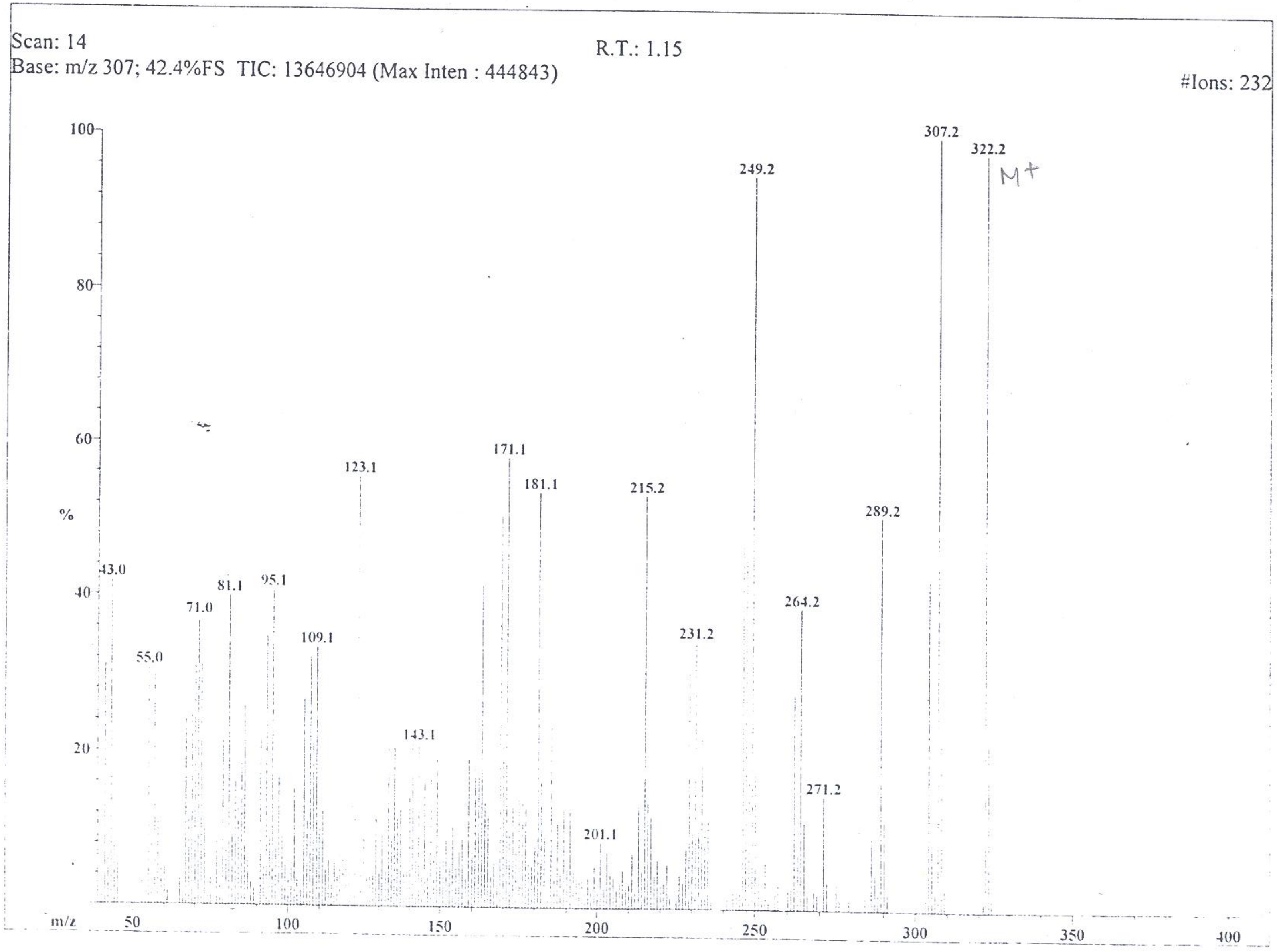

Mass	Relative Intensity	Theoretical Mass	Delta [ppm]	Delta [mmu]	RDB	Composition
264.2077	7.3429	264.2089	-4.6	-1.2	4.0	$C_{17} H_{28} O_{2}$
265.2133	2.1717	265.2168	-13.1	-3.5	3.5	$C_{17} H_{29} O_{2}$
269.1894	4.1671	269.1905	-4.4	-1.2	7.5	$C_{19} H_{25} O_{1}$
271.2066	56.2431	271.2062	1.6	0.4	6.5	$C_{19} H_{27} O_{1}$
272.2094	10.4296	272.2140	-17.0	-4.6	6.0	$C_{19} H_{28} O_{1}$
280.9819	4.5319					
286.2279	8.4857	286.2297	-6.3	-1.8	6.0	$C_{20} H_{30} O_{1}$
287.2036	2.9180	287.2011	8.5	2.4	6.5	$C_{19} H_{27} O_{2}$
289.2154	36.5854	289.2168	-4.5	-1.3	5.5	$C_{19} H_{29} O_{2}$
290.2194	7.0981	290.2246	-18.0	-5.2	5.0	$C_{19} H_{30} O_{2}$
292.9819	3.0083					
304.2376	11.3861	304.2402	-8.5	-2.6	5.0	$C_{20} H_{32} O_{2}$
305.2418	2.7627					
307.2262	18.2279	307.2273	-3.7	-1.1	4.5	$C_{19} H_{31} O_{3}$
308.2294	3.6973	308.2351	-18.7	-5.8	4.0	$C_{19} H_{32} O_{3}$
322.2507	9.7509	322.2508	-0.4	-0.1	4.0	$C_{20} H_{34} O_{3}$
330.9787	3.1591					

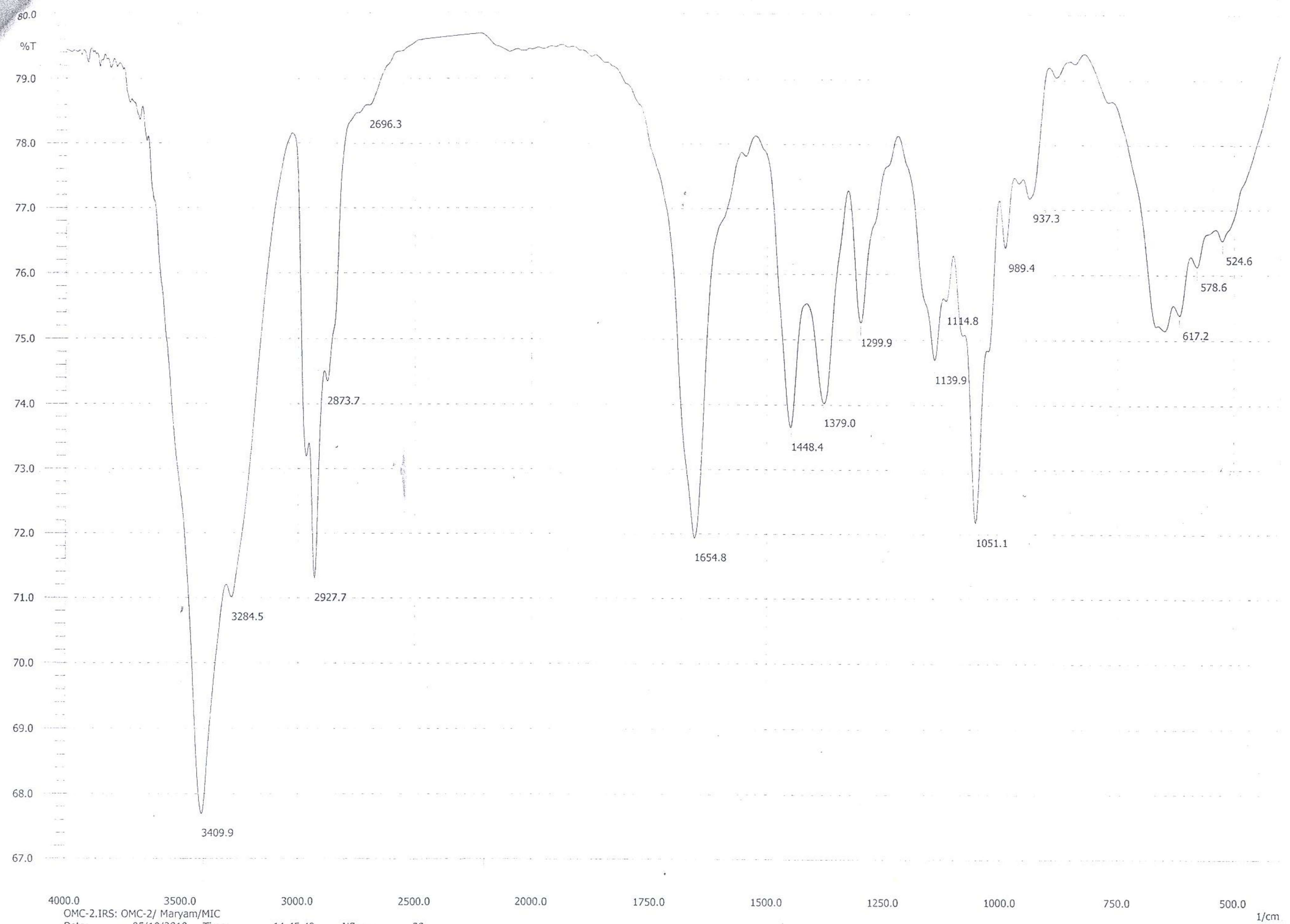

OMC-2.IRS: OMC-2/ Maryam/MIC					
Date:	05/10/2010	Time:	14:45:49	NScans:	20
Type:	HYPER IR	User:	Zubair Ahmad	Detector:	standard
Abscissa:	1/cm	Ordinate:	%T	Apodization:	Happ
Min:	401.17	Max:	3998.16	Range:	1/cm
Ndp:	1866	Data Interval:	1.92868	Resolution:	4.0
Gain:	auto	Aperture:	auto	Mirror Speed:	2.8(low)
