# Supplementary material for: Synthesis of some potent immunomodulatory and anti-inflammatory metabolites by fungal transformation of anabolic steroid oxymetholone
Source: Chem Cent J. 2012 Dec 10;6:153. doi: 10.1186/1752-153X-6-153 (PMC3740782; doi:10.1186/1752-153X-6-153)

No 108

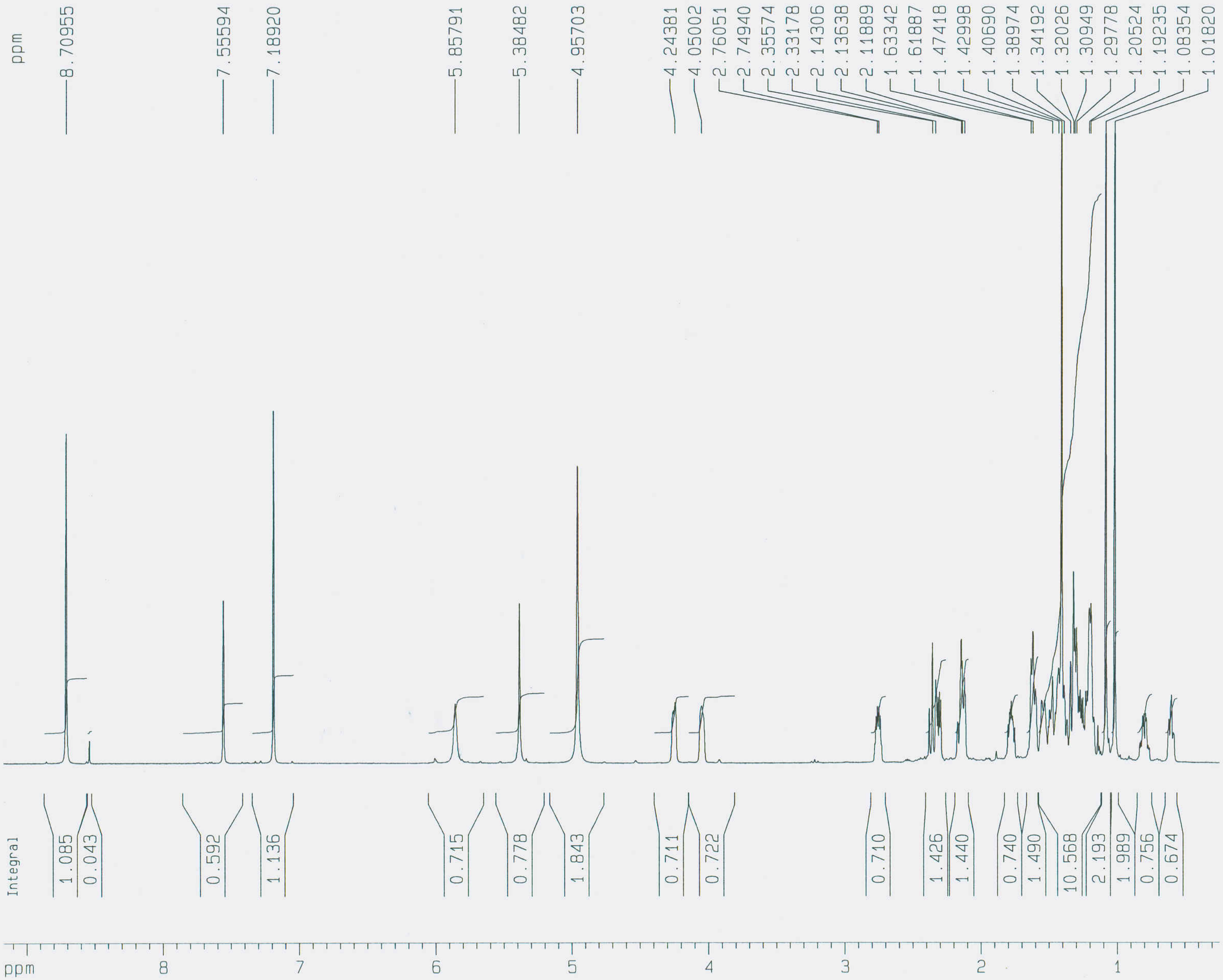

Current Data Parameters	
NAME	nov01
EXPNO	2
PROCNO	1

F2 - Acquisition Parameters	
Date_	20081101
Time	10.28
INSTRUM	spect
PROBHD	5 mm CPTCI 1H-
PULPROG	zg30
TD	32768
SOLVENT	Pyr
NS	10
DS	0
SWH	5630.631 Hz
FIDRES	0.171833 Hz
AQ	2.9099371 sec
RG	5
DW	88.800 usec
DE	6.00 usec
TE	299.3 K
D1	1.00000000 sec
MCREST	0.00000000 sec
MCWRK	0.01500000 sec

======== CHANNEL f1 ========	
NUC1	1H
P1	7.40 usec
PL1	3.30 dB
SFO1	600.2335414 MHz

F2 - Processing parameters	
SI	16384
SF	600.2306835 MHz
WDW	EM
SSB	0
LB	0.30 Hz
GB	0
PC	1.20

1D NMR plot parameters	
CX	20.00 cm
CY	12.50 cm
F1P	9.171 ppm
F1	5504.86 Hz
F2P	0.245 ppm
F2	147.05 Hz
PPMCM	0.44631 ppm/cm
HZCM	267.89035 Hz/cm

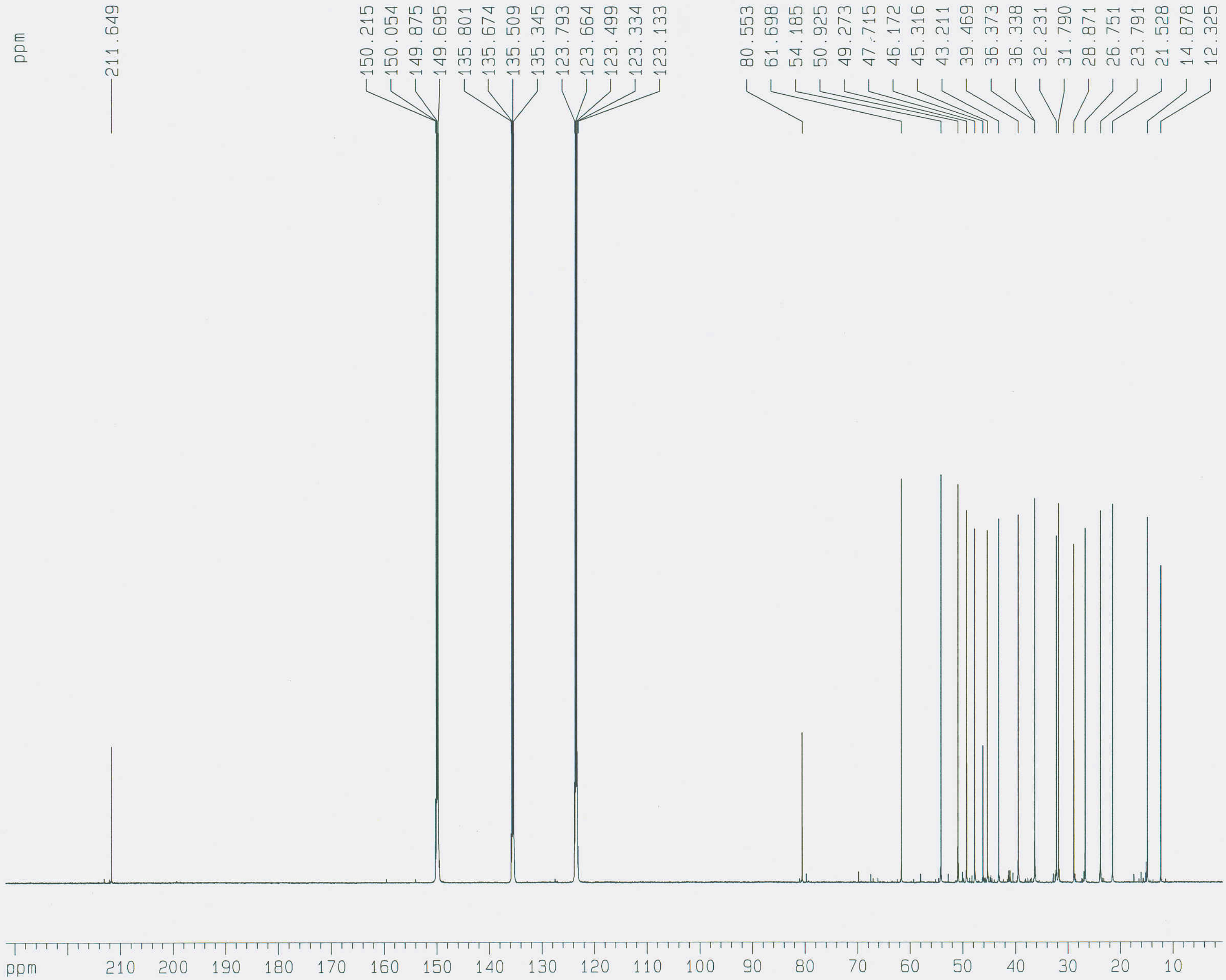

Current Data Parameters

NAME	nov01
EXPNO	7
PROCNO	1

F2 - Acquisition Parameters

Date_	20081101
Time	19.58
INSTRUM	spect
PROBHD	5 mm CPTCI 1H-
PULPROG	zgpg
TD	65536
SOLVENT	Pyr
NS	12288
DS	4
SWH	35971.223 Hz
FIDRES	0.548877 Hz
AQ	0.9110143 sec
RG	32768
DW	13.900 usec
DE	6.00 usec
TE	297.9 K
D1	1.50000000 sec
d11	0.03000000 sec
DELTA	1.39999998 sec
MCREST	0.00000000 sec
MCWRK	0.01500000 sec

======== CHANNEL f1 ========

NUC1	13C
P1	16.00 usec
PL1	2.00 dB
SFO1	150.9453107 MHz

======== CHANNEL f2 ========

CPDPRG2	waltz16
NUC2	1H
PCPD2	65.00 usec
PL2	3.30 dB
PL12	21.50 dB
PL13	27.00 dB
SFO2	600.2330011 MHz

F2 - Processing parameters

SI	32768
SF	150.9280938 MHz
WDW	EM
SSB	0
LB	1.50 Hz
GB	0
PC	1.20

1D NMR plot parameters

CX	20.00 cm
CY	45.00 cm
F1P	231.766 ppm
F1	34980.04 Hz
F2P	0.558 ppm
F2	84.24 Hz
PPMCM	11.56040 ppm/cm
HZCM	1744.78967 Hz/cm

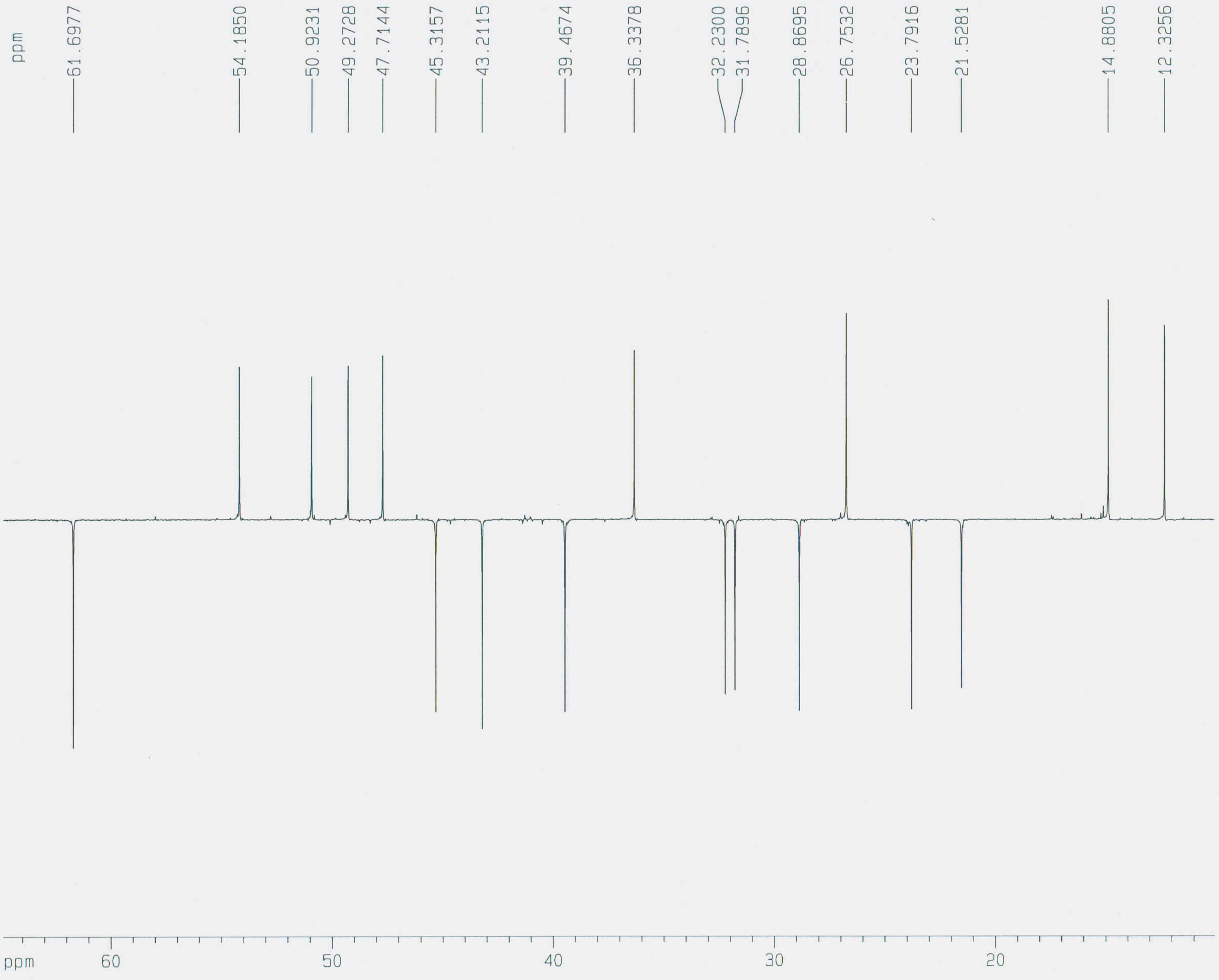

Current Data Parameters

NAME	nov01
EXPNO	8
PROCNO	1

F2 - Acquisition Parameters

Date_	20081102
Time	4.25
INSTRUM	spect
PROBHD	5 mm CPTCI 1H-
PULPROG	deptsp135
TD	65536
SOLVENT	Pyr
NS	6144
DS	4
SWH	35971.223 Hz
FIDRES	0.548877 Hz
AQ	0.9110143 sec
RG	32768
DW	13.900 usec
DE	6.00 usec
TE	297.5 K
CNST2	145.0000000
D1	1.50000000 sec
d2	0.00344828 sec
d12	0.00002000 sec
DELTA	0.00002037 sec
MCREST	0.00000000 sec
MCWRK	0.01500000 sec

======== CHANNEL f1 ========

NUC1	13C
P1	16.00 usec
P12	2000.00 usec
PL0	120.00 dB
PL1	2.00 dB
SFO1	150.9430463 MHz
SP2	1.99 dB
SPNAM2	Crp60comp.4
SPOFF2	0.00 Hz

======== CHANNEL f2 ========

CPDPRG2	waltz16
NUC2	1H
P3	7.50 usec
p4	15.00 usec
PCPD2	65.00 usec
PL2	3.30 dB
PL12	22.00 dB
SFO2	600.2336014 MHz

F2 - Processing parameters

SI	32768
SF	150.9280938 MHz
WDW	EM
SSB	0
LB	1.50 Hz
GB	0
PC	1.20

1D NMR plot parameters

CX	20.00 cm
CY	4.00 cm
F1P	64.861 ppm
F1	9789.38 Hz
F2P	10.069 ppm
F2	1519.70 Hz
PPMCM	2.73961 ppm/cm
HZCM	413.48404 Hz/cm

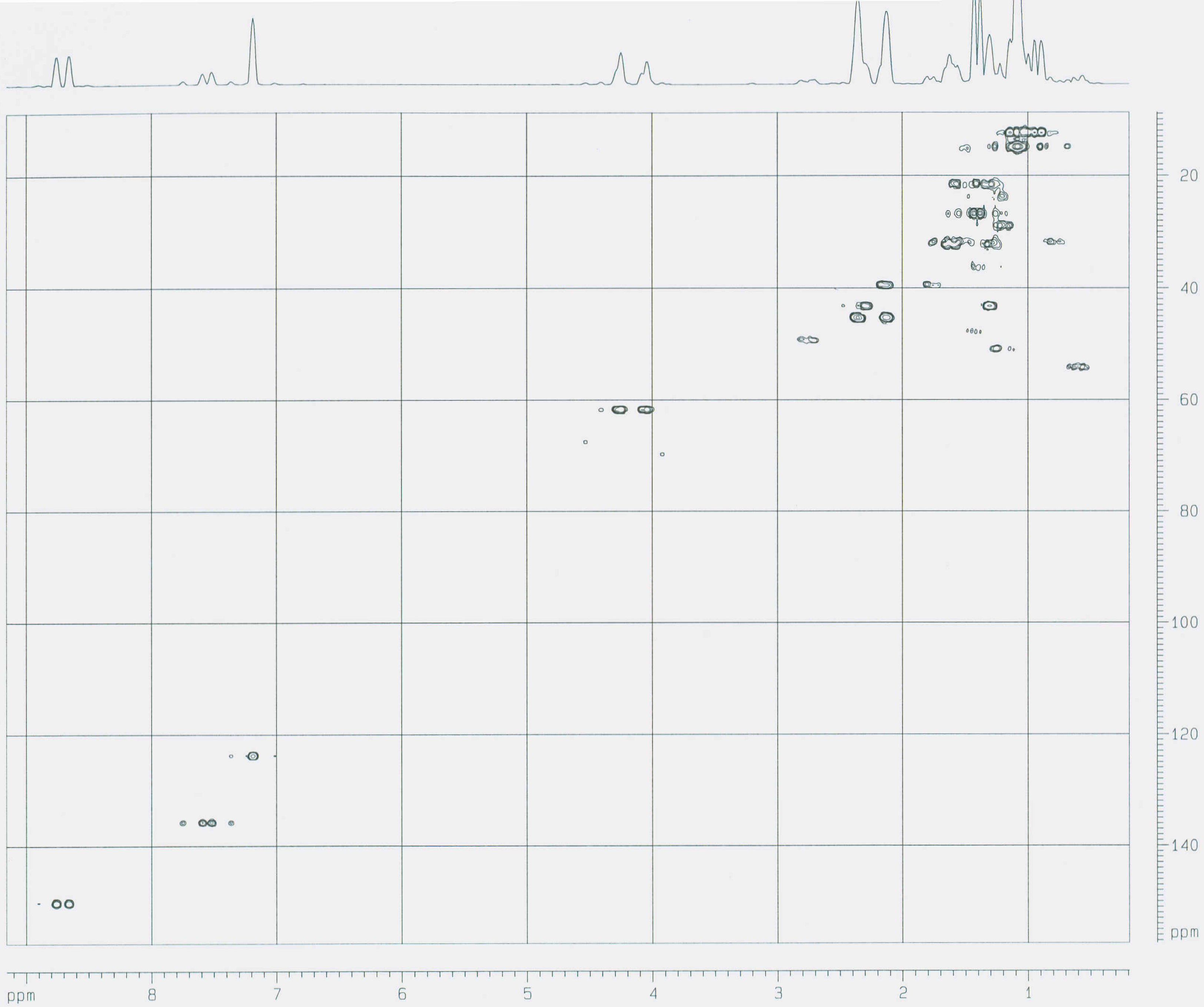

Current Data Parameters

NAME	nov01
EXPNO	5
PROCNO	1

F2 - Acquisition Parameters

Date_	20081101
Time	13.00
INSTRUM	spect
PROBHD	5 mm CPTCI 1H-
PULPROG	hmqcgpqf
TD	1024
SOLVENT	Pyr
NS	16
DS	16
SWH	5630.631 Hz
FIDRES	5.498663 Hz
AQ	0.0910700 sec
RG	29193
DW	88.800 usec
DE	6.00 usec
TE	299.0 K
CNST2	145.0000000
d0	0.00000300 sec
D1	1.50000000 sec
d2	0.00344828 sec
d12	0.00002000 sec
d13	0.00000400 sec
D16	0.00015000 sec
DELTA1	0.00127428 sec
IN0	0.00001744 sec
MCREST	0.00000000 sec
MCWRK	1.50000000 sec

======== CHANNEL f1 ========

NUC1	1H
P1	7.40 usec
p2	14.80 usec
PL1	3.30 dB
SFO1	600.2335414 MHz

======== CHANNEL f2 ========

CPDPRG2	garp
NUC2	13C
P3	16.00 usec
PCPD2	65.00 usec
PL2	2.00 dB
PL12	22.00 dB
SFO2	150.9422922 MHz

====== GRADIENT CHANNEL =====

GPNAM1	SINE.100
GPNAM2	SINE.100
GPNAM3	SINE.100
GPX1	0.00 %
GPX2	0.00 %
GPX3	0.00 %
GPY1	0.00 %
GPY2	0.00 %
GPY3	0.00 %
GPZ1	50.00 %
GPZ2	30.00 %
GPZ3	40.10 %
P16	2000.00 usec

F1 - Acquisition parameters

ND0	2
TD	256
SFO1	150.9423 MHz
FIDRES	112.007172 Hz
SW	189.966 ppm
FnMODE	QF

F2 - Processing parameters

SI	1024
SF	600.2306835 MHz
WDW	QSINE
SSB	0
LB	0.00 Hz
GB	0
PC	1.40

F1 - Processing parameters

SI	512
MC2	QF
SF	150.9280938 MHz
WDW	QSINE
SSB	0
LB	0.00 Hz
GB	0

2D NMR plot parameters

CX2	20.00 cm
CX1	15.00 cm
F2PLO	9.159 ppm
F2LO	5497.25 Hz
F2PHI	0.190 ppm
F2HI	114.05 Hz
F1PLO	157.525 ppm
F1LO	23774.98 Hz
F1PHI	8.730 ppm
F1HI	1317.54 Hz
F2PPMCM	0.44843 ppm/cm
F2HZCM	269.15958 Hz/cm
F1PPMCM	9.91971 ppm/cm
F1HZCM	1497.16248 Hz/cm

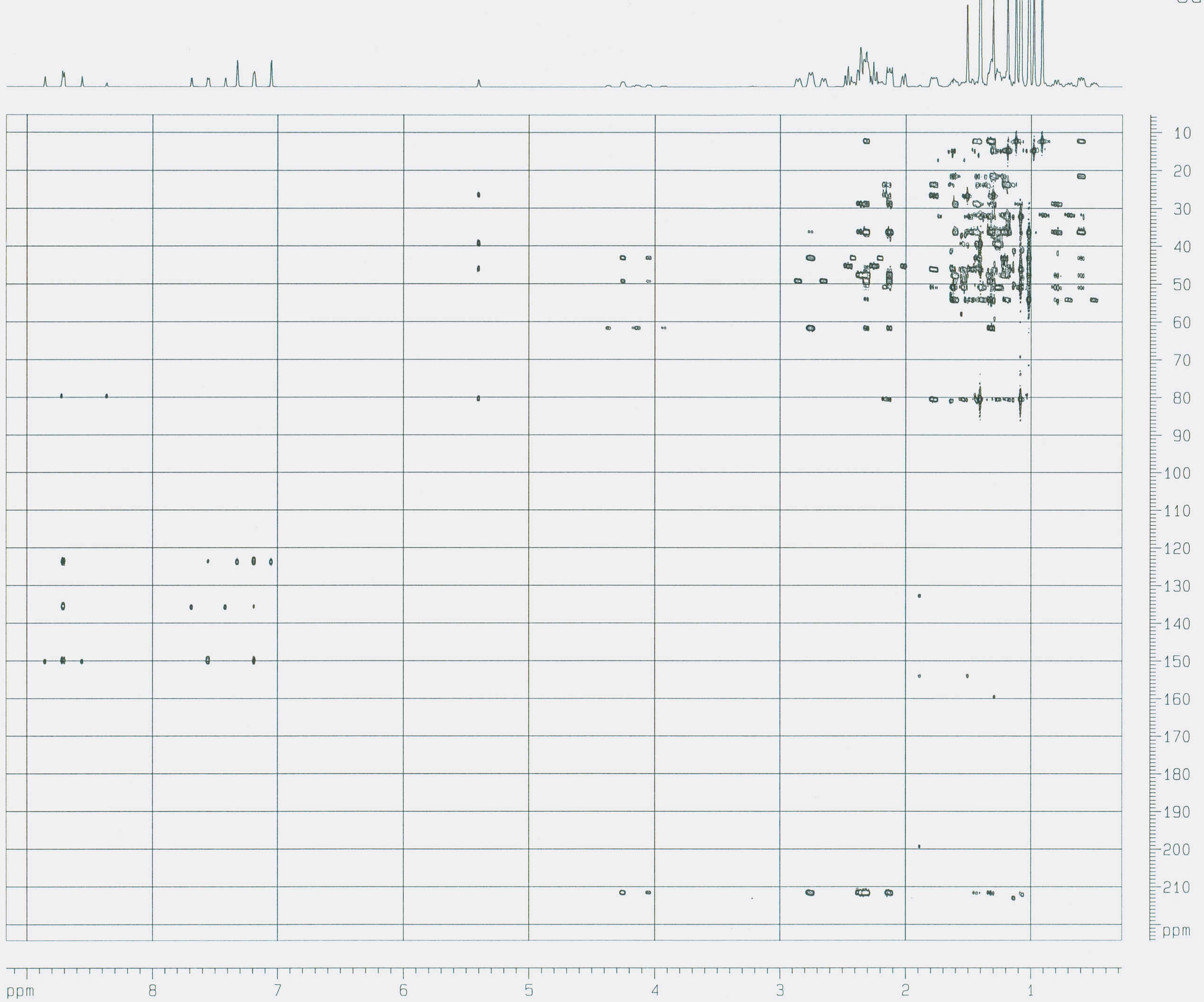

Current Data Parameters

NAME	nov01
EXPNO	6
PROCNO	1

F2 - Acquisition Parameters

Date_	20081101
Time	14.51
INSTRUM	spect
PROBHD	5 mm CPTCI 1H-
PULPROG	hmbcgpndqf
TD	4096
SOLVENT	Pyr
NS	32
DS	16
SWH	5630.631 Hz
FIDRES	1.374666 Hz
AQ	0.3638636 sec
RG	29193
DW	88.800 usec
DE	6.00 usec
TE	298.9 K
CNST13	8.0000000
d0	0.00000300 sec
D1	1.50000000 sec
d6	0.06250000 sec
D16	0.00015000 sec
IN0	0.00001427 sec
MCREST	0.00000000 sec
MCWRK	1.50000000 sec

======== CHANNEL f1 ========

NUC1	1H
P1	7.40 usec
p2	14.80 usec
PL1	3.30 dB
SFO1	600.2335414 MHz

======== CHANNEL f2 ========

NUC2	13C
P3	16.00 usec
PL2	2.00 dB
SFO2	150.9454616 MHz

====== GRADIENT CHANNEL =====

GPNAM1	SINE.100
GPNAM2	SINE.100
GPNAM3	SINE.100
GPX1	0.00 %
GPX2	0.00 %
GPX3	0.00 %
GPY1	0.00 %
GPY2	0.00 %
GPY3	0.00 %
GPZ1	50.00 %
GPZ2	30.00 %
GPZ3	40.10 %
P16	2000.00 usec

F1 - Acquisition parameters

ND0	2
TD	256
SFO1	150.9455 MHz
FIDRES	136.821365 Hz
SW	232.046 ppm
FnMODE	QF

F2 - Processing parameters

SI	2048
SF	600.2306835 MHz
WDW	QSINE
SSB	8
LB	0.00 Hz
GB	0
PC	1.40

F1 - Processing parameters

SI	1024
MC2	QF
SF	150.9280938 MHz
WDW	QSINE
SSB	8
LB	0.00 Hz
GB	0

2D NMR plot parameters

CX2	20.00 cm
CX1	15.00 cm
F2PLO	9.168 ppm
F2LO	5502.74 Hz
F2PHI	0.272 ppm
F2HI	163.54 Hz
F1PLO	224.276 ppm
F1LO	33849.57 Hz
F1PHI	5.348 ppm
F1HI	807.21 Hz
F2PPMCM	0.44476 ppm/cm
F2HZCM	266.96008 Hz/cm
F1PPMCM	14.59518 ppm/cm
F1HZCM	2202.82349 Hz/cm

[illegible]

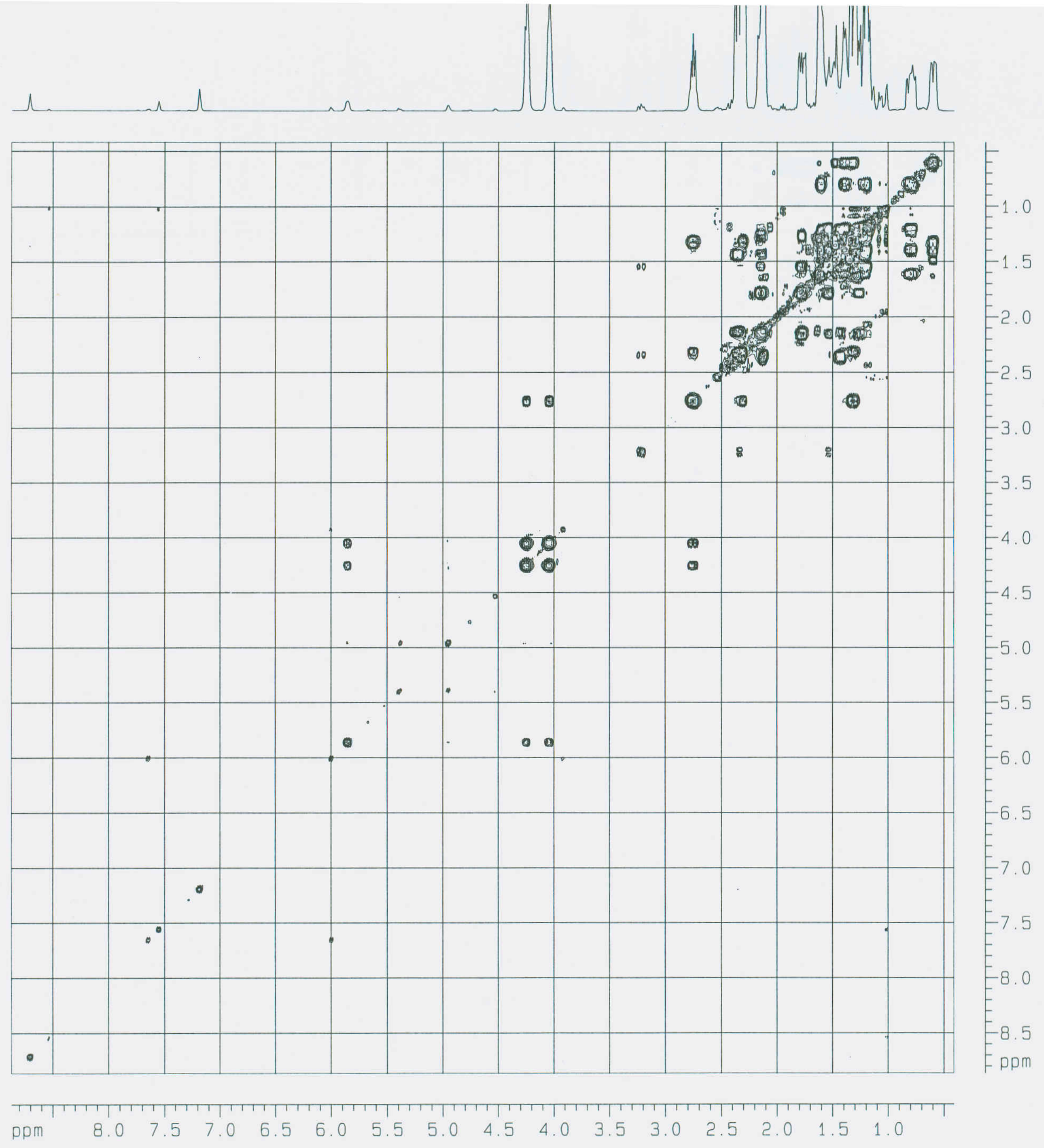

Current Data Parameters

NAME	nov01
EXPNO	3
PROCNO	1

F2 - Acquisition Parameters

Date_	20081101
Time	10.35
INSTRUM	spect
PROBHD	5 mm CPTCI 1H-
PULPROG	cosydfqf
TD	2048
SOLVENT	Pyr
NS	8
DS	4
SWH	5630.631 Hz
FIDRES	2.749331 Hz
AQ	0.1820012 sec
RG	32
DW	88.800 usec
DE	6.00 usec
TE	299.4 K
d0	0.00000300 sec
D1	1.50000000 sec
d13	0.00000400 sec
D20	0.00002000 sec
IN0	0.00017760 sec
MCREST	0.00000000 sec
MCWRK	1.50000000 sec

======== CHANNEL f1 ========

NUC1	1H
P1	7.40 usec
PL1	3.30 dB
SFO1	600.2335414 MHz

F1 - Acquisition parameters

ND0	1
TD	256
SFO1	600.2335 MHz
FIDRES	21.994652 Hz
SW	9.381 ppm
FnMODE	QF

F2 - Processing parameters

SI	1024
SF	600.2306835 MHz
WDW	SINE
SSB	0
LB	0.00 Hz
GB	0
PC	1.40

F1 - Processing parameters

SI	512
MC2	QF
SF	600.2306835 MHz
WDW	SINE
SSB	0
LB	0.00 Hz
GB	0

2D NMR plot parameters

CX2	15.00 cm
CX1	15.00 cm
F2PLO	8.875 ppm
F2LO	5326.79 Hz
F2PHI	0.410 ppm
F2HI	246.02 Hz
F1PLO	8.865 ppm
F1LO	5321.29 Hz
F1PHI	0.419 ppm
F1HI	251.52 Hz
F2PPMCM	0.56431 ppm/cm
F2HZCM	338.71765 Hz/cm
F1PPMCM	0.56309 ppm/cm
F1HZCM	337.98447 Hz/cm

[illegible]

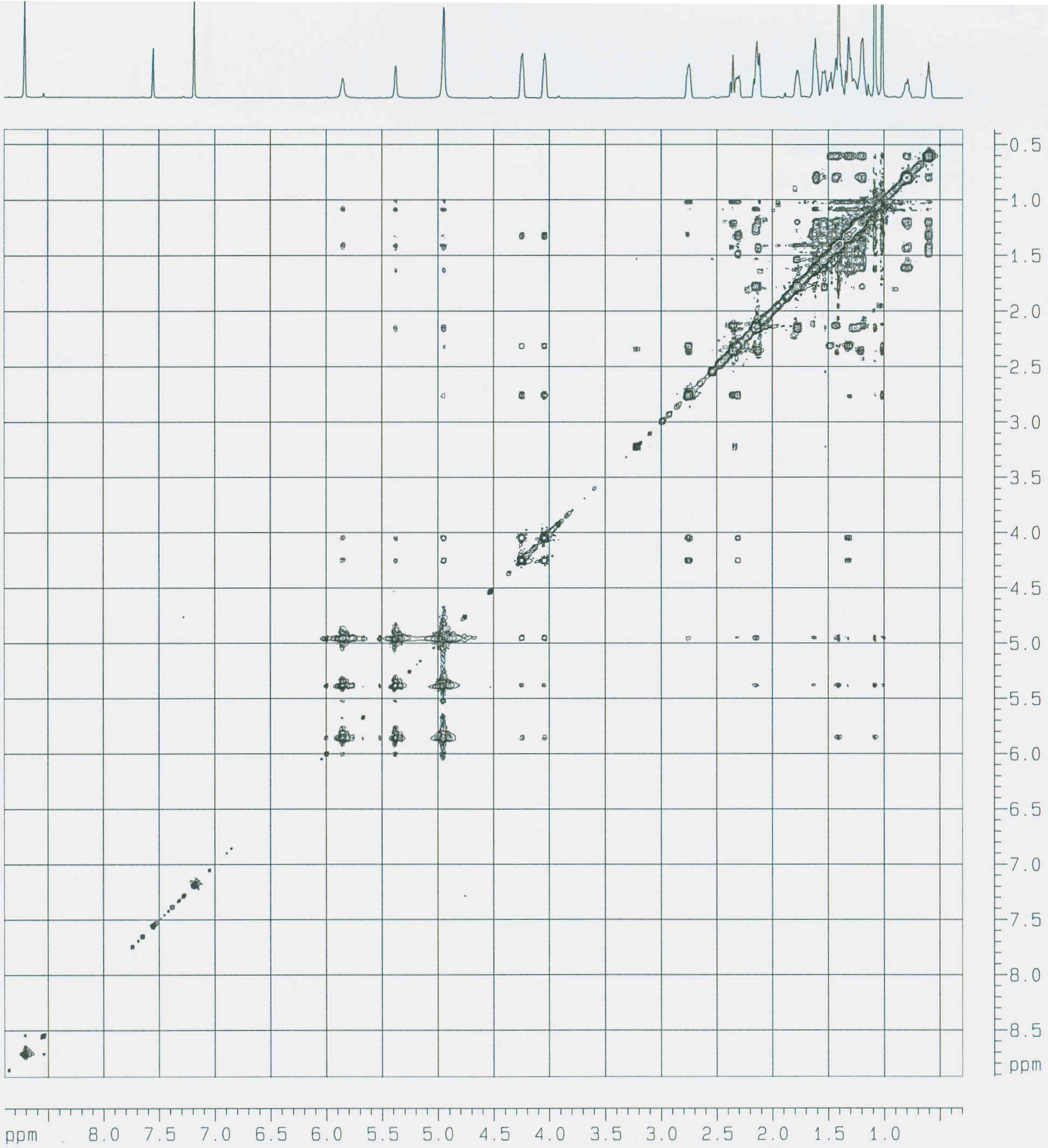

Current Data Parameters

NAME	nov01
EXPNO	4
PROCNO	1

F2 - Acquisition Parameters

Date_	20081101
Time	11.34
INSTRUM	spect
PROBHD	5 mm CPTCI 1H-
PULPROG	noesyph
TD	2048
SOLVENT	Pyr
NS	8
DS	4
SWH	5630.631 Hz
FIDRES	2.749331 Hz
AQ	0.1820012 sec
RG	20.2
DW	88.800 usec
DE	6.00 usec
TE	299.5 K
d0	0.00007938 sec
D1	1.50000000 sec
D8	0.80000001 sec
IN0	0.00017760 sec
MCREST	0.00000000 sec
MCWRK	0.75000000 sec
ST1CNT	128

======== CHANNEL f1 ========

NUC1	1H
P1	7.40 usec
PL1	3.30 dB
SFO1	600.2335414 MHz

F1 - Acquisition parameters

ND0	1
TD	256
SFO1	600.2335 MHz
FIDRES	21.994652 Hz
SW	9.381 ppm
FnMODE	States-TPPI

F2 - Processing parameters

SI	2048
SF	600.2306835 MHz
WDW	QSINE
SSB	2
LB	0.00 Hz
GB	0
PC	1.00

F1 - Processing parameters

SI	1024
MC2	States-TPPI
SF	600.2306835 MHz
WDW	QSINE
SSB	2
LB	0.00 Hz
GB	0

2D NMR plot parameters

CX2	15.00 cm
CX1	15.00 cm
F2PLO	8.893 ppm
F2LO	5337.78 Hz
F2PHI	0.295 ppm
F2HI	177.29 Hz
F1PLO	8.920 ppm
F1LO	5354.28 Hz
F1PHI	0.364 ppm
F1HI	218.53 Hz
F2PPMCM	0.57317 ppm/cm
F2HZCM	344.03302 Hz/cm
F1PPMCM	0.57042 ppm/cm
F1HZCM	342.38342 Hz/cm

File: OX-2-1HP **Date Run: 02-07-2009 (Time Run: 12:14:05)**
Sample: NAIK/H.E.J
Instrument: JEOL MSRoute
Inlet: Direct Probe **Ionization mode: EI+**

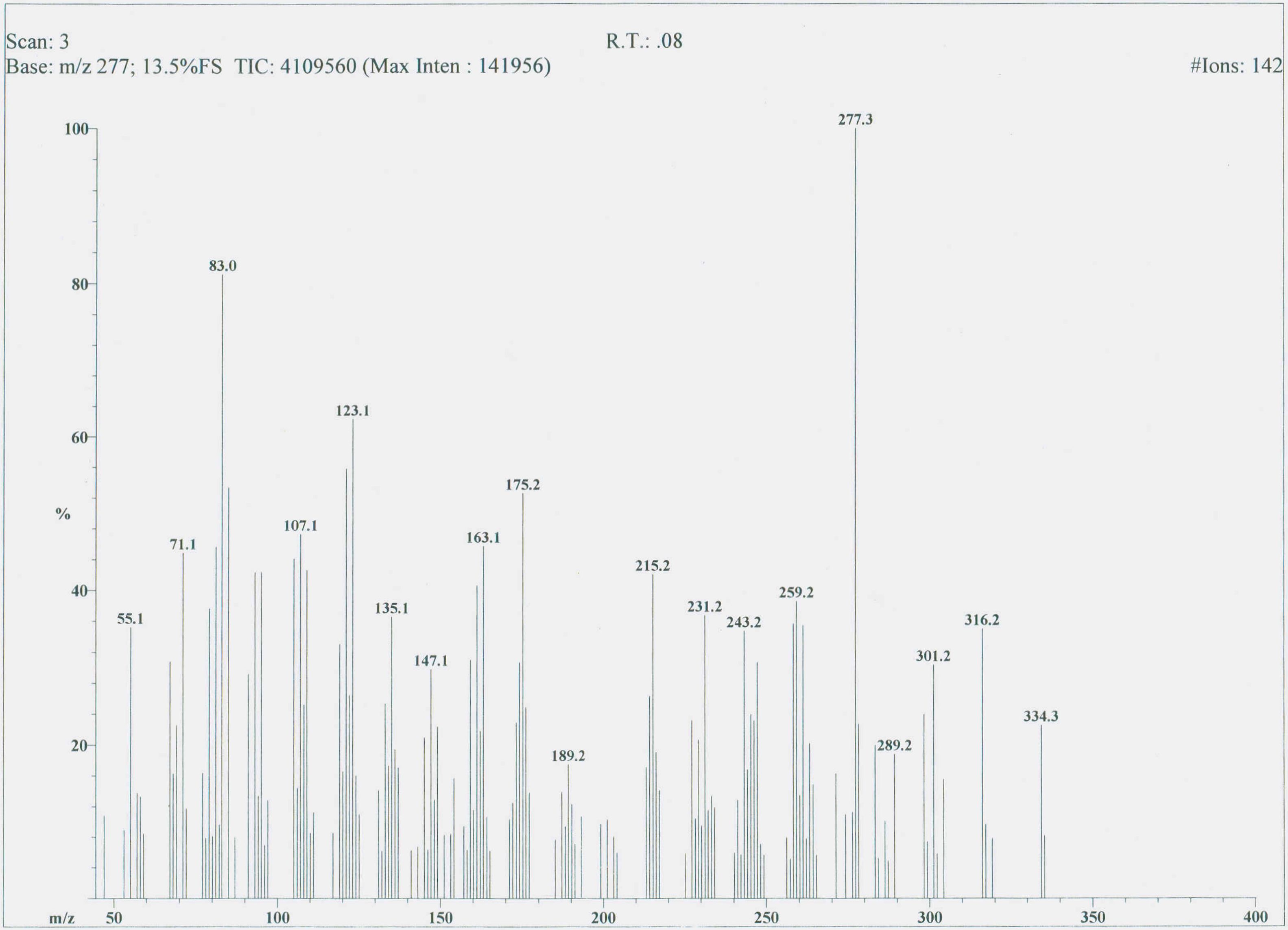

Supplement: Additional file 5 — Crystallographic information file (cif) of compound 6. [file 1752-153X-6-153-S5.pdf]
