# Supplementary material for: Synthesis of some potent immunomodulatory and anti-inflammatory metabolites by fungal transformation of anabolic steroid oxymetholone
Source: Chem Cent J. 2012 Dec 10;6:153. doi: 10.1186/1752-153X-6-153 (PMC3740782; doi:10.1186/1752-153X-6-153)

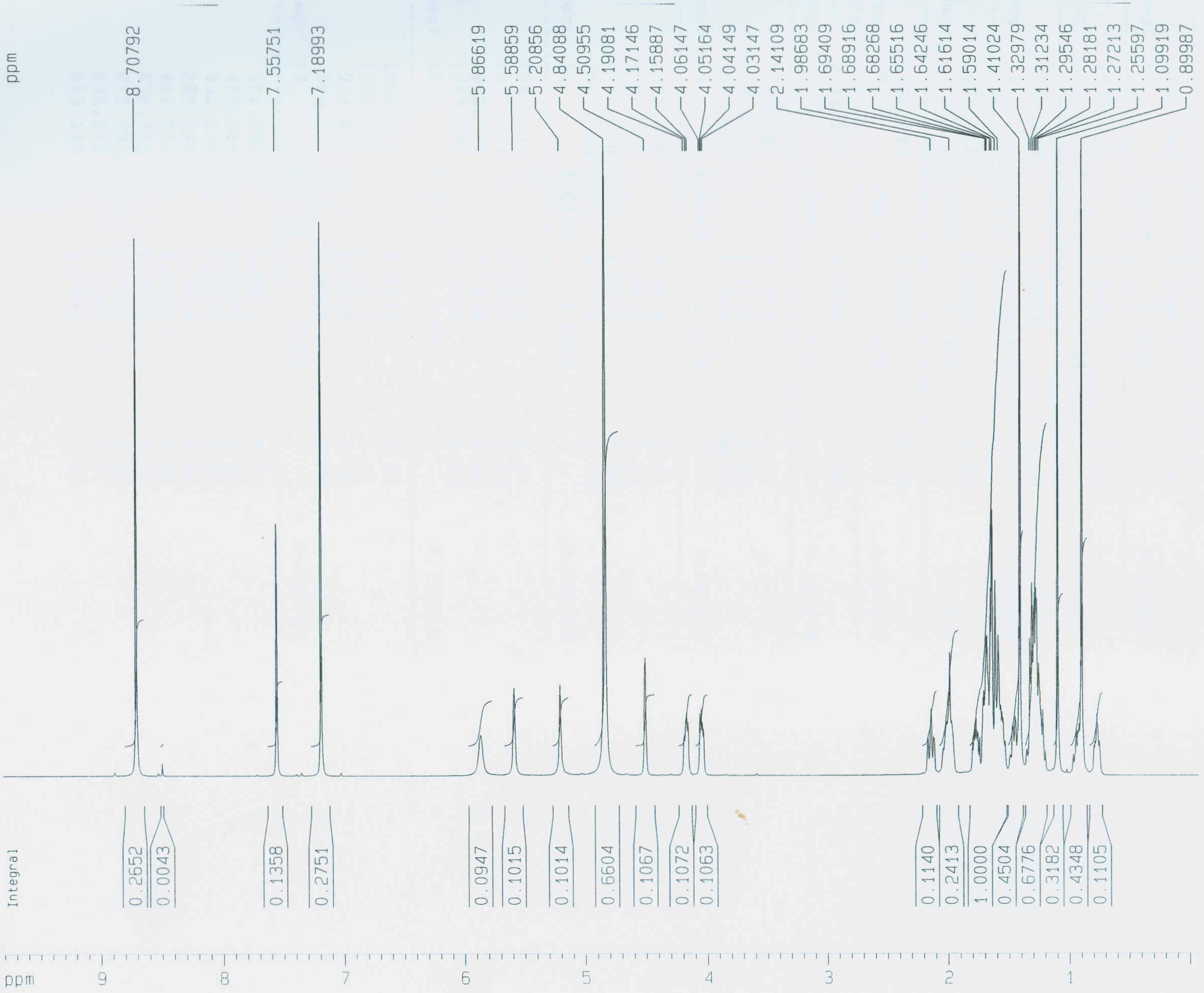

Current Data Parameters

NAME	oct31
EXPNO	1
PROCNO	1

F2 - Acquisition Parameters

Date_	20081031
Time	8.51
INSTRUM	spect
PROBHD	5 mm BBI 1H-BB
PULPROG	zg30
TD	32768
SOLVENT	Pyr
NS	64
DS	0
SWH	10000.000 Hz
FIDRES	0.305176 Hz
AQ	1.6385000 sec
RG	181
DW	50.000 usec
DE	6.00 usec
TE	304.6 K
D1	1.00000000 sec
MCREST	0.00000000 sec
MCWRK	0.01500000 sec

======== CHANNEL f1 ========

NUC1	1H
P1	7.00 usec
PL1	0.00 dB
SFO1	500.3340026 MHz

F2 - Processing parameters

SI	16384
SF	500.3307526 MHz
WDW	EM
SSB	0
LB	0.30 Hz
GB	0
PC	1.20

1D NMR plot parameters

CX	20.00 cm
CY	14.00 cm
F1P	9.812 ppm
F1	4909.28 Hz
F2P	-0.069 ppm
F2	-34.67 Hz
PPMCM	0.49407 ppm/cm
HZCM	247.19728 Hz/cm

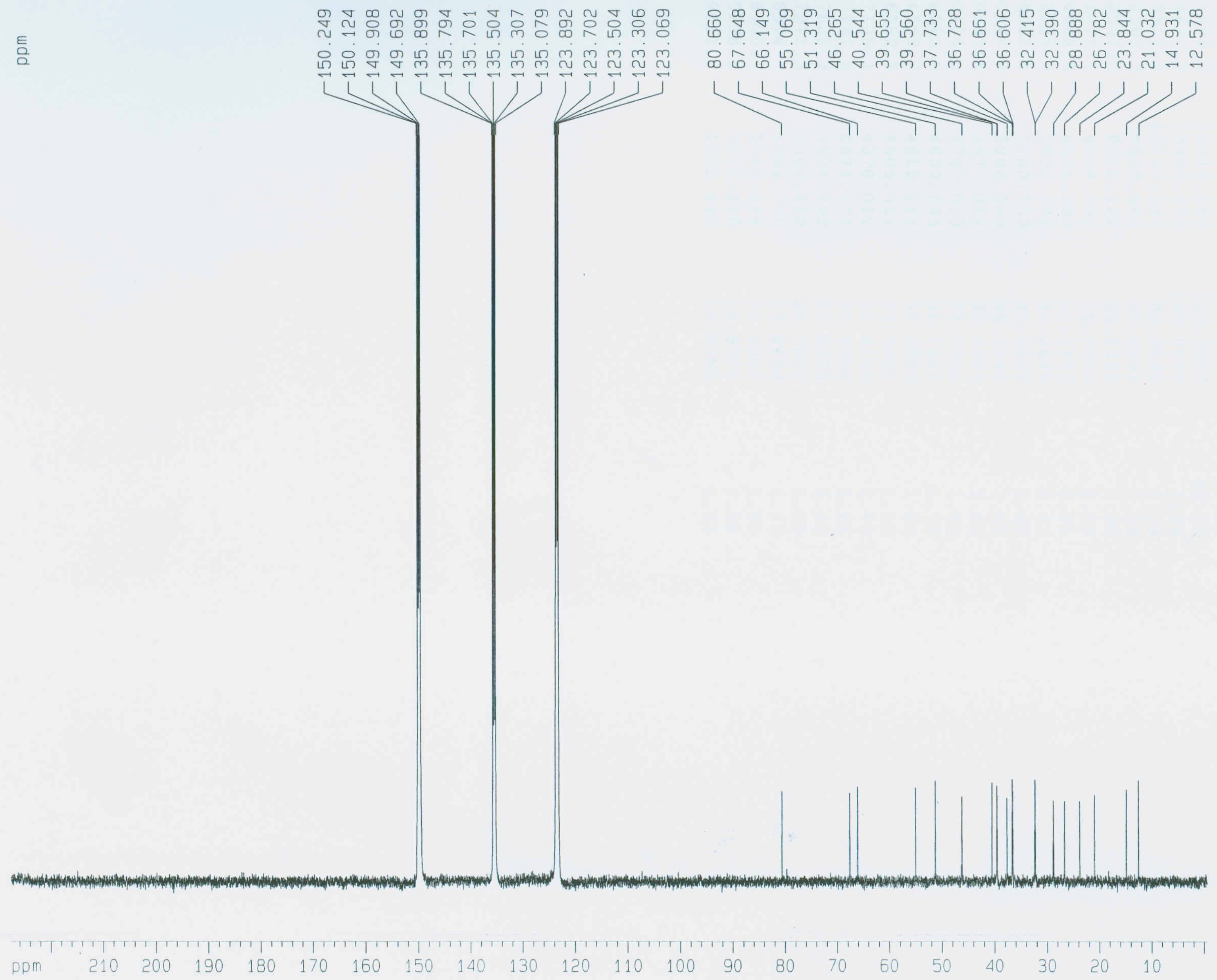

Current Data Parameters

NAME	oct31
EXPNO	6
PROCNO	1

F2 - Acquisition Parameters

Date_	20081101
Time	2.40
INSTRUM	spect
PROBHD	5 mm BBI 1H-BB
PULPROG	zgpg30
TD	65536
SOLVENT	Pyr
NS	9871
DS	4
SWH	30030.029 Hz
FIDRES	0.458222 Hz
AQ	1.0912410 sec
RG	32768
DW	16.650 usec
DE	6.00 usec
TE	305.3 K
D1	1.50000000 sec
d11	0.03000000 sec
DELTA	1.39999998 sec
MCREST	0.00000000 sec
MCWRK	0.01500000 sec

======== CHANNEL f1 ========

NUC1	13C
P1	13.50 usec
PL1	-2.00 dB
SFO1	125.8221695 MHz

======== CHANNEL f2 ========

CPDPRG2	waltz16
NUC2	1H
PCPD2	100.00 usec
PL2	0.00 dB
PL12	24.00 dB
PL13	24.00 dB
SFO2	500.3325016 MHz

F2 - Processing parameters

SI	32768
SF	125.8082360 MHz
WDW	EM
SSB	0
LB	1.50 Hz
GB	0
PC	1.20

1D NMR plot parameters

CX	20.00 cm
CY	90.00 cm
F1P	227.692 ppm
F1	28645.56 Hz
F2P	-0.568 ppm
F2	-71.50 Hz
PPMCM	11.41303 ppm/cm
HZCM	1435.85291 Hz/cm

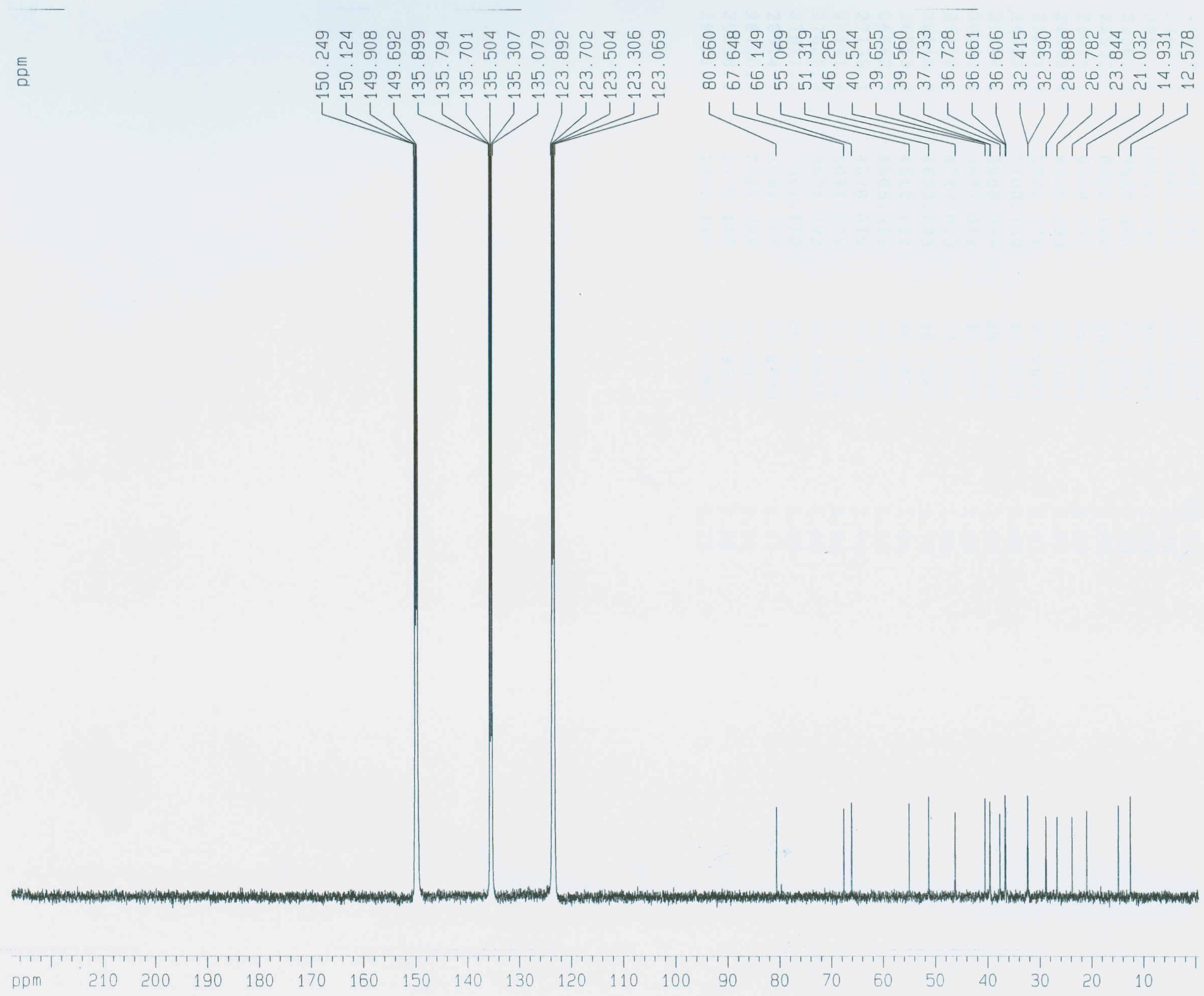

Current Data Parameters

NAME	oct31
EXPNO	6
PROCNO	1

F2 - Acquisition Parameters

Date_	20081101
Time	2.40
INSTRUM	spect
PROBHD	5 mm BBI 1H-BB
PULPROG	zgpg30
TD	65536
SOLVENT	Pyr
NS	9871
DS	4
SWH	30030.029 Hz
FIDRES	0.458222 Hz
AQ	1.0912410 sec
RG	32768
DW	16.650 usec
DE	6.00 usec
TE	305.3 K
D1	1.50000000 sec
d11	0.03000000 sec
DELTA	1.39999998 sec
MCREST	0.00000000 sec
MCWRK	0.01500000 sec

======== CHANNEL f1 ========

NUC1	13C
P1	13.50 usec
PL1	-2.00 dB
SFO1	125.8221695 MHz

======== CHANNEL f2 ========

CPDPRG2	waltz16
NUC2	1H
PCPD2	100.00 usec
PL2	0.00 dB
PL12	24.00 dB
PL13	24.00 dB
SFO2	500.3325016 MHz

F2 - Processing parameters

SI	32768
SF	125.8082360 MHz
WDW	EM
SSB	0
LB	1.50 Hz
GB	0
PC	1.20

1D NMR plot parameters

CX	20.00 cm
CY	90.00 cm
F1P	227.692 ppm
F1	28645.56 Hz
F2P	-0.568 ppm
F2	-71.50 Hz
PPMCM	11.41303 ppm/cm
HZCM	1435.85291 Hz/cm

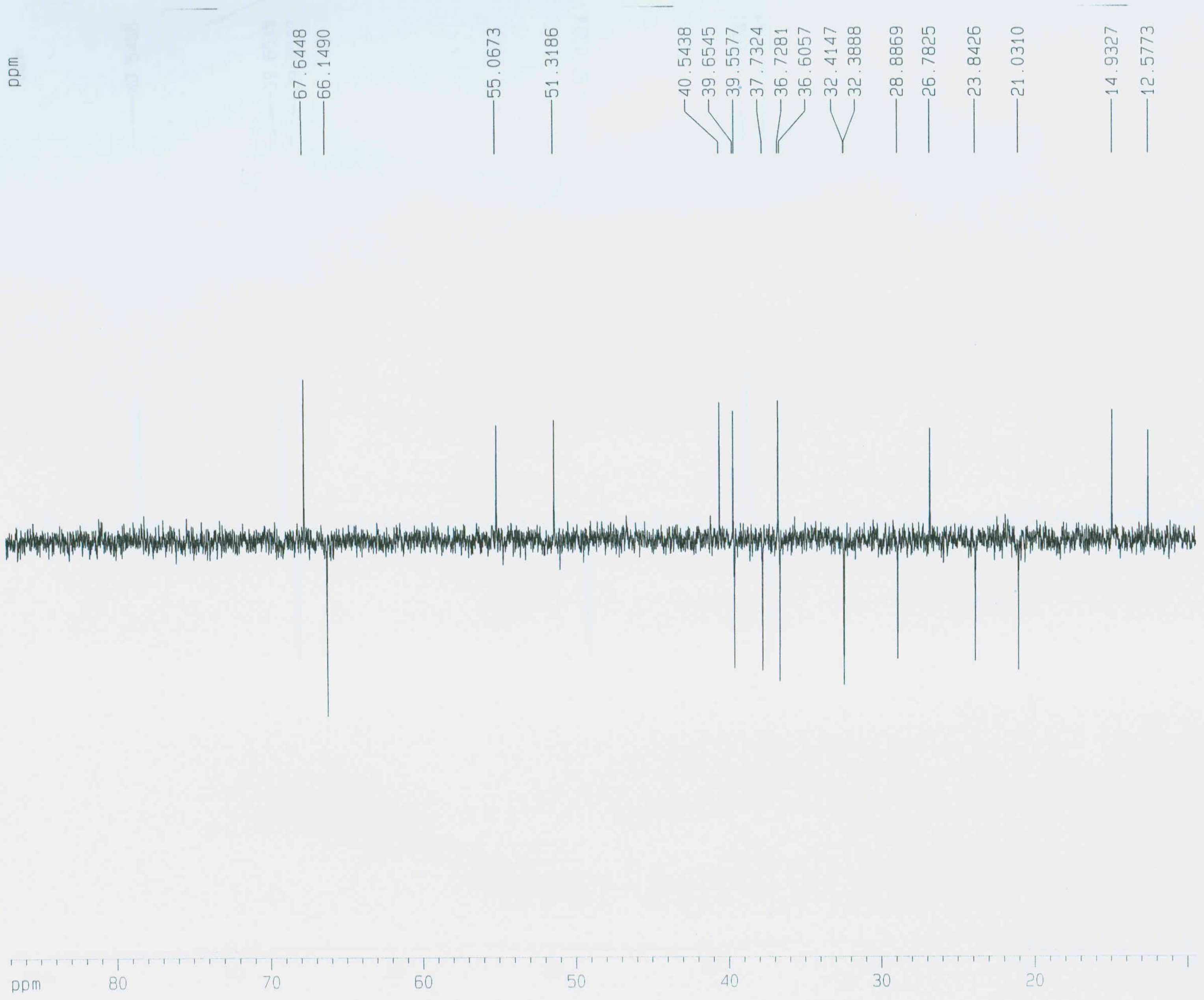

Current Data Parameters

NAME	oct31
EXPNO	7
PROCNO	1

F2 - Acquisition Parameters

Date_	20081101
Time	9.30
INSTRUM	spect
PROBHD	5 mm BBI 1H-BB
PULPROG	deptsp135
TD	65536
SOLVENT	Pyr
NS	919
DS	4
SWH	30030.029 Hz
FIDRES	0.458222 Hz
AQ	1.0912410 sec
RG	32768
DW	16.650 usec
DE	6.00 usec
TE	305.4 K
CNST2	145.0000000
D1	1.50000000 sec
d2	0.00344828 sec
d12	0.00002000 sec
DELTA	0.00001719 sec
MCREST	0.00000000 sec
MCWRK	0.01500000 sec

======== CHANNEL f1 ========

NUC1	13C
P1	13.50 usec
P12	2000.00 usec
PL0	120.00 dB
PL1	-2.00 dB
SFO1	125.8206594 MHz
SP2	1.99 dB
SPNAM2	Crp60comp.4
SPOFF2	0.00 Hz

======== CHANNEL f2 ========

CPDPRG2	waltz16
NUC2	1H
P3	7.00 usec
p4	14.00 usec
PCPD2	100.00 usec
PL2	0.00 dB
PL12	24.00 dB
SFO2	500.3330020 MHz

F2 - Processing parameters

SI	32768
SF	125.8082360 MHz
WDW	EM
SSB	0
LB	1.50 Hz
GB	0
PC	1.20

1D NMR plot parameters

CX	20.00 cm
CY	3.00 cm
F1P	87.039 ppm
F1	10950.23 Hz
F2P	9.463 ppm
F2	1190.48 Hz
PPMCM	3.87882 ppm/cm
HZCM	487.98740 Hz/cm

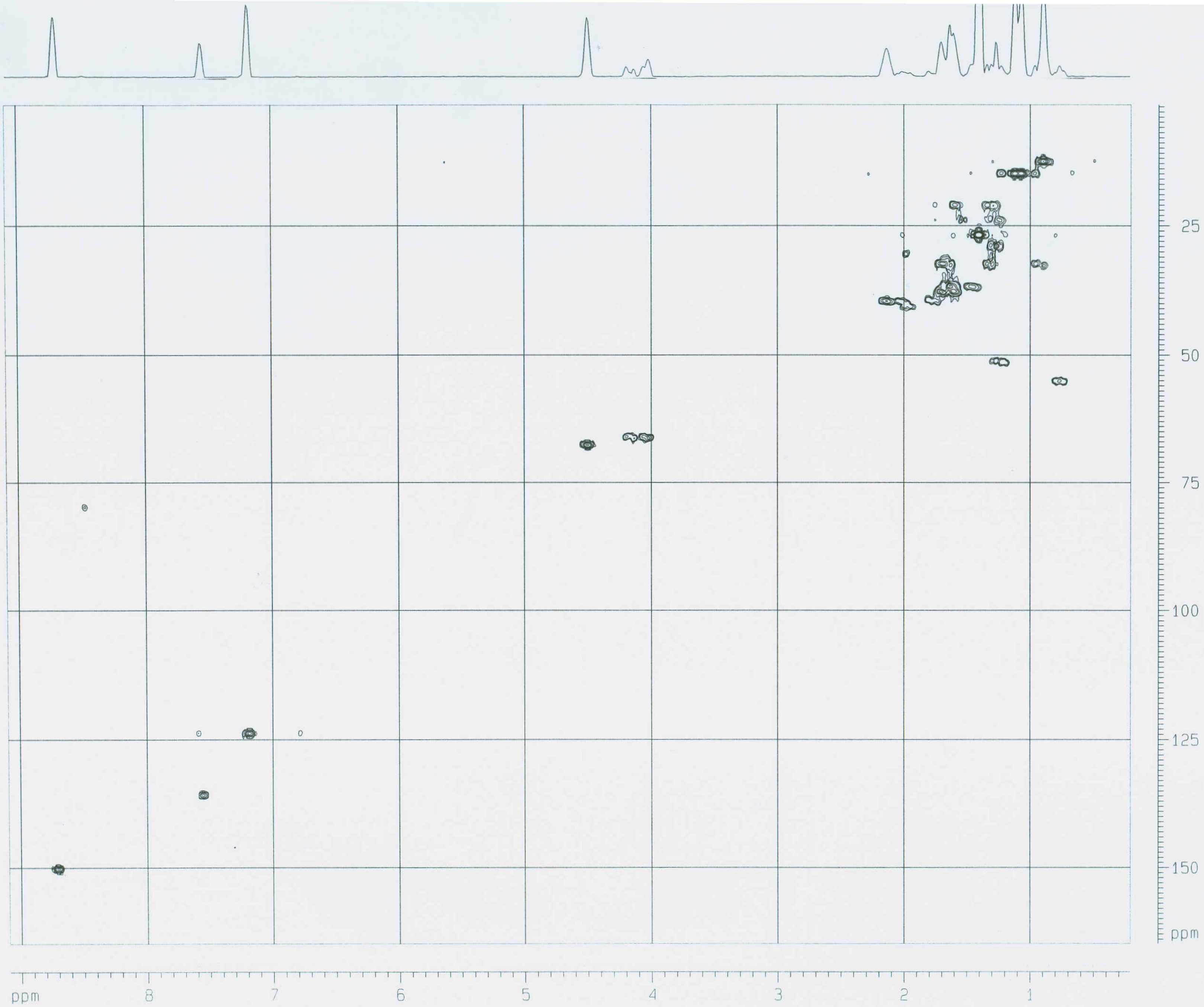

Current Data Parameters

NAME	oct31
EXPNO	4
PROCNO	1

F2 - Acquisition Parameters

Date_	20081031
Time	12.58
INSTRUM	spect
PROBHD	5 mm BBI 1H-BB
PULPROG	hmqcgpqf
TD	1024
SOLVENT	Pyr
NS	32
DS	16
SWH	4496.403 Hz
FIDRES	4.391018 Hz
AQ	0.1140300 sec
RG	26008
DW	111.200 usec
DE	6.00 usec
TE	306.2 K
CNST2	145.0000000
d0	0.00000300 sec
D1	1.50000000 sec
d2	0.00344828 sec
d12	0.00002000 sec
d13	0.00000400 sec
D16	0.00010000 sec
DELTA1	0.00232428 sec
IN0	0.00002208 sec
MCREST	0.00000000 sec
MCWRK	1.50000000 sec

======== CHANNEL f1 ========

NUC1	1H
P1	7.00 usec
p2	14.00 usec
PL1	0.00 dB
SFO1	500.3331020 MHz

======== CHANNEL f2 ========

CPDPRG2	garp
NUC2	13C
P3	13.50 usec
PCPD2	78.00 usec
PL2	-2.00 dB
PL12	13.00 dB
SFO2	125.8194017 MHz

====== GRADIENT CHANNEL =====

GPNAM1	SINE.100
GPNAM2	SINE.100
GPNAM3	SINE.100
GPX1	0.00 %
GPX2	0.00 %
GPX3	0.00 %
GPY1	0.00 %
GPY2	0.00 %
GPY3	0.00 %
GPZ1	50.00 %
GPZ2	30.00 %
GPZ3	40.10 %
P16	1000.00 usec

F1 - Acquisition parameters

ND0	2
TD	256
SFO1	125.8194 MHz
FIDRES	88.476784 Hz
SW	180.020 ppm
FnMODE	QF

F2 - Processing parameters

SI	1024
SF	500.3307525 MHz
WDW	QSINE
SSB	0
LB	0.00 Hz
GB	0
PC	1.40

F1 - Processing parameters

SI	1024
MC2	QF
SF	125.8082360 MHz
WDW	QSINE
SSB	0
LB	0.00 Hz
GB	0

2D NMR plot parameters

CX2	20.00 cm
CX1	15.00 cm
F2PLO	9.093 ppm
F2LO	4549.40 Hz
F2PHI	0.211 ppm
F2HI	105.69 Hz
F1PLO	164.881 ppm
F1LO	20743.37 Hz
F1PHI	1.547 ppm
F1HI	194.64 Hz
F2PPMCM	0.44408 ppm/cm
F2HZCM	222.18553 Hz/cm
F1PPMCM	10.88892 ppm/cm
F1HZCM	1369.91553 Hz/cm

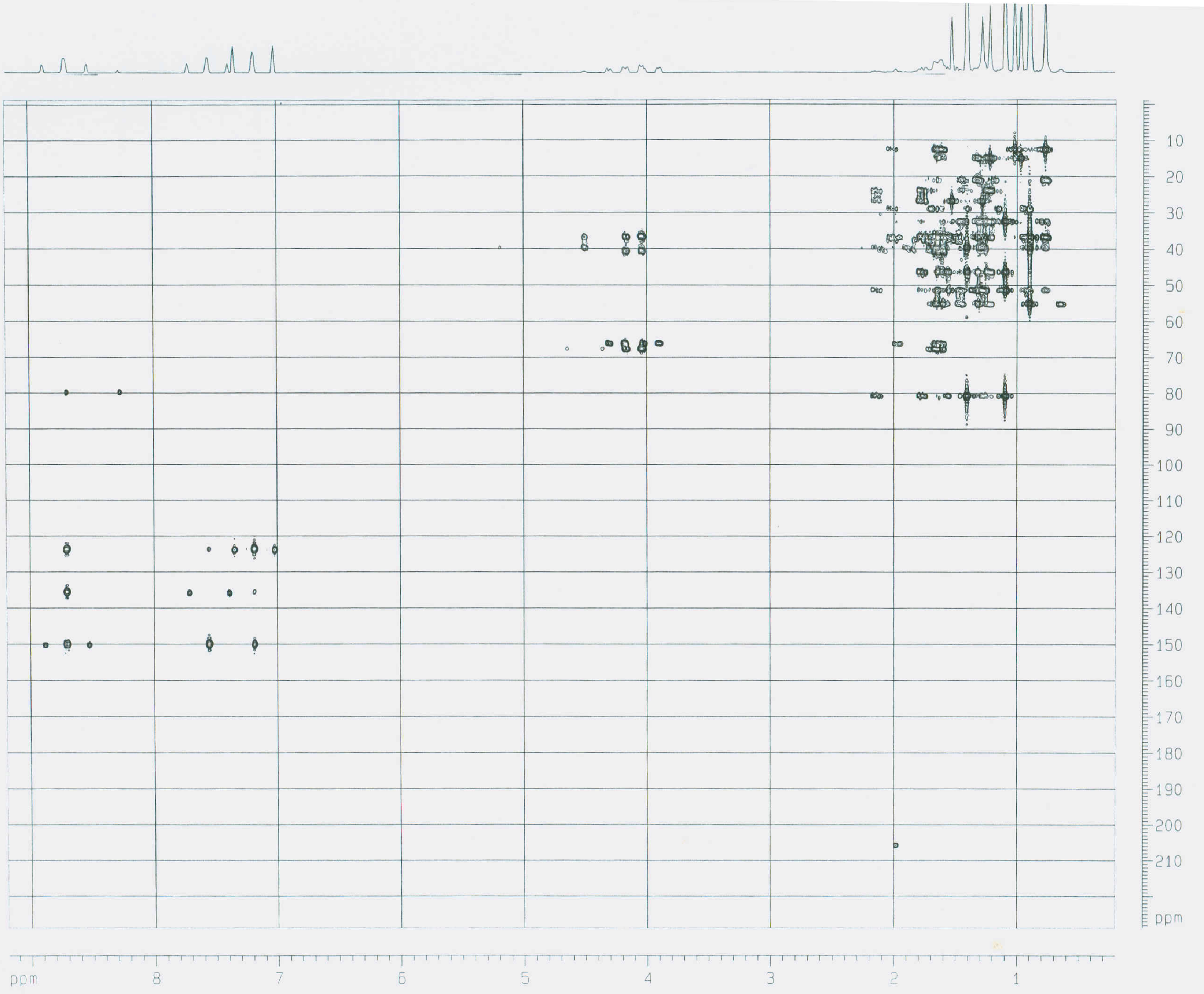

Current Data Parameters

NAME	oct31
EXPNO	5
PROCNO	1

F2 - Acquisition Parameters

Date_	20081031
Time	16.43
INSTRUM	spect
PROBHD	5 mm BBI 1H-BB
PULPROG	hmbcgpndqf
TD	4096
SOLVENT	Pyr
NS	64
DS	16
SWH	4496.403 Hz
FIDRES	1.097755 Hz
AQ	0.4556364 sec
RG	20642.5
DW	111.200 usec
DE	6.00 usec
TE	306.2 K
CNST13	8.0000000
d0	0.00000300 sec
D1	1.50000000 sec
d6	0.06250000 sec
D16	0.00010000 sec
IN0	0.00001728 sec
MCREST	0.00000000 sec
MCWRK	1.50000000 sec

======== CHANNEL f1 ========

NUC1	1H
P1	7.00 usec
p2	14.00 usec
PL1	0.00 dB
SFO1	500.3331020 MHz

======== CHANNEL f2 ========

NUC2	13C
P3	13.50 usec
PL2	-2.00 dB
SFO2	125.8225469 MHz

====== GRADIENT CHANNEL =====

GPNAM1	SINE.100
GPNAM2	SINE.100
GPNAM3	SINE.100
GPX1	0.00 %
GPX2	0.00 %
GPX3	0.00 %
GPY1	0.00 %
GPY2	0.00 %
GPY3	0.00 %
GPZ1	50.00 %
GPZ2	30.00 %
GPZ3	40.10 %
P16	1000.00 usec

F1 - Acquisition parameters

ND0	2
TD	256
SFO1	125.8225 MHz
FIDRES	113.060783 Hz
SW	230.035 ppm
FnMODE	QF

F2 - Processing parameters

SI	1024
SF	500.3307525 MHz
WDW	QSINE
SSB	8
LB	0.00 Hz
GB	0
PC	1.40

F1 - Processing parameters

SI	1024
MC2	QF
SF	125.8082360 MHz
WDW	QSINE
SSB	8
LB	0.00 Hz
GB	0

2D NMR plot parameters

CX2	20.00 cm
CX1	15.00 cm
F2PLO	9.189 ppm
F2LO	4597.70 Hz
F2PHI	0.202 ppm
F2HI	101.29 Hz
F1PLO	228.783 ppm
F1LO	28782.75 Hz
F1PHI	-1.278 ppm
F1HI	-160.81 Hz
F2PPMCM	0.44934 ppm/cm
F2HZCM	224.82013 Hz/cm
F1PPMCM	15.33739 ppm/cm
F1HZCM	1929.57056 Hz/cm

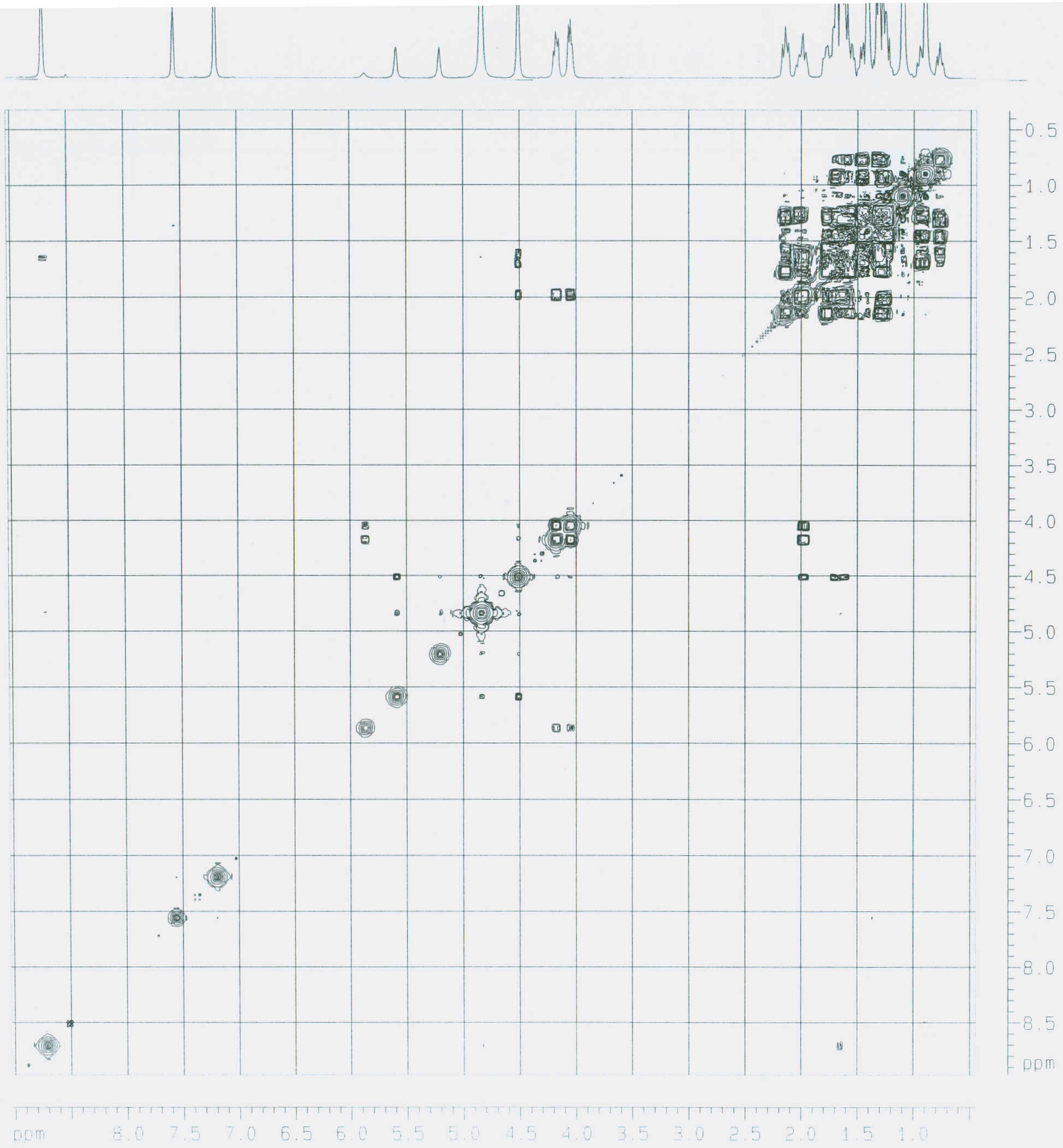

Current Data Parameters

NAME	oct31
EXPNO	2
PROCNO	1

F2 - Acquisition Parameters

Date_	20081031
Time	9.02
INSTRUM	spect
PROBHD	5 mm BBI 1H-BB
PULPROG	cosygf45
TD	2048
SOLVENT	Pyr
NS	16
DS	16
SWH	4496.403 Hz
FIDRES	2.195509 Hz
AQ	0.2278988 sec
RG	80.6
DW	111.200 usec
DE	6.00 usec
TE	304.6 K
d0	0.00000300 sec
D1	1.50000000 sec
IN0	0.00022240 sec
MCREST	0.00000000 sec
MCWRK	1.50000000 sec

======== CHANNEL f1 ========

NUC1	1H
P1	7.00 usec
PL1	0.00 dB
SFO1	500.3331020 MHz

F1 - Acquisition parameters

ND0	1
TD	128
SFO1	500.3331 MHz
FIDRES	35.128147 Hz
SW	8.987 ppm
FnMODE	QF

F2 - Processing parameters

SI	1024
SF	500.3307525 MHz
WDW	SINE
SSB	0
LB	0.00 Hz
GB	0
PC	1.40

F1 - Processing parameters

SI	1024
MC2	QF
SF	500.3307525 MHz
WDW	SINE
SSB	0
LB	0.00 Hz
GB	0

2D NMR plot parameters

CX2	15.00 cm
CX1	15.00 cm
F2PLO	9.031 ppm
F2LO	4518.66 Hz
F2PHI	0.448 ppm
F2HI	224.24 Hz
F1PLO	8.970 ppm
F1LO	4487.92 Hz
F1PHI	0.325 ppm
F1HI	162.77 Hz
F2PPMCM	0.57221 ppm/cm
F2HZCM	286.29440 Hz/cm
F1PPMCM	0.57631 ppm/cm
F1HZCM	266.34354 Hz/cm

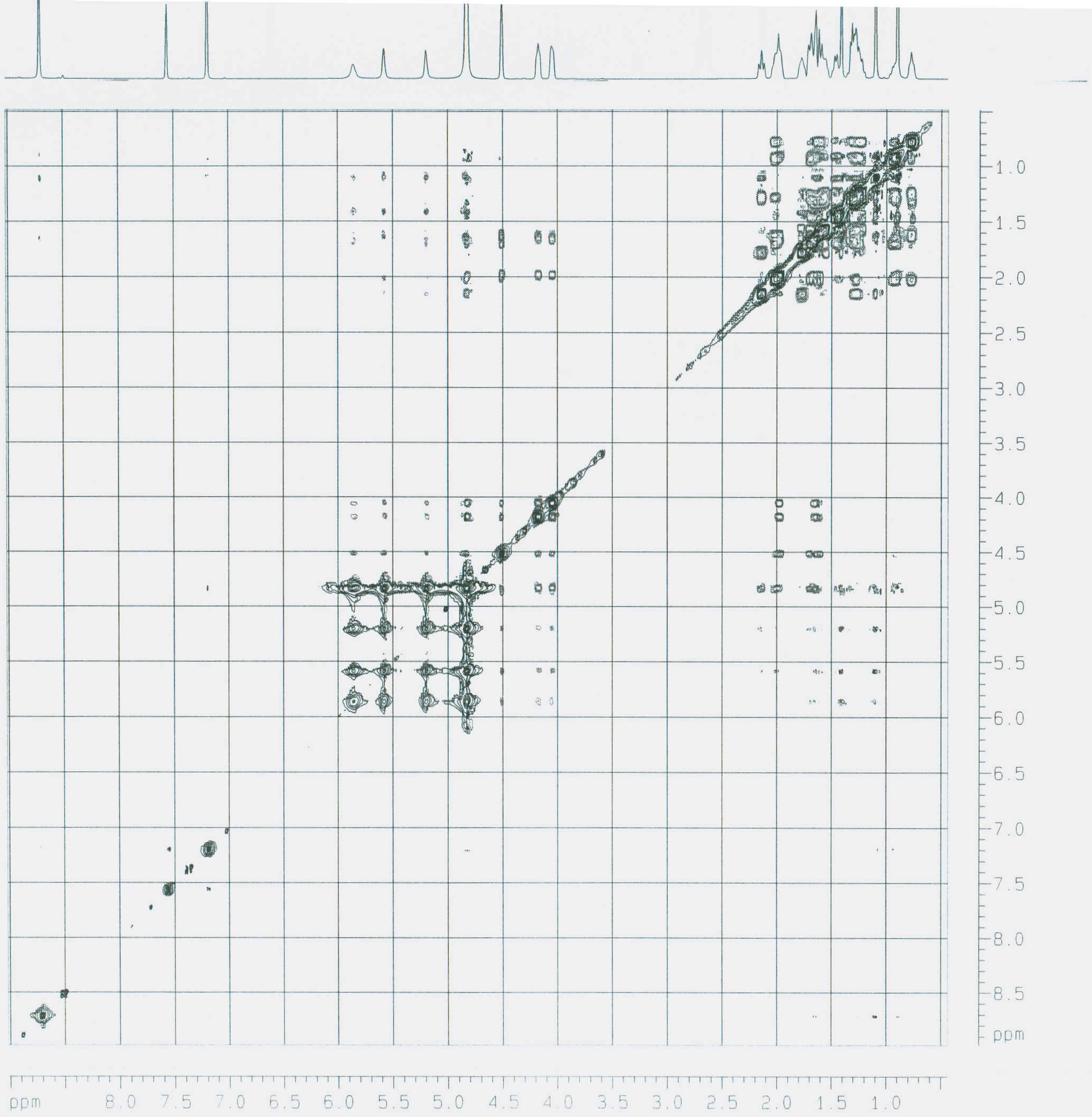

Current Data Parameters

NAME	oct31
EXPNO	3
PROCNO	1

F2 - Acquisition Parameters

Date_	20081031
Time	10.03
INSTRUM	spect
PROBHD	5 mm BBI 1H-BB
PULPROG	noesyph
TD	2048
SOLVENT	Pyr
NS	16
DS	16
SWH	4496.403 Hz
FIDRES	2.195509 Hz
AQ	0.2278988 sec
RG	128
DW	111.200 usec
DE	6.00 usec
TE	305.1 K
d0	0.00010229 sec
D1	1.50000000 sec
D8	0.80000001 sec
IN0	0.00022240 sec
MCREST	0.00000000 sec
MCWRK	0.75000000 sec
ST1CNT	128

======== CHANNEL f1 ========

NUC1	1H
P1	7.00 usec
PL1	0.00 dB
SFO1	500.3331020 MHz

F1 - Acquisition parameters

ND0	1
TD	256
SFO1	500.3331 MHz
FIDRES	17.564074 Hz
SW	8.987 ppm
FnMODE	States-TPPI

F2 - Processing parameters

SI	2048
SF	500.3307525 MHz
WDW	QSINE
SSB	2
LB	0.00 Hz
GB	0
PC	1.00

F1 - Processing parameters

SI	512
MC2	States-TPPI
SF	500.3307525 MHz
WDW	QSINE
SSB	2
LB	0.00 Hz
GB	0

2D NMR plot parameters

CX2	15.00 cm
CX1	15.00 cm
F2PLO	9.018 ppm
F2LO	4512.07 Hz
F2PHI	0.435 ppm
F2HI	217.66 Hz
F1PLO	8.979 ppm
F1LO	4492.31 Hz
F1PHI	0.483 ppm
F1HI	241.81 Hz
F2PPMCM	0.57221 ppm/cm
F2HZCM	286.29440 Hz/cm
F1PPMCM	0.56636 ppm/cm
F1HZCM	283.36703 Hz/cm

File: OX-1HP
Sample: NAIK
Instrument: JEOL JMS600
Inlet: Direct Probe

Date Run: 02-11-2009

Ionization mode: EI-

Time Run: 10:04:43

Run By: lab101
Printed by: lab101

Scan: 51
Base: m/z 261; 28.6%FS TIC: 4446638

R.T.: 2:06.8

#Ions: 182

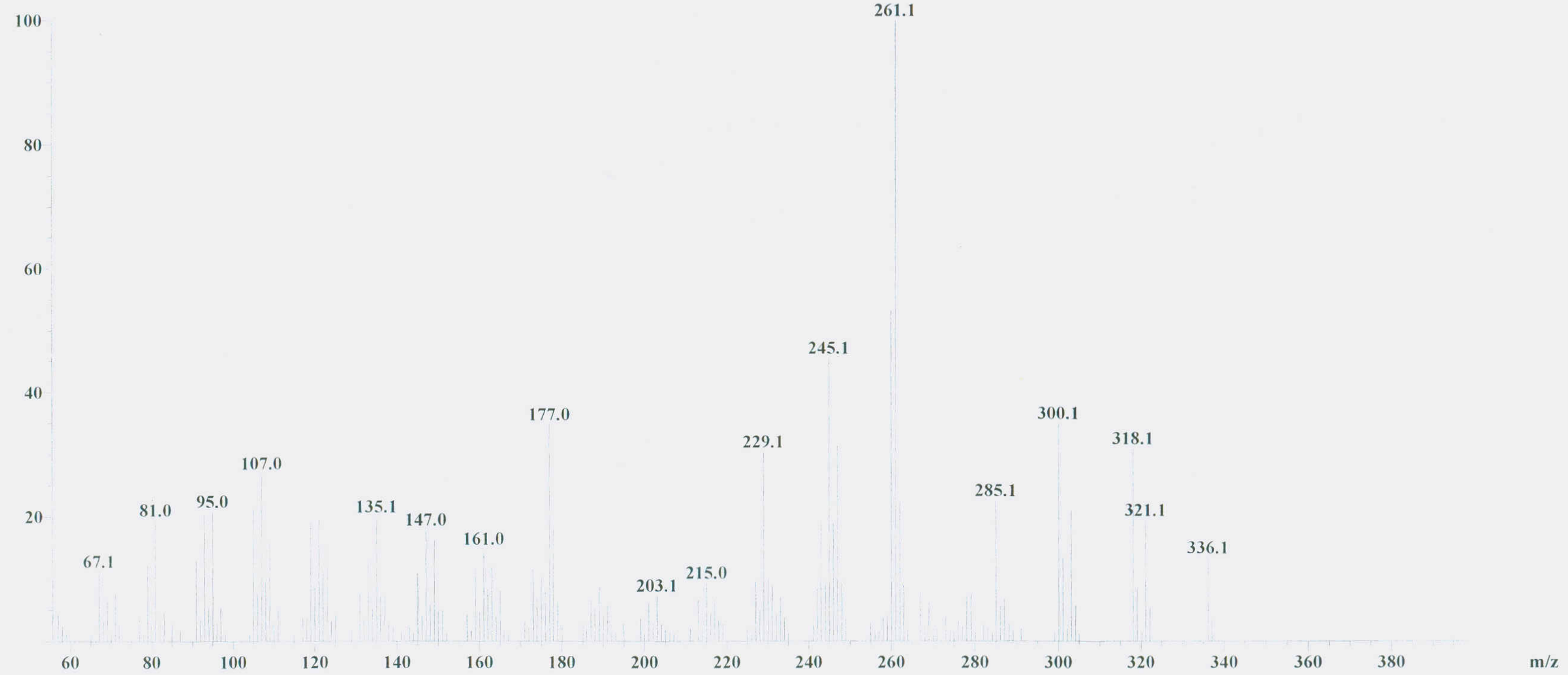

File: OX-1HP
Sample: NAIK
Instrument: JEOL JMS600
Inlet: Direct Probe

Date Run: 02-11-2009
Ionization mode: EI-

Time Run: 10:04:43
Run By: lab101
Printed by: lab101

Scan: 51
Base: m/z 261; 28.6%FS TIC: 4446638

R.T.: 2:06.8

#Ions: 182

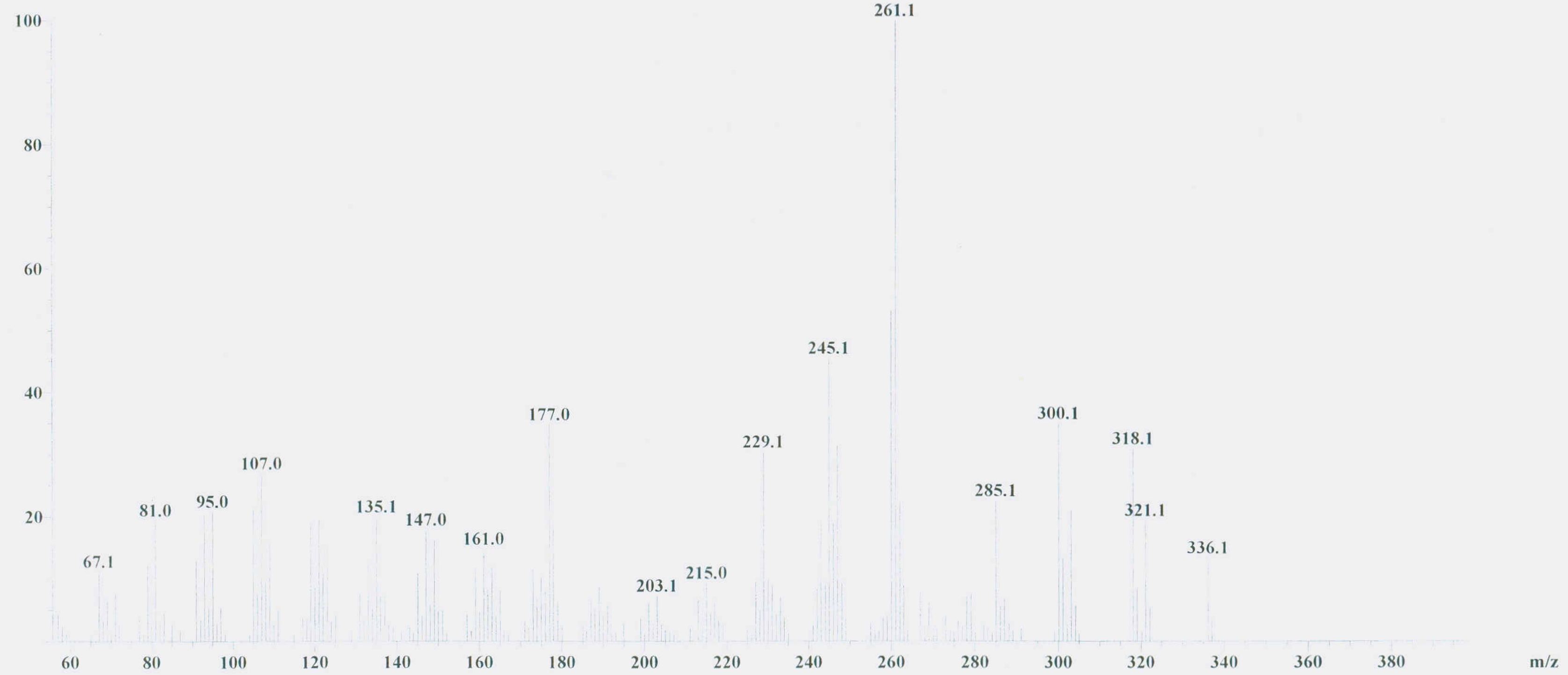

Supplement: Additional file 6 — Spectroscopic data of compound 7. Include spectra of 1H-NMR, 13C-NMR (BB, DEPT-135), HMQC, HMBC, COSY-45°, NOESY, and EI-MS. [file 1752-153X-6-153-S6.pdf]
